# Integrated strategies for enhancing agrifood productivity, lowering greenhouse gas emissions, and improving soil health

**DOI:** 10.1016/j.xinn.2025.101006

**Published:** 2025-06-25

**Authors:** Li Wang, Gina Marie Garland, Tida Ge, Shiqian Guo, Endalkachew Abebe Kebede, Chengang He, Mohamed Hijri, Daniel Plaza-Bonilla, Lindsay C. Stringer, Kyle Frankel Davis, Soon-Jae Lee, Shoujiang Feng, Li Wang, Zhenyang Wei, Hanwen Cao, Zhi Wang, Jiexiong Xu, Kadambot H.M. Siddique, Gary Y. Gan, Min Zhao

**Affiliations:** 1 College of Life and Environmental Science, Wenzhou University, Wenzhou 325035, China; 2 State & Local Joint Engineering Research Center for Ecological Treatment Technology of Urban Water Pollution, Wenzhou University, Wenzhou 325035, China; 3 Zhejiang Provincial Key Laboratory of Water Ecological Environment Treatment and Resource Protection , Wenzhou University, Wenzhou 325035, China; 4 Department of Environmental Systems Sciences, ETH Zurich, 8092 Zurich, Switzerland; 5 School of Integrative Plant Science, Cornell University, Ithaca, NY 14853, USA; 6 State Key Laboratory for Quality and Safety of Agro-Products, Key Laboratory of Biotechnology in Plant Protection of MARA, Institute of Plant Virology, Ningbo University, Ningbo 315211, China; 7 International Science and Technology Cooperation Base for the Regulation of Soil Biological Functions and One Health of Zhejiang Province, Ningbo University, Ningbo 315211, China; 8 Gansu Provincial General Station for Cultivated Land Quality Construction and Protection, Lanzhou 730030, China; 9 Department of Geography and Spatial Sciences, University of Delaware, Newark, DE 19716, USA; 10 College of Tobacco Science, Yunnan Agricultural University, Kunming 650031, China; 11 Institut de Recherche en Biologie Végétale, Département de Sciences Biologiques, Université de Montréal, Montréal, QC H1X 2B2, Canada; 12 African Genome Center, Université Mohammed VI Polytechnic (UM6P), Ben Guerir 43150, Morocco; 13 Department of Agricultural and Forest Science and Engineering – Agrotecnio-CERCA Center, Universitat de Lleida, 25198 Lleida, Spain; 14 York Environmental Sustainability Institute, and Department of Environment and Geography, University of York, York YO10 5DD, UK; 15 Department of Plant and Soil Sciences, University of Delaware, Newark, DE 19716, USA; 16 Department of Ecology and Evolution, University of Lausanne, 1015 Lausanne, Switzerland; 17 The UWA Institute of Agriculture, The University of Western Australia, Crawley, WA 6009, Australia; 18 Agroecosystems, The μBC-Soil Group, Tallus Heights, Kelowna, BC V4T 3M2, Canada

**Keywords:** agroecosystem resilience, alternative cropping systems, biofertilizers, biological nitrogen fixation, carbon footprint, N_2_O emissions, soil-plant-microbiome interactions

## Abstract

Global agrifood systems face three interconnected challenges: ensuring food security, promoting environmental sustainability, and restoring soil health in the face of climate change. Conventional practices have prioritized productivity over ecological resilience, leading to soil degradation, increased greenhouse gas (GHG) emissions, and inefficient resource utilization. Here, we introduce a “triple-goal” agrifood framework that enhances food production, soil health, and GHG mitigation simultaneously through integrated innovations. Using a second-order meta-analysis of 104 meta-analyses that cover 39,162 studies and 300,139 global field comparisons, we identified key interventions, including optimized fertigation, diversified cropping systems, organic amendments, and precision N management, that increased productivity by 14%–28% while reducing environmental impacts. Diversified systems boosted yields by 19.6% and reduced land use by 19%. Integrating legumes and cover crops lowered N_2_O emissions by 18%–65%, while organic amendments increased soil organic carbon stocks by 7%–13%. Structural equation modeling identified nitrogen use efficiency and microbial activity as central to the food-soil-emissions nexus. However, tradeoffs remain; yield-focused strategies can elevate emissions if not tailored to local conditions. By integrating agronomic, biological, and technological interventions such as conservation tillage, biofertilization, and digital agriculture, this triple-goal framework supports a 15%–30% reduction in anthropogenic CO_2_-equivalent emissions. These findings underscore the need for policy reform and multi-stakeholder collaboration to scale up the adaptation of integrated strategies in alignment with the UN’s Sustainable Development Goals and the “One Health” initiative. The triple-goal framework provides a transformative pathway to climate-smart, equitable, and resilient agrifood systems that strike a balance between productivity and planetary health.

## Public summary

• Integrated cropping strategies can simultaneously enhance food production, reduce emissions, and improve soil health.
• Enhancing plant-soil-microbe interactions can enhance agroecosystem resilience by 15%–40%.
• Prioritizing CO_2_ fertilization, along with biofertilization, can cut greenhouse gas emissions by 30%–50%.
• Legume-cereal intercropping can enhance system productivity while reducing environmental footprint.
• Second-order meta-analysis can synthesize comprehensive research to solve interlinked issues.

## Graphical abstract

**Figure:**
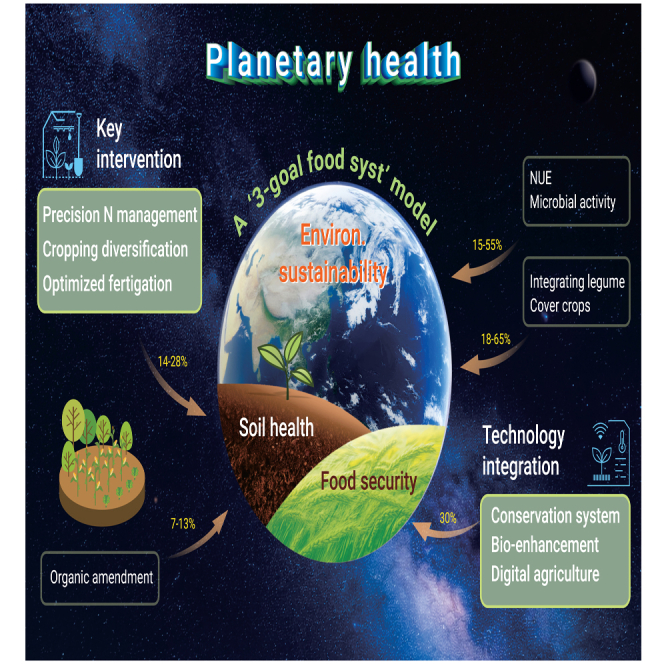

## Introduction

Since the Industrial Revolution (∼1850 CE), the Earth has undergone continuous warming^1^ profoundly affecting the atmosphere, hydrosphere, lithosphere, and biosphere.^2^ This change has intensified three interconnected global challenges—food security, environmental sustainability, and soil health—each of which occurs independently or often simultaneously.

(1) The food security challenge. Global food demand is projected to increase by 35%–56% between 2010 and 2050, aiming to meet nutritional needs and alleviate hunger. However, the risk of food insecurity varies widely during this period, ranging from −91% to +8%.^3^ The pressure on arable land is increasing, particularly in densely populated regions such as China, India, and many African nations.^4^ Rapid urbanization, industrial expansion, and ongoing land degradation continue to reduce cultivable land,^5^ further threatening food production.^6^ Converting carbon-rich grasslands and forests into croplands results in significant carbon losses^7^ and compromises agrifood system resilience.^8^ Global disruptions—including the COVID-19 pandemic, geopolitical conflicts, and restrictive trade policies—have also weakened supply chains^9^ and increased global food insecurity,^10^ underscoring the need to address the imbalance between food supply and demand.
(2) The environmental sustainability challenge. Since the Green Revolution of the 1950s–1960s, agriculture has increasingly relied on synthetic fertilizers,^11^ pesticides,^12^ and agrofuels.^13^ Synthetic nitrogen fertilizers in particular are a significant source of nitrous oxide (N_2_O),^14^ a potent and long-lived greenhouse gas (GHG). Over the past 40 years, nitrogen inputs to croplands have increased N_2_O emissions by approximately 30%,^15^ contributing to rising atmospheric GHG concentrations.^16^ Globally, food systems emit about 20 Gt CO_2_ equiv year^−1^, about 35% of total GHG emissions,^17^ with agricultural production accounting for about half of all non-CO_2_ emissions between 1980 and 2016.^15^ For instance, wheat—a staple food crop—will likely require significant nitrogen inputs to realize more than 50% of its yield potential in a warming climate,^11^ inevitably increasing environmental burdens and highlighting the urgent need to reduce GHG emissions and work toward net-zero agriculture to help reverse climate change.^18^
(3) The soil health challenge. Many unsustainable farming practices have severely degraded soil health.^19^ Conventional tillage disrupts soil organic carbon (SOC) accumulation and mineralization, thereby weakening soil structure and fertility.^20^ Frequent soil disturbance destroys soil aggregates, reducing carbon stability,^21^ while continuous monoculture disrupts microbial communities and biodiversity.^22^ Excessive use of synthetic agrochemicals further deteriorates the chemical and biological integrity of soil,^23^ leading to soil salinization,^24^ acidification,^25^ and nutrient imbalances^26^ as well as water pollution^27^ and habitat destruction.^28^ These factors have negatively affected soil health, while crop yields dependent on nitrogen inputs have stagnated^29^—or even declined—in some regions.^30^ Although countries like China and India have achieved substantial yield gains in recent decades, those improvements have often come at the expense of soil health.^31^ Globally, an estimated 24 billion tons of fertile soil are lost annually, and over 90% of the Earth’s land may be degraded by 2050 if current trends continue.^32^

**Figure 1:**
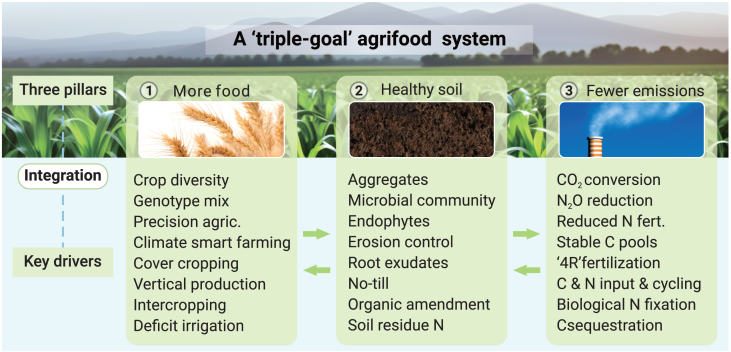
Integration of the three pillars—more food, healthier soils, and fewer emissions—within the triple-goal agrifood framework Each pillar is supported by key drivers. (A) More food through innovative and sustainable practices such as alley cropping, intercropping, genotype diversification, deficit irrigation, cover cropping, legume-based rotations, smart farming, precision agriculture, and vertical farming. (B) Healthier soils through strategies including increased carbon inputs, stable carbon pool formation, soil amendments, reduced or no-till practices, enhanced carbon and nitrogen cycling, improved soil aggregation, stimulation of root exudation, and promotion of endophyte activity. (C) Fewer emissions through enhanced carbon sequestration, reduced or no-till practices, optimized fertilization, improved residue N management, 4R fertilization strategies,^19^ erosion control, and management practices to reduce N_2_O emissions.

To address these interlinked challenges—feeding a growing population, reducing environmental impacts, and restoring degraded soils—we introduce a “triple-goal agrifood production framework” (hereafter called the triple-goal framework). This integrated, multi-factor approach, synthesizing improved and emerging agricultural practices, is built on three foundational pillars, each supported by key drivers (Figure 1). The triple-goal framework features the following: (1) enhancing system resilience by integrating existing and novel technologies that improve plant-soil-microbe-environment interactions; (2) promoting carbon source-to-sink strategies that boost soil carbon sequestration and support global carbon cycling; (3) stimulating biological processes—including enzymatic and microbial activities—to enhance water and nutrient use efficiency, thereby improving soil biochemical properties; and (4) reducing reliance on synthetic nitrogen by leveraging biological nitrogen fixation (BNF), thereby decreasing nitrogen fertilizer inputs and lowering N_2_O emissions. The triple-goal framework is based on a comprehensive synthesis of findings from 104 individual meta-analyses (Table S1), incorporating studies from countries such as Australia, Canada, China, the United States, the United Kingdom, European Union (EU) member states, and others worldwide.

### Second-order meta-analysis

In the study, we employed a second-order meta-analysis (SOMA)^33^—similar to the approach used by Beillouin et al.,^23^ Xu et al.,^34^ and Ascenzi et al.^35^—to synthesize findings across multiple, individual first-order meta-analyses. We defined our target subject area and identified 104 relevant first-order meta-analyses, collectively encompassing 39,162 studies (or experiments) and 300,139 paired comparisons between target treatments and the control groups (Table S1). These articles were selected based on predefined criteria and a structured selection process (Table S2). The geographic distribution of study sites is shown in the global map (Figure S1). The rationale for adopting SOMA in this study, along with its robustness, is provided in the supplemental information.

From the selected 104 meta-analyses, we extracted all effect sizes (e.g., mean differences, odds ratios, Cohen’s *d*, Hedges’ *g*, and Pearson correlation *r*), their variances (standard errors and confidence intervals), and the number of primary studies and observations. To ensure consistency in the SOMA metrics, we converted all the effect sizes to Hedges’ *g* (a bias-corrected standardized mean difference) using the following formulas:

(Equation 1) $$g=\left(1-\frac{3}{4*df-1}\right)\times d$$

(Equation 2) $$d=\frac{{2r}_{xy}}{\sqrt{1-{r_{xy}}^{2}}}$$

where *g* is Hedges’ *g*, *d* is Cohen’s *d*, and *r* is the Pearson x∗y correlation coefficient). We employed a random-effects model of comprehensive meta-analysis (CMA)^36^ and calculated the summary effect size and its variance using the following formulas:

(Equation 3) $$M=\frac{\sum_{i-1}^{k}W_{i}Y_{i}}{\sum_{i-1}^{k}W_{i}}$$

(Equation 4) $$V_{M}=\frac{1}{\sum_{i-1}^{k}W_{i}}$$

where *M* is the summary effect size, $V_{M}$ is the variance of the summary effect, $W_{i}$ is the weight for study *i*, which was computed by the inverse of the sum of the within-study variance for study *i* and between-study variance, and $Y_{i}$ is the effect size for study *i*. We retained independent effect sizes and weighted them by the inverse of their variance, as recommended by Sanchez-Meca and Marín-Martínez,^37^ to reduce the influence of lower-quality meta-analyses. Furthermore, we quantified the heterogeneity of true effect sizes using CMA’s built-in statistics, including Q value, *T*^2^, and *I*^2^. The Q statistic and its *p* value test the null hypothesis that the true effect sizes are consistent across studies. *T*2 is the estimate of the variance of the true effects, and *I*^2^ is an index indicating the percentage of variability that reflects the heterogeneity of true effect sizes.

The CMA calculated the Q statistic and *I*^2^ to assess heterogeneity and the significance of $\tau^{2}$:

(Equation 5) $$I^{2}=\frac{\tau^{2}}{\tau^{2}+⩢}\times 100\%$$

(Equation 6) $$Q=\sum w_{j}{\left(g_{j}-\mu \right)}^{2}$$

where ⩢ is the average within the meta-analysis variance, and *w*_*j*_ is the inverse variance weight.

From the SOMA modeling, we identified relevant drivers from a list of predefined soil- and crop-related anthropogenic activities. We then categorized the effect sizes for 6–8 key drivers across the three goal areas: increased food production, healthier soils, and reduced emissions. More detailed descriptions of the SOMA methodology, statistical models, publication bias, and limitations are provided in the supplemental information.

### The boundaries

The triple-goal framework focuses exclusively on staple and vegetable crops, addressing three key pillars simultaneously—more food, healthier soils, and reduced emissions (Figure 1). Other food sectors, such as fisheries, livestock, synthetic foods, and agroforestry, fall outside the scope of this study. Although these sectors may offer income opportunities for smallholder farmers in Africa,^38^ they are beyond the objectives of this analysis. Similarly, while many publications explore soil- and crop-specific agronomic practices for particular cropping systems, consolidating such practices by cropping system was not the aim of this work.

## Pillar 1: More food

### Core strategies for boosting agrifood production

**Figure 2:**
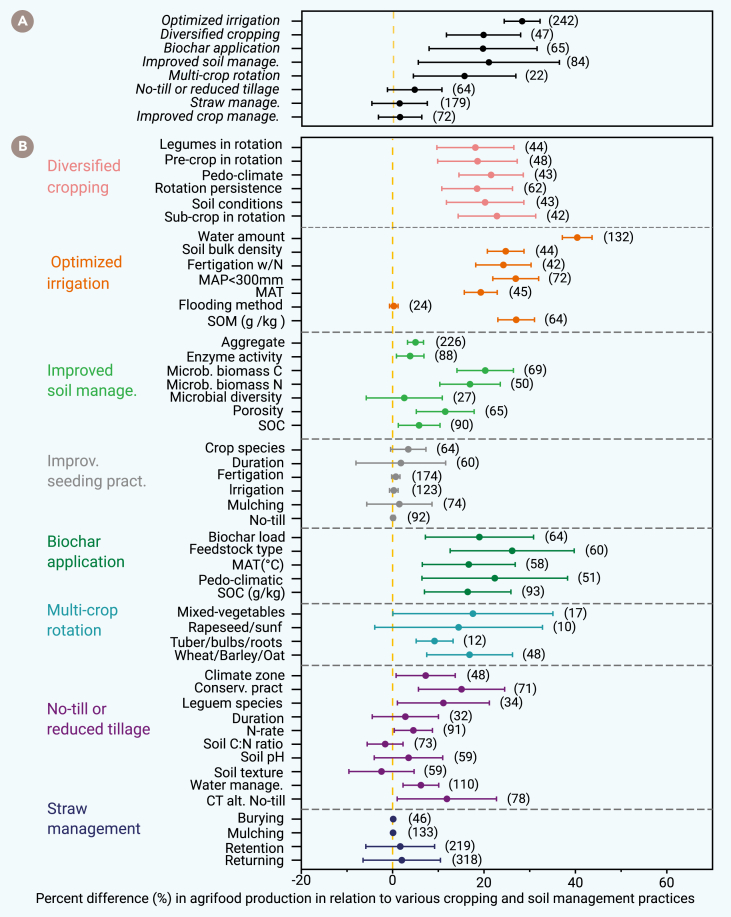
The triple-goal framework integrates established and emerging farming practices to maximize agrifood productivity and stability (A) Results from the SOMA indicate that integrated farming approaches significantly increase crop yields compared to conventional practices. The main contributors to yield gains are optimized irrigation (28.3% increase, *n* = 60 first-order meta-analyses), diversified cropping systems (19.6%, *n* = 47), organic amendments (19.4%, *n* = 65), and improved soil management (18.8%, *n* = 84). (B) Each of these key drivers comprises a range of agronomic practices, leading to varying effects on crop yields. While multi-crop rotation, reduced or no tilling, crop seeding practices, and straw management also improved yields, they generally had smaller effects.

The triple-goal framework integrates innovative, proven strategies to enhance agrifood productivity, including optimized irrigation, diversified cropping systems, biochar application, and improved soil and crop management (Figure 2). The SOMA showed that optimized irrigation increased agrifood production by 28.3% (*n* = 60 first-order meta-analyses), followed by diversified cropping systems by 19.6% (*n* = 47), organic amendments like biochar by 19.4% (*n* = 65), and improved soil management by 18.8% (*n* = 84), with each effect size weighted by the number of contributing studies or experiments. Multi-crop rotation also increased crop yield by 14.7%, whereas reduced or no-till practices and straw management had comparatively smaller gains. Returning crop residue to the soil as compost or biochar promotes microbial activity, which improves soil nitrogen and boosts production.^39^^,^^40^ Structural equation modeling further supports the notion that productivity gains are strongly linked to the total nitrogen supplied and nitrogen use efficiency (NUE), which, in turn, are associated with plant nitrogen uptake and soil nitrogen accumulation (Figure S2). However, the effects of biochar on soil nutrients and crop production vary widely depending on soil biochemical properties (e.g., pH and N status),^41^ crop species,^42^ biochar properties,^43^^,^^44^ and application methods and rates.^44^^,^^45^ In the short term (≤5 years), crop yields following biochar application can fluctuate due to interannual variability in temperature and precipitation, although such variability tends to diminish over time.^46^

Traditional agrifood production systems, which rely on monoculture in wheat (*Triticum aestivum* L.), rice (*Oryza sativa* L.), maize (*Zea mays* L.), canola (*Brassica juncea* L.), and other staple crops, face limitations due to socioeconomic barriers and saturated markets focused on caloric and oil products. In contrast, diversified cropping systems—where crops with contrasting morphological, physiological, or biochemical traits are grown together—offer a promising alternative. Diversification may occur across time (e.g., crop rotations), space (e.g., intercropping), or both. For example, southern China supports intensive double or triple cropping due to its favorable climate; the EU commonly uses cover crop-cash crop rotations, and the semiarid northwestern plains of India practice cereal-legume intercropping.^47^ These locally adapted approaches can improve food security,^48^ enhance profitability, and reduce nitrogen-induced GHG emissions.^49^

Intercropping systems combining forage, grain, oilseed, and legumes in strip,^50^ alley,^51^ or relay^52^ formats improve both productivity and resource use efficiency.^53^ One meta-analysis of 88 studies found that maize-soybean (*Glycine max* L.) intercropping improved nitrogen and phosphorus use efficiencies compared to monoculture.^54^ Another meta-analysis (226 experiments and 934 observations) found that yield gains from intercropping were equivalent to a 19% land saving compared to sole cropping.^55^ These benefits grow with continued use over time due to improved soil fertility^56^ driven by enhanced root exudates and nitrogen fixation by legumes.^57^ Many other soil and crop management practices have also demonstrated significant advantages over monoculture in supporting the triple-goal framework (Table S3).

### System resilience: Insurance for agrifood security

Agrifood production systems are highly vulnerable to disruptions and shocks caused by geopolitical crises (e.g., the Russia-Ukraine war), public health emergencies (e.g., the COVID-19 pandemic), and natural disasters (e.g., severe droughts), which can significantly impact supply chains at local to global scales.^58^ The triple-goal framework seeks to enhance production system resilience, enabling it to tolerate moderate abiotic and biotic stresses while ensuring rapid recovery once disruptions subside.

Diverse cropping systems play a key role in this resilience by disrupting host plant-pest species-environment relationships, thereby reducing the survival of pathogens with narrow host ranges and short life cycles.^59^^,^^60^ Spatiotemporal crop diversity hinders the establishment of host-favoring fungal microbiomes.^61^ Host plants influence microbiome composition through the selective allocation of resources to multiple symbionts,^62^ which can further limit pathogen resistance. These practices also help address the growing challenge of pesticide resistance, particularly in regions where excessive pesticide use became widespread following the Green Revolution. Well-designed, diversified systems that incorporate crops with diverse growth habits, life cycles, and morphologies can effectively suppress pest populations. Rotating between cool- and warm-season crops, annuals and perennials, and monocots and dicots disrupts pest life cycles and reduces their persistence.^63^ Additionally, using multiple modes of pesticide control (e.g., herbicidal and non-herbicidal strategies) can delay resistance development, supporting long-term crop health.^64^ A resilient agrifood production system must strike a balance between production and risk mitigation.^65^ One key approach is reducing synthetic nitrogen fertilizer use, a major contributor to N_2_O emissions and the nitrogen-induced carbon footprint.^66^ Applying organic fertilizers, integrating a broader array of nutrients, and fostering plant-microbe compatibility can reduce N_2_O emissions while maintaining or improving productivity; these measures contribute to a more robust and lower-risk agrifood system.

### Agrifood challenges following the Green Revolution

A significant challenge facing agrifood systems in the 21^st^ century is the persistent issue of nutritional inequality. According to the Food and Agriculture Organization of the United Nations （FAO） Food Security and Nutrition Report, more than 720 million people were undernourished following the COVID-19 pandemic—an increase of more than 150 million since 2019.^67^ Ongoing global uncertainties have further disrupted food supply chains, triggering significant inflation in commodity prices. Countries heavily reliant on agricultural imports, such as those in the Middle East and North Africa, have been particularly affected.^68^ Compounding these issues, extreme weather events—such as droughts and floods—continue to place additional pressure on already vulnerable agrifood systems.

While the Green Revolution significantly boosted calorie production—particularly from rice, wheat, and maize—it also contributed to rising nutrient deficiencies in developing nations and increasing overweight and obesity rates in the developed world.^69^ Malnutrition remains a widespread public health challenge in sub-Saharan Africa,^70^ where the Green Revolution had a limited impact on marginal lands.^71^ In Southeast Asia, smallholder farms gained few benefits due to inequitable land ownership, unaffordable inputs, and policies that marginalized small-scale producers.^30^^,^^72^

In Africa, abundant arable land and underdeveloped market infrastructure hindered the effectiveness of the Green Revolution.^71^ A shift toward regionally adapted staple crops such as millet (*Cenchrus americanus* L.), sorghum (*Sorghum bicolor* L.), and cassava (*Manihot esculenta* L.) could offer greater nutritional benefits.^73^ Furthermore, the social aspects of agricultural development were often overlooked in many developing countries during the Green Revolution,^74^ leading to class and gender disparities.^75^ Women-headed households, in particular, have faced lower crop yields and incomes, leaving them more vulnerable to climate change and economic shocks. Addressing these gender-specific vulnerabilities requires improving women’s access to markets, promoting labor-saving technologies, and supporting women’s organizations.^73^ We advocate for coordinated action by governments, international organizations, and local communities to address the legacy shortcomings of the Green Revolution. A sustainable transformation of global agrifood systems must prioritize social equity, ensuring that smallholder and marginalized farmers have fair access to agricultural innovations and resources.^76^

## Pillar 2: Healthier soil

Soil health, defined by the synergistic interaction of biological, physical, and chemical properties,^19^ is fundamental for sustaining long-term agrifood productivity. Soils host an astonishing abundance and diversity of life, including earthworms, nematodes, mammals, insects, and microorganisms.^77^ A single gram of soil can contain up to 10^11^ billion bacteria,^78^ with soils home to approximately 59% of Earth’s species, making them the most diverse habitat on the planet.^79^ Recent advances in DNA sequencing and metagenomics have deepened our understanding of soil microbial communities, which are key drivers of essential ecological functions. These microbiomes participate in SOC decomposition by releasing hormones and chemical compounds, helping store around 1,325 Pg organic carbon within the top 100 mm of soil.^80^ The balance between carbon released into the atmosphere through respiration and carbon stored in the soil through sequestration largely determines the size of soil carbon pools. Even small shifts in this balance can have a significant impact on overall carbon dynamics.^81^

**Figure 3:**
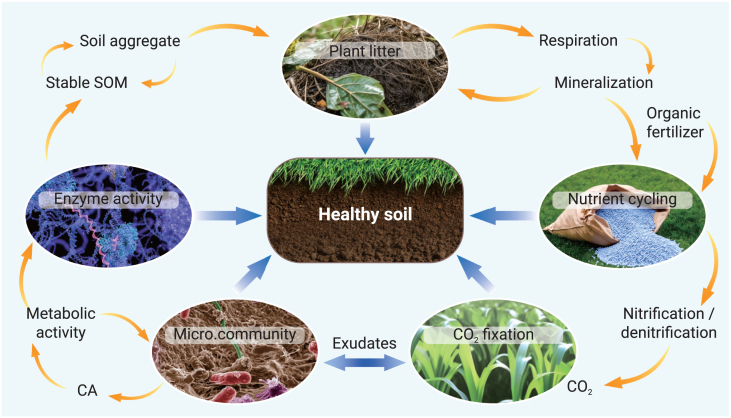
A healthy soil system involves complex metabolic pathways, nutrient transfer and cycling, and dynamic enzymatic and microbial activities Continuous inputs of plant litter and organic fertilizers contribute to maintaining stable soil organic matter (SOM) and improving soil structure through enhanced aggregation, which physically protects carbon pools from degradation and promotes microbial growth and activity. The coordination of (de)nitrification metabolic activities and processes involving in soil respiration and mineralization, driven by enzyme activities and root exudates, plays a key role in nutrient cycling. Optimized soil and crop management strategies can enhance soil health under favorable soil and climatic conditions.

Within this context, our triple-goal framework includes several strategies to optimize soil health (Figure 3). These strategies focus on balancing plant litter input, soil aggregates, and soil organic matter stability, regulating carbon loss through respiration and mineralization, managing organic fertilizer input and nutrient cycling, controlling (de)nitrification and CO_2_ fixation, and fostering microbial community diversity and metabolic activity. Exudates and enzyme activities are crucial intermediaries that link microbial communities to soil structure.

**Figure 4:**
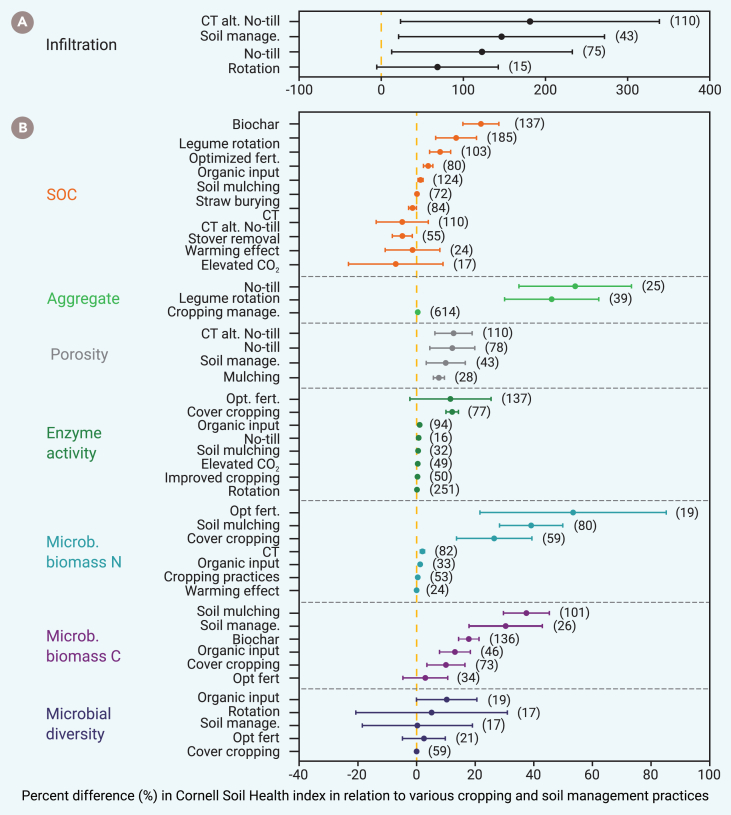
Key driving factors impacting soil health (A) The SOMA revealed that soil infiltration is the most critical driver impacting soil health, as indicated by the Cornel Soil Health Index. Soil infiltration is closely related to tillage, crop rotation, and other soil management practices. (B) Various anthropogenic activities impact soil health by altering soil physiochemical and biological properties, including SOC (SOC), aggregate stability, soil porosity, enzymatic activity, microbial biomass carbon (MBC), microbial biomass nitrogen (MBN), and microbial diversity. Each of these seven soil properties is influenced by different soil- and crop-related practices.

Our SOMA of original meta-analyses indicates that soil bio-physiochemical properties and anthropogenic activities influence soil health (Figure 4). Increased soil infiltration enhances the Cornell Soil Health Index^31^ by 127% on average (*n* = 61 meta-analysis studies). Alternating conventional tillage with no-till methods contributes most to improved soil infiltration, followed by soil management and crop rotations (Figure 4A). Other soil properties that positively impact the Cornell Soil Health Index include soil aggregates (45%, *n* = 69), microbial biomass carbon (MBC) (20%, *n* = 69), microbial biomass nitrogen (MBN) (17%, *n* = 50), and soil porosity (11%, *n* = 65) (Figure 4B). Other soil factors, such as SOC, enzymatic activity, and microbial diversity, all showed positive but narrower effects on the soil health index (3.1%–5.6%). Key anthropogenic activities impacting soil health include crop cultivation, optimized fertilization and irrigation (e.g., fertigation), and improved cropping practices.

**Figure 5:**
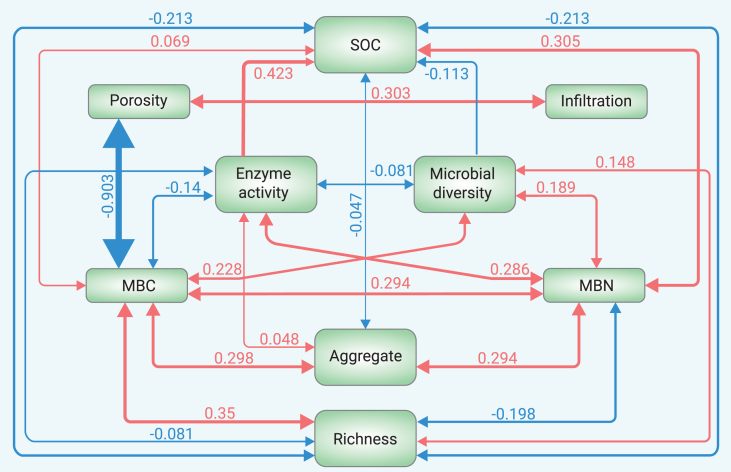
Structural equation modeling demonstrates that many soil properties exhibit significant interactions Notably, there are highly significant, positive relationships between enzyme activity and soil organic carbon (SOC), MBC and MBN, MBC and microbial richness, MBN and SOC, MBN and porosity, MBN and infiltration, and MBN and enzymatic activities. Less important factors to the triple-goal framework were excluded based on their correlation coefficients (indicated by the numbers beside the corresponding lines).

Structural equation modeling revealed complex relationships between soil properties, both positive and negative (Figure 5). Strong positive correlations exist between enzymic activity and SOC, MBC and MBN, MBC and microbial richness, MBN and SOC, MBC and porosity, and MBC and aggregation. These relationships highlight the importance of refining cropping and soil management practices to improve soil health, with the strength of these correlations varying according to local conditions.

### Managing soil carbon to enhance soil health

Stable SOC within aggregates is a significant nutrient reservoir that enhances the soil’s buffering capacity.^82^ Numerous anthropogenic activities influence SOC accumulation, transportation, and decomposition, thereby affecting the size and stability of SOC pools. A meta-analysis of 269 studies, encompassing 2,035 observations, showed that adding organic materials, such as biochar, manure, and crop straw, increased aggregate-protected carbon by 21%–34% and aggregate stability by 19%–23%, thereby reducing the decomposability of aggregate-associated organic carbon.^83^ Applying nature-based organic amendments to soils in arid and semiarid climates lowered soil temperatures, protected soil aggregates, and enhanced stable carbon storage. Improved cropping systems—such as cover cropping and agroforestry—also substantially increased stable SOC stocks. A global meta-analysis of 434 paired observations^84^ found that cover cropping increased SOC stocks at 60% of the study sites, particularly where initial SOC concentrations were below 11.6 g kg^−1^. These findings highlight that anthropogenic management is crucial for enhancing SOC stocks, with aggregate-protected carbon pools being key repositories for long-term carbon sequestration and improved soil health.

SOC plays a crucial role in shaping the soil’s physicochemical properties. While mineralogy and texture largely determine the baseline SOC content,^85^ agricultural practices can significantly modify SOC levels, influencing bulk density, cation exchange capacity (CEC), and the soil’s potential for carbon sequestration. For instance, increasing organic matter inputs enhances SOC concentrations by forming stable complexes with soil minerals. Higher SOC levels improve bulk density and CEC, enhancing soil structure and overall functionality. The soil carbon-to-nitrogen ratio influences carbon assimilation and emissions due to the positive association between SOC decomposability and CO_2_ cycling.^86^ In conjunction with local climate conditions, SOC provides critical habitats for microbial biodiversity, which supports nutrient cycling, water infiltration, and the breakdown of contaminants.

In agrifood systems, SOC accumulation is driven by inputs from root exudates, plant residues, and microbial necromass. A net sequestration rate of 2.1 Mg C ha^−1^ year^−1^ is required to counterbalance global soil carbon losses. Projections suggest that annual inputs of 5.1 Mg C ha^−1^ could raise SOC stocks to 55 Mg C ha^−1^ by 2050.^87^ However, SOC stability is often undermined by the priming effect, whereby fresh organic inputs stimulate native SOC mineralization. Meta-analyses indicate that exogenous carbon amendments can increase native SOC decomposition by up to 61% in croplands,^88^ with priming intensity governed by SOM recalcitrance and mineral-organic interactions.^89^

The soil microbiota plays a central role in carbon cycling, performing key metabolic functions. Anabolic processes lead to the synthesis of complex organic compounds, while catabolic processes mineralize SOC, releasing energy-rich byproducts such as pyruvate and ethanol, along with secondary metabolites that influence soil food web dynamics.^90^ Microbial residues represent a diverse and significant fraction of stable SOC, potentially up to 50%,^91^ forming persistent organic pools and highlighting microbial metabolites as accumulators critical to long-term carbon storage and microbially derived carbon stability. Globally, soils release an estimated 75–100 Pg CO_2_ annually through respiration,^92^ reflecting the diverse metabolic pathways of different decomposers: bacteria dominate plant residue breakdown,^93^ archaea drive methanogenesis in anoxic environments, and fungi specialize in degrading recalcitrant compounds such as lignin and cellulose.^91^ Some specialized soil fungi can also decompose the carcasses of insects and earthworms. Additionally, protozoa influence carbon cycling by selectively grazing on microbial populations, thereby modulating decomposition dynamics.^92^

### Promoting microbial community functioning to enhance soil health

The increasing recognition of the critical role of soil biodiversity in ecosystem functioning has intensified efforts to develop strategies that assess the contributions of distinct soil biological groups. These strategies are vital for conserving habitats and sustaining soil ecosystem health. Soil microbial communities exhibit uneven spatial and temporal distributions,^94^ with their biogeochemical impacts varying significantly at global, regional, and field scales. At broader scales, climate, parent material, and topography shape the underlying soil physicochemical and structural properties, which govern microbial community composition and functional potential.^77^ Even within a single soil type, microscale variations in structure and chemistry—such as differences in pore size, aggregate distribution, and root architecture—can increase microbial diversity by affecting oxygen levels, water availability, and nutrient dynamics.^95^ Microbial populations are primarily concentrated in the rhizosphere, where root exudates and decaying roots provide readily available carbon sources.^96^ This spatial variability renders microbial communities highly responsive to land use and management practices. Temporal drivers, such as seasonal cycles and microclimate fluctuations, further influence microbial abundance, composition, and diversity.

Soil microbes play a vital role in decomposing plant biomass into SOM, stabilizing it by forming organo-mineral complexes,^97^ sequestering it within soil aggregates,^98^ or mineralizing it and releasing CO_2_ into the atmosphere.^92^ As key regulators of carbon cycling, soil microbes play a central role in maintaining ecosystem services, particularly in agricultural systems. Unlike natural ecosystems, where microbial communities are shaped predominantly by inherent soil properties, climate, and vegetation, managed agricultural systems allow for deliberate interventions that influence microbial dynamics. Increasing soil carbon inputs not only compensates for carbon losses due to harvesting but also contributes to climate change mitigation by promoting the formation of stable carbon pools and enhancing microbial resilience.^99^ Strategic adjustments to management practices can result in long-term improvements in soil properties,^100^ reduce anthropogenic carbon emissions,^101^ and strengthen soil health and climate resilience. Fostering healthy and diverse microbial communities in soil contributes to more sustainable agriculture and global food security.

### Managing nitrogen to enhance soil health

Soil nitrogen availability is governed by three interrelated sources: BNF, organic nitrogen mineralization from decomposing plant residues, and atmospheric nitrogen deposition. Among these, BNF can contribute up to 70% of the aboveground plant nitrogen in legume-dominated systems, highlighting its potential for reducing the reliance on synthetic nitrogen fertilizers. The triple-goal framework encourages the use of nitrogen-fixing microbial technologies, such as *Rhizobium* and arbuscular mycorrhizal (AM) fungal inoculants, to enhance nodulation and phosphorus uptake, thereby improving symbiotic nitrogen fixation. At the same time, the mineralization of organic nitrogen—regulated by soil moisture and temperature, physicochemical properties, and functional microbial communities—supplies plant-available ammonium (NH_4_^+^), which can then be nitrified into nitrate (NO_3_^−^) or denitrified into nitrous oxide (N_2_O) and dinitrogen (N_2_).^102^ Specialized microbial consortia mediate these transformations, facilitating the flow of nitrogen between organic matter (e.g., crop residues and manure) and plant-accessible forms.

The triple-goal framework promotes (1) precision management practices, such as the 4R approach to fertilization^19^ (applying the right source, at the correct rate, at the right time, and in the right place), to maximize plant nitrogen uptake and minimize excess inorganic nitrogen accumulation; (2) crop residue retention and the use of organic amendments to stabilize nitrogen pools;; and (3) innovative interventions such as biochar, nitrification inhibitors, and slow-release fertilizers to modulate key biochemical processes and reduce N_2_O emissions. Importantly, the effectiveness of these strategies is highly context dependent, shaped by controllable factors (e.g., crop genotype and tillage) and uncontrollable variables (e.g. and climatic extremes).^102^ Therefore, integrated nitrogen management must remain adaptive and site specific to support soil health and agricultural sustainability.

### Managing the microenvironment to enhance soil health

Soil microenvironments that support microbial activity are fundamental to driving nutrient cycling and detoxification processes. Extracellular enzymes play a central role in these functions, playing key roles in nutrient turnover^103^ and SOM mineralization,^104^ and are well-recognized soil health indicators. Their activity—directly linked to substrate availability—reflects microbial metabolic potential and serves as a biomarker of soil functionality.^19^ Beyond decomposing SOM to fuel microbial biomass, these enzymes mediate nutrient transformations, break down contaminants such as heavy metals and microplastics, and suppress soil-borne pathogens. Notably, synergistic plant-microbe interactions can enhance crop tolerance to heavy metals, offering a sustainable approach to managing contaminated agroecosystems.^105^

Our triple-goal framework targets key soil management practices to improve microenvironmental conditions and support soil health. Root-derived carbon inputs—such as rhizodeposition, root exudates, and necromass—contribute disproportionately to soil carbon pools compared to aboveground residues. These root inputs are closely linked to microbial activity and enzymatic processes and are considered a critical indicator of soil health.^19^ The abundance of the *cbbL* gene, which encodes bacterial Ribulose-1,5-bisphosphate carboxylase/oxygenase, serves as a proxy for the carbon sequestration potential of soil autotrophic microorganisms.^106^ Higher *cbbL* gene copy numbers are often observed under conservation tillage compared to conventional tillage due to the favorable microenvironments created by added organic matter.^107^ However, responses can vary depending on soil nutrient status and the duration of nutrient management.^108^ In some cases, soil CO_2_-fixing genes may not respond positively to soil disturbance or nutrient amendments.^109^

Another focus of the triple-goal framework is reducing anthropogenic disturbances of the soil structure. Limiting agrochemical inputs such as pesticides and herbicides is critical for preserving microbial diversity and function. Additional practices that promote SOC accumulation and maintain microbial habitats include reducing soil compaction through optimized machinery use, retaining plant residues, and incorporating organic amendments. It is also essential to exclude contaminants, including antibiotics,^110^ microplastics,^111^^,^^112^ and heavy metals^113^—especially when using recycled organic amendments^114^—to protect microbial communities and ensure the delivery of ecosystem services.

Legume-based diversification offers a multifunctional strategy for improving soil health (Figure S3). Incorporating legumes into crop rotations supports BNF and system resilience, reducing the need for synthetic nitrogen inputs and associated CO_2_ emissions. A meta-analysis of 462 studies (11,768 observations) revealed that legume rotations can increase subsequent crop yields by an average of 20% across diverse pedo-climatic conditions.^115^ Regional case studies further demonstrate 7%–13% SOC increases in maize-wheat systems in the Indo-Gangetic Plains^116^ and 50%–102% reductions in CO_2_ emissions in temperate legume-intercropping systems.^117^

Reduced tillage and crop diversification are complementary practices that work together to enhance soil health. A global meta-analysis (comprising 77 articles and 393 treatments) found that combining no-till practices with crop diversification enhances fungal abundance, improves the fungus-bacterium ratio, and optimizes nutrient cycling. However, such practices must be adapted to local conditions, balancing soil health gains with the need to maintain cereal yields, particularly in regions where food security remains a pressing concern.

Innovative cropping strategies can also promote biodiversity-based production systems. For example, perennial tropical crops like banana (*Musa acuminata*) and coffee (*Coffea arabica*), grown in shaded agroforestry systems, support greater biodiversity,^118^ while annual crops such as maize, sugarcane (*Saccharum officinarum* L.), and oil palm (*Elaeis guineensis* L.) tend to diminish when grown in open conditions.^118^ In the northern Great Plains of North America, integrating annual legumes into traditional wheat or oilseed monocultures enhances above- and belowground microbial biodiversity.^119^ In Asia, applying biochar derived from pyrolyzed carbon feedstocks improves soil sustainability.^120^ Selecting appropriate feedstocks and pre-pyrolysis activation methods can enhance biochar’s ability to adsorb and immobilize heavy metals, benefiting the remediation of contaminated environments.^121^

### Conditioning soils with amendments to enhance soil health

The foundational role of soil health in supporting agrifood productivity and resilience is widely acknowledged. However, tailoring amendment strategies to specific soil conditions is crucial for sustaining long-term soil functionality. Within the “triple-goal” framework, a key focus is on enhancing soil health through the use of organic amendments that improve its physicochemical and biological properties. Directly incorporating organic matter—such as livestock manure (raw or processed), green waste compost, and anaerobic digestate—remains a proven approach to increasing SOM.^122^ Emerging circular economy innovations are expanding the range of available amendments, including fishery byproducts repurposed as nutrient-rich fertilizers,^123^ insect frass from black soldier fly farming,^124^ and human-derived fertilizers (e.g., sanitized sewage sludge and urine).^125^ Biochar, a carbon-rich material produced by pyrolysis, also holds promise for conditioning soils by enhancing microbial diversity, stabilizing SOM,^121^^,^ and mitigating soil contamination (e.g., heavy metals) through its strong adsorption capacity.^126^ However, biochar’s effectiveness depends heavily on the type of feedstock and pyrolysis temperature,^121^ and its widespread use is often limited by logistical and financial constraints.^126^ Moreover, biochar addition—alone or combined with other organic amendments—can sometimes increase CO_2_,^127^ N_2_O,^128^ or CH_4_^129^ emissions, raising the overall global warming potential.^130^

Organic fertilizers enhance soil structure by improving aggregation, reducing bulk density, and acting as carbon sinks.^131^ They contribute beneficial microbial consortia while stimulating native microbial communities. However, their effects on soil health are highly variable, influenced by feedstock source, processing technique, and application rate. Significant challenges associated with organic fertilizers include GHG emissions from incomplete mineralization,^132^ the risk of contaminants (e.g., antibiotics and heavy metals),^133^ and issues related to labor intensity and inconsistent nutrient release.^132^

Enhanced rock weathering has recently emerged as a novel strategy for carbon sequestration. This practice involves applying crushed silicate or carbonate minerals (e.g., basalt and dolomite) to agricultural soils. It has the potential to sequester an estimated 0.5–2 billion tons of CO_2_ annually in major cropping regions.^134^ In addition to carbon capture, benefits may include increased soil pH, improved availability of micronutrients (e.g., calcium and magnesium),^135^ and higher crop yields in acidic soils.^136^ However, the long-term effects of rock weathering amendments on soil microbial communities and SOM dynamics remain unresolved.^134^

## Pillar 3: Fewer emissions

### Mitigating N_2_O emissions in the triple-goal framework

Primary food production systems generate 70%–85% of global anthropogenic N_2_O emissions—a potent GHG with a global warming potential of 298 times greater than CO_2_ over a 100-year atmospheric lifetime that exacerbates climate change and stratospheric ozone depletion.^137^ Fertilized croplands are the primary source of N_2_O emissions, driven by microbially mediated nitrification (NH_3_ → NO_2_^−^ → NO_3_^−^) and denitrification (NO_3_^−^ → N_2_O or N_2_), processes regulated by key functional genes (e.g., *nirK*, *nirS*, and *nosZ*). Exogenous N inputs elevate *nirK* and *nirS* abundance while suppressing *nosZ*, skewing nitrogen cycling toward increased N_2_O production. Reducing the oxidation of NH_4_^+^ to NO_3_^−^ is critical, as NO_3_^−^ leaching and subsequent denitrification account for 30%–50% of total nitrogen losses.^103^ In the “triple-goal” framework, emissions are assessed across on-farm (e.g., fertilizer and pesticide application) and off-farm (e.g., production, transport, and storage of agrochemicals) activities (Figure S4). These emissions are expressed as CO_2_ equiv, following Intergovernmental Panel on Climate Change guidelines.^138^ Notably, the production of synthetic nitrogen fertilizer contributes 2.8–16.1 kg CO_2_ equiv per kg of nitrogen applied.^139^

**Figure 6:**
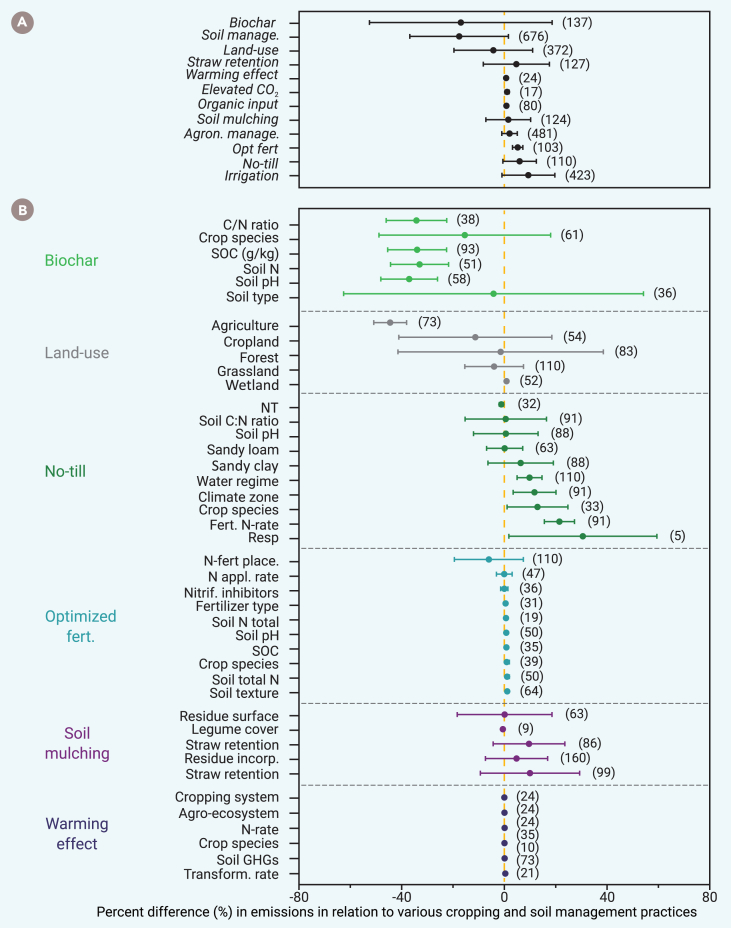
Key drivers regulating N_2_O emissions in agrifood production systems The triple-goal agrifood production system adopts a multidisciplinary approach to simultaneously boost food production, reduce GHG emissions, and improve soil health. This approach system mitigates nitrogen-induced emissions through practices such as (A) biochar application, improved soil management, straw retention, land-use optimization, and reduced tillage during cropping. However, many current anthropogenic activities aimed primarily at increasing crop yields—such as (B) intensive tillage, soil mulching, and climate change-related warming—can exacerbate nitrogen losses.

Anthropogenic interventions play a central role in mitigating N_2_O emissions (Figure 6A). Key strategies include cover cropping and integrating legumes into crop rotations. A meta-analysis of 372 studies found that cover crops can reduce N_2_O emissions by 18%–30% in soils with moderate carbon (∼20 g kg^−1^) and nitrogen (∼3 g kg^−1^) levels.^140^ Legume-based rotations, such as those involving chickpea or pea, can cut emissions by 56–65% compared to canola monocultures,^141^ although tradeoffs exist between maximizing yields and minimizing nitrogen losses (Figure 6B). Tillage and hydrothermal conditions also influence N_2_O emissions, with no tilling increasing emissions by 6%–13% in arid regions but reducing them by 11% in humid^142^ or low-C soils (<20 g kg^−1^).^143^ However, these effects tend to diminish over time as the soil structure improves and reduces anaerobic microsites.^144^ A meta-analysis of 37 studies found no consistent impact of tillage (up to 40 years) on N_2_O emissions, regardless of the tillage method used (e.g., moldboard plow, chisel plow, or double-disk systems).^145^

Crop plants typically absorb only a portion of applied nitrogen fertilizer, with the remainder, particularly mobile NO_3_, becoming a substrate for nitrification and denitrification.^103^ Globally, NUE—the proportion of fertilizer nitrogen taken up by plants—ranges from 25% to 50% in season, with an additional 5%–20% uptake in the subsequent season.^146^ Precision nitrogen management strategies, such as 4R,^19^ can significantly enhance NUE using slow-release fertilizers, nitrification inhibitors, and variable-rate application.

Structural equation modeling identifies N_2_O as the dominant nitrogen loss pathway, decoupled from runoff and respiration losses (Figure S5). Among mitigation strategies, optimizing BNF offers significant potential (Figure S6). Currently, BNF contributes 1.4 Mt of nitrogen annually, a figure projected to increase by 56% by 2100 under elevated CO_2_ conditions.^147^ Key strategies to enhance BNF include (1) legume and non-legume intercropping (e.g., *Arachis hypogaea*-maize rotation), which promotes rhizosphere metabolites (flavonoids and coumarins), nodulation, and nitrogen fixation^148^; (2) microbial synergies, such as co-inoculation with AM fungi and *Rhizobium*, which improves phosphorus uptake^57^ and alleviates nitrogen limitations^149^; and (3) adaptation to soil-climate interactions, as optimal BNF occurs at ∼25°C. Climate change is expected to enhance BNF at higher latitudes (+50%) while reducing efficacy in tropical regions (−50%).^150^ Non-symbiotic BNF (e.g., in crops like sugarcane and tobacco) play a minimal role in global nitrogen inputs due to low carbon use efficiency (0.012–0.02 g N per g C).^151^

### Exploring CO_2_ fertilization by promoting CO_2_ biotransformation

Over the past 50 years, the seasonal amplitude of atmospheric CO_2_ has increased in the Northern Hemisphere,^152^^,^^153^ intensifying a “fertilization effect” that enhances photosynthesis and promotes the conversion of CO_2_ into plant biomass, which will likely significantly increase carbon capture via vegetation in the coming decades. Within the triple-goal framework, a key focus is on optimizing this biotransformation by leveraging source-sink mechanisms, including leaf area index, canopy architecture, aboveground biomass accumulation, the evaporation-to-transpiration ratio, solar energy interception, and plant respiration. These physiological and structural dynamics could mitigate up to 30% of anthropogenic CO_2_ equiv emissions. However, the terrestrial hydrological cycle influences the extent of the CO_2_ fertilization effect, impacting the rate of CO_2_ conversion. Additionally, enhancing CO_2_ biotransformation via the CO_2_ fertilization effect could increase soil carbon emissions^153^ due to shifts in land-atmosphere carbon fluxes, which are governed mainly by atmospheric CO_2_ levels and temperature.

The triple-goal framework proposes several management strategies to enhance carbon source-to-sink biotransformation under rising CO_2_ levels: “precision agriculture,”^154^— using data-driven technologies like geographic information systems,^155^ remote sensing,^156^ and the Internet of Things^157^ to optimize resource use, monitor crop performance, and increase productivity with reduced environmental impact, and “vertical farming,” incorporating hydroponics, aeroponics, and Light-Emitting Diode lighting in vertically stacked systems^158^ to maximize space efficiency and enable continuous urban food production. However, its contribution to global food supply remains limited; “climate-smart agriculture” (CSA)—promotes CSA approaches to support sustainable agrifood systems by improving soil health, crop yields, and resilience to biotic and abiotic stresses while simultaneously reducing GHG emissions.^159^ Scaling up CSA requires ongoing investment in capacity building, communication, and farmer engagement. It is key to understanding growers’ perceptions and willingness to adopt adaptation and mitigation strategies. Cross-sectoral collaboration is also vital for long-term success. “Automated systems”—integrating technologies such as smart sensors, drones, robotic harvesters, and automated irrigation^160^^,^^161^ to improve operational efficiency and environmental sustainability across indoor and open-field agriculture; “technological innovation” leverages cutting-edge tools like gene editing (e.g., CRISPR-Cas9^162^) to develop cultivars with improved resource use efficiency, high-throughput sequencing to study soil-root-microbe‒environment interactions,^163^ remote sensing technologies (Normalized Difference Vegetation Index and Enhanced Vegetation Index) to more precisely map carbon sinks and water availability,^164^ and explore biofertilizer options,^165^ such as AM fungal inoculants for promoting sustainable development.^166^ Introducing N_2_O-reducing bacteria in the hyphosphere offers further potential for reducing emissions.^167^ Additionally, digital cropping systems can enhance sustainability,^168^^,^^169^ and the root-associated microbiota plays a vital role in conferring plant resistance to abiotic stresses such as aluminum toxicity and phosphorus deficiency.^105^ Despite these advancements, significant disparities in access to innovation persist between wealthy and low-income countries. Addressing this inequity will require broader societal and cultural transformation to ensure that the benefits of advanced emerging technologies are shared globally.

## Integration for local solutions

**Figure 7:**
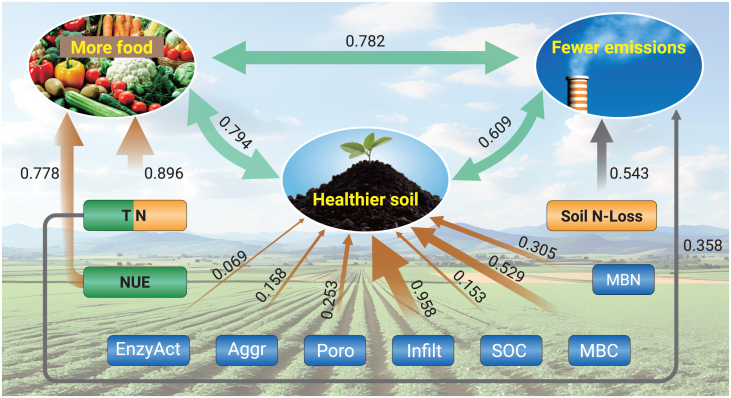
The relationship between food production, soil health, and GHG emissions The three components—food production, soil health, and GHG emissions—are strongly interconnected, with correlation coefficients greater than 0.609. Total soil nitrogen (TN) is the most influential factor in achieving high food production, followed by nitrogen use efficiency (NUE); both are positively associated with food production and emissions. Key soil health indicators include infiltration (Infil), MBC, MBN, and porosity (Poro). Soil enzyme activity (EnzAct), aggregate stability (Aggr), and SOC also contribute positively, though to a lesser extent. Some factors with minimal relevance to the triple-goal framework were excluded. Correlation coefficients are shown alongside the connecting lines.

The triple-goal framework integrates novel and improved strategies to optimize the balance between the three pillars: agrifood production, soil health, and GHG emissions (Figure 7). A synthesis of more than 39,000 studies revealed a highly significant positive correlation among the three pillars, with coefficients exceeding 0.61, indicating that technologies aimed at boosting agrifood yields often result in increased GHG emissions. High crop yields depend primarily on total nitrogen and NUE as key determinants. Notably, total soil N plays a dual role—showing a strong positive correlation with food production (*r* = 0.90) and a moderate correlation with emissions (*r* = 0.36). The primary sources of GHG emissions are soil nitrogen losses and changes in total soil nitrogen levels. Soil infiltration, MBC, MBN, porosity, aggregate stability, and enzyme activity are all key contributors to soil health. The observed positive relationship between food production and emissions primarily stems from the addition of fresh carbon to the system through increased inputs of fertilizer and organic amendments. Our SOMA identified the relative influence of over 120 natural and anthropogenic factors affecting these interconnections. These factors are associated with soil physical properties, agronomic practices, and environmental conditions. Effective nitrogen management is crucial for balancing the nexus of food production, soil health, and GHG emissions. However, not all solutions are universally applicable. Some practices that support soil biological functions may hinder other processes. For example, conservation agriculture, residue retention, and no-till methods can help retain SOM in drier regions but may increase soil-borne pathogens in humid regions.^170^

There is no one-size-fits-all solution for enhancing soil health, increasing food production, and reducing emissions across diverse cropping systems. Strategies must be tailored to local contexts. While crop diversification has many benefits, its success depends on regional markets, farm size, farmer incentives, and social and cultural practices. Currently, there is no globally accepted framework for carbon farming, and although agricultural soils have significant potential for climate mitigation, the mechanisms underlying this potential remain poorly understood. Developing cost-effective monitoring tools and performance indicators will help farmers and land managers implement practical, locally adapted strategies aligned with the triple-goal approach. Institutional and government support will enable flexible management that takes into account current social, climatic, and environmental conditions. Coordinated policies and guidelines are necessary across all governance levels to drive progress toward these three interconnected goals. The “triple-goal” framework promotes the integration of innovative and proven practices to develop localized solutions that address the tradeoffs among food production, soil health, and environmental outcomes.

## Perspective for future growth

Since the Green Revolution, countries have followed diverse development trajectories in building effective agrifood systems. However, the path toward resilience is highly context specific and must be tailored to each nation’s unique biophysical and socioeconomic conditions.^171^ Climate change is projected to have profound effects on agriculture, underscoring the urgency of adopting climate-resilient food production systems that address both synergies and tradeoffs in mitigation and adaptation.^172^ There is a growing need for innovative approaches that prioritize productivity and sustainability to meet global food demands while minimizing environmental degradation.^173^ Emerging digital technologies—like sensors, uncrewed aerial vehicles, GPS-guided systems, autonomous monitoring devices, and advanced data analytics—offer promising solutions for improving productivity^174^ and resource use efficiency while reducing GHG emissions. Artificial intelligence is revolutionizing agriculture by enabling precise, data-driven input applications that optimize crop growth and reduce waste. However, to fully realize the potential of these technologies, challenges such as high initial investment costs, disparities in technology access, data privacy concerns, and the need for farmer education must be addressed.

Agrifood systems today must strike a delicate balance: feeding a growing global population, adapting to climate change, and mitigating GHG emissions while simultaneously restoring degraded soils. Despite soil health not being included in the United Nation’s (UN’s) Sustainable Development Goals,^175^ it has gained prominence through the “One Health” initiative,^176^ which seeks to optimize the health of people, animals, and ecosystems.^177^ Current research increasingly emphasizes climate adaptation and crop sensitivity,^178^ and the frameworks introduced by the World Bank and FAO under the International Assessment of Agricultural Knowledge, Science, and Technology for Development 2002 offer practical strategies to support these goals. We advocate for a renewed emphasis on conversation agriculture, tailored across biophysical and socioeconomic contexts. This paper’s triple-goal agrifood production framework integrates emerging and established technologies to enhance productivity, mitigate N_2_O-driven GHG emissions, and promote holistic water-carbon-nutrient cycling, thereby improving soil health. This approach provides a roadmap for decision-makers to develop climate-smart, resource-efficient, and stress-resilient agrifood systems, serving as a potential global model for multifunctional agriculture.

Nonetheless, we acknowledge potential limitations in implementing the triple-goal system. In less developed countries, for example, economic constraints may favor cash cropping over diversified systems, limiting the feasibility of cropping diversification. Regional agricultural conditions may also restrict the adoption of a diverse crop mix, depending on climate, soil type, and market access. Effective policy development requires collaboration among governments, business sectors, and local communities to address barriers to adopting practices such as no-till farming and crop diversification. Strategies that have proven successful in more developed contexts may require adaptation and supportive infrastructure in less developed regions. In areas where local economies rely on cash crops, producing cereals may not be a viable alternative. Integrating livestock into cropping systems—such as crop-tree-livestock configurations—can enhance nutrient cycling and support the production of diverse food products for human and animal consumption.

## Conclusion

Global agrifood systems are significant contributors to GHG emissions, intensifying the impacts of climate change, while growing food demands exacerbate these challenges. At the same time, soil health has deteriorated significantly in recent decades, creating a self-reinforcing cycle of degradation from field to fork. Traditional agrifood systems have largely failed to eradicate hunger and malnutrition or to reverse worrying trends such as soil degradation, water pollution, and biodiversity loss. A sustainable agrifood production system must deliver food security while actively restoring soil health to help achieve a future that is land degradation neutral. Our proposed triple-goal framework seeks to enhance resource use efficiencies (including water, fertilizers, and economic investment), improve soil and land quality, and increase food production from less land while boosting soil carbon sequestration. This integrated approach offers a pathway to contribute meaningfully to the UN’s Sustainable Development Goals by promoting soil health and strengthening resilience to climate change. Broad adoption of the triple-goal framework could provide a strategic roadmap for transforming agrifood production globally, ensuring healthy soils, affordable and nutritious food, and reduced environmental harm. While this system alone will not eliminate global hunger, it sets the foundation for a more productive, sustainable, and resilient future for agrifood systems worldwide.

## Funding and Acknowledgments

The views expressed in this paper are those of the authors and should not be construed as reflecting the official position of their respective affiliations. The project was supported by the 10.13039/501100001809National Natural Science Foundation of China (32472826), the Leading Project of the “Three Agri-Priorities with Nine Directions” Science and Technology Collaboration Plans in Zhejiang Province (2025SNJF016), the Wenzhou University research start-up fund (QD2024084), and the Wenzhou City Talent Introduction fund (R20241101). The funders had no role in the study design, data collection and analysis, decision to publish, or the preparation of the manuscript.

## Author contributions

L. Wang,^1^^,^^2^^,^^3^ G.Y.G., and M.Z., conceptualized the review. G.M.G., G.Y.G., E.A.K., and D.P.-B. contributed section materials. L.C.S. and K.F.D. brought out the critical issues relative to the subject, reviewed the draft and revisions, and provided novel ideas to improve the work. K.H.M.S. reviewed and rewrote subsections and edited versions. T.G., S.G., S.F., M.H., C.H., L. Wang,^1^^,^^6^^,^^7^ and S.-J.L. contributed subsection materials to the paper. H.C., Z. Wei, J.X., and Z. Wang data collection, meta-analysis, and graphics. All authors contributed to the manuscript, agreed on the contents and authorship, and approved the final version. G.Y.G. and L. Wang^1^^,^^2^^,^^3^ finalized the manuscript for publication.

## Declaration of interests

The authors declare no competing interests.

## Supplemental information

Document S1. Figures S1–S7 and Tables S1–S3

Document S2. Article plus supplemental information
