# Supplementary material for: Integrated strategies for enhancing agrifood productivity, lowering greenhouse gas emissions, and improving soil health
Source: Innovation (Camb). 2025 Jun 25;6(11):101006. doi: 10.1016/j.xinn.2025.101006 (PMC12628179; doi:10.1016/j.xinn.2025.101006)
Supplement: Document S1. Figures S1–S7 and Tables S1–S3 [file mmc1.pdf]

The Innovation, Volume *6*

Supplemental Information

Integrated strategies for enhancing agrifood productivity, lowering greenhouse gas emissions, and improving soil health

Li Wang, Gina Marie Garland, Tida Ge, Shiqian Guo, Endalkachew Abebe Kebede, Chengang He, Mohamed Hijri, Daniel Plaza-Bonilla, Lindsay C. Stringer, Kyle Frankel Davis, Soon-Jae Lee, Shoujiang Feng, Li Wang, Zhenyang Wei, Hanwen Cao, Zhi Wang, Jiexiong Xu, Kadambot H.M. Siddique, Gary Y. Gan, and Min Zhao

Supplementary file

TOWARD 'TRIPLE-GOAL' AGRIFOOD SYSTEMS

Li Wang[1,2], Gina Marie Garland[3,4], Junling Zhang[5], Tida Ge[6], Mohamed Hijri[7], Chengang He[8], Daniel Plaza-Bonilla[9], Lindsay C. Stringer[10], Endalkachew Abebe Kebede[11], Kyle Frankel Davis[11,12], Soon-Jae Lee[13], Shoujiang Feng[2], Zhenyang Wei[2], Hanwen Cao[2], Zhi Wang[2], Jiexiong Xu[2], Kadambot H.M Siddique[14], Fusuo Zhang[5*], Gary Y. Gan[2,15*], Min Zhao[2*]

Includes: More descriptions on Methods used in the study

Table S1, Table S2, Table S3

Figure S1

Figure S2

Figure S3

Figure S4

Figure S5

Figure S6

Figure S7

More descriptions on Methods

Rationale of Using A 'Second-Order Meta-Analysis' (Abbreviation SOMA)

Numerous studies have investigated the impact of crop- and soil-related anthropogenic activities on agrifood production, soil health, and GHG emissions, and the results have been documented in various scientific literatures through a rapidly growing number of first-order meta-analyses. However, most of the first-order meta-analyses synthesize the findings on individual issues, lacking a comprehensive understanding of multi-factor effects and their interactions. A close examination of individual first-order meta-analysis reveals that their results are highly variable, inconsistent, and sometimes contradictory. This was due to large variations in their research scopes, scales, experimental structure (e.g., treatment complex versus simplicity), and the number of experiments included in the original meta-analyses. Also, original studies were conducted under various soil-climatic conditions across different geographical regions, and the results differed substantially among the studies that were included in the first-order meta-analysis[1].

In contrast, a large review can be time-consuming and expensive, but it has a better chance of identifying underlying patterns of variability that may be of use to the field. A SOMA is less costly and less time-consuming while providing sufficient power of identifying novel findings, as a SOMA is designed to synthesize the results of multiple first-order meta-analyses in a quantitative and comprehensive way. A SOMA differs from a typical first-order meta-analysis which synthesizes the results of systematic review of published articles on a specific subject. Also, a SOMA differs from a conventional literature review which typically synthesizes available evidence on a certain topic utilizing pre-specified eligibility criteria for including articles with a systematic method for its production. A SOMA provides a robust way to deal with the heterogeneities across the various studies, enabling to foster the impact of soil- and crop-related anthropogenic activities on agrifood system productivity, GHG emissions, and soil health. Moreover, a SOMA approach represents an economical means of providing an answer to big questions in research determinations[2]. The strongest point in a SOMA is its ability to provide evidence to answer a general question by taking a substantive body of hard data into

consideration. The synthesis with the validation process of a SOMA indicates that the approach is an adequate technique for synthesizing effect sizes and estimating the average effect size in relation to a specific phenomenon. A SOMA allows for using moderator analysis to answer more specific questions pertaining to various study features of interest. By applying the standard procedures of systematic reviews to the synthesis of meta-analyses, the SOMA is intended to capture the essence of the existing body of literature on the subject. Additionally, the SOMA approach may prove to be helpful when reliable answers to global questions are required within limited time frames and with limited resources.

Article selection criteria and logistic steps

In the SOMA, we took the following logistic steps:

First, we defined and listed the subject areas to be discussed in our article, aiming at finding how anthropogenic activities relative to crop and soil management practices impact the triple-goal system.

Second, we identified original meta-analysis articles closely relevant to the defined subject areas through searching for the most popular academic search engines Web of Science-AHCI, Web of Science-SCIE, Web of Science-SSCI, Elsevier ScienceDirect, and Google Scholar. As a result, 190 out of 4712 meta-analyses in the subject area of interest were identified that might meet the study objectives (**Figure S1**).

Third, we preset article selection criteria: an article had (1) analyzed the effect of one or several factors on at least two of the three factors (food, soil, emissions), (2) presented a statistical analysis of at least two primary studies on at least two of the three factors, (3) reported indicators of precision of the effect sizes (standard errors, standard deviation, or confidence intervals), and (4) provided details on the methods used in the original studies which can be found in the paper or supplementary files.

Fourth, we entered the basic information presented in each of the 190 original meta-analyses into a spreadsheet (final version to be disclosed via figshare at https://figshare.com/) and then we extracted relevant data on emissions, soil health, and

crop yield or system productivity from the individual articles to generate a Master spreadsheet. A careful examination of the 190 articles during and after the data extraction ensured that each meta-analysis contained at least two of the three factors (food, soil, emissions) in the same article.

Removing overlayed articles

The same article may have been selected by different original meta-analyses, which may have some sort of 'cumulative effects' with time and space overlayed. For instance, an article describing the effect of AM fungi on soil health may have been selectively used by multiple (say six) original meta-analyses. If all those six meta-analyses were to be included in our SOMA, it would result in a 'cumulative effect' of the AM fungi effects. In this case, the requirement of statistical independence to test the classes of the moderator may be a concern. To avoid this sort of pseudo-replication, we examined all the references in the identified original meta-analyses and calculated the proportion of shared original studies among the meta-analyses, and we found that overlayed articles in the different original meta-analyses were fewer than 20%, thus, the 'cumulative effects' with time and space is at a low and acceptable level based on the findings by Florence et al[3]. This process strikes a balance between reducing significant dependence, but maintained important large studies. So, a smaller than 25% amounts of overlap is considered reasonable[4]. Thus, we included only original meta-analyses with a maximum of 25% of shared original studies in the SOMA. This cutoff was based on (1) the studies focusing only on the three pillars of interest in our study, (2) the list of studies was included in their original first-order meta-analyses, and (3) the effect of anthropogenic activities (soil and crop management-related practices) on the response variables was summarized. As a result, the search yielded **104** original meta-analyses that fit our preset selection criteria (**Table S1**).

Data extraction & management

For each of the 104 selected articles, we extracted data from the control and the response groups. All the effect sizes of the original meta-analyses were then extracted from the text, tables or figures. We used WebPlotDigitizer (https://automeris. io/WebPlotDigitizer/)

to extract data from figures of original meta-analyses. The list of primary studies (and their DOIs, when available) used in each meta-analysis was retrieved, allowing us to identify the number of common primary studies between each pair of meta-analyses when needed. Finally, we characterized the meta-analyses included in our study by pre-set criteria related to the literature search and potential bias analysis. We entered the basic information of each selected article into a spreadsheet (https://figshare.com). A total of 39,162 studies or field experiments with over 300,000 observations were recorded in the 104 meta-analyses (**Table S2**), and the studies were conducted across the globe (Global meta-analyses) or in specific regions (Regional meta-analyses).

Effect size

Effect Sizes: We extracted the ratio, percentage change, possible transformations, confidence intervals or other indicators of variability, and the number of primary studies and observations that were used to calculate the effect sizes. Also, we extracted and collected the pooled effect sizes (e.g., mean differences, odds ratios) and their variances (standard errors, confidence intervals) from each first-order meta-analysis.

In the SOMA, we use a random-effects model of the Comprehensive Meta Analysis (CMA)—one of the best meta-analysis tools at the present time. The CMA's build-in features can handle two levels of effect sizes: the observed effect sizes (Hedges' g) from each first-order meta-analysis, and the distribution of these effects across meta-analyses. This is based on the assumptions that each first-order meta-analysis provided an unbiased estimate, and that the variances were known or estimated. Also, heterogeneity measures like Tau^2 and I^2 are built to quantify between-study variance. The SOMA combines these, considering both the variance within each meta-analysis and the variance between them.

Standardization: To ensure consistency in the SOMA metrics, we standardized mean differences by converting all the effect size to **Hedges' g** (a bias-corrected standardized mean difference).

Cross-Validation

A cross validation offers support for the accuracy of the effect size synthesis and the SOMA results. The extensive literature search and a systematic review process resulted in the inclusion of 104 meta-analyses with overlap below 25% in the 39,000+ primary studies, resulting in an overall effect size of **0.67** under the random effects model, which is significantly different from zero. To validate our SOMA, we extracted 216 individual, independent effect sizes from 87 out of 104 articles which reported effect size or ratios, leading to a mean effect size of **0.48** under the random effects model. The average effect size **0.67** in our analysis is substantially greater than 0.48 in the 'sampled' meta-analyses. Effect size is a quantitative measure of the magnitude of the experimental effect. The larger the effect size the stronger the relationship between an experimental group and the control group for the target variable. When the sample size increases, the power for detecting a given effect size increase. In other words, increasing sample size while holding statistical power constant at a particular level (e.g., 95%) allows to detect a smaller effect size at that level or the chance of detecting a given effect size with a given power, where increased precision is what allowed us to detect a smaller effect with a larger sample.

Publication bias

An important issue with meta-analyses is publication bias that indicates an association between the publication status of a manuscript and the magnitude of the effect found. In our analysis, potential publication bias was assessed with funnel plots (**Figure S7**). Studies with high precision will be plotted near the average mean effect, and studies with low precision will be spread on both sides. To minimize publication bias, we took the following approaches:

1) Included meta-analyses describing experiments conducted in a field setting, and excluded meta-analyses of laboratory experiments, case studies, survey data, and qualitative studies;
2) Included meta-analyses that incorporated studies with a control group or a proxy for a control group, that incorporated both within- and between-subject designs and multiple measurement points, and that provided (either in the article, through open

access sources, or in correspondence with the authors) adequate statistics for us to calculate both an effect size measure and a measure of dispersion. These measures helped minimized potential publication bias as the funnel plot showed only weak indications of asymmetry;

3) Excluded low-quality studies or studies with insufficient information to calculate effect sizes or SEs;
4) Assessed publication bias for all accessible effect sizes reported in the original meta-analyses; and
5) Tested the sensitivity of the results against publication bias using Rosenthal fail-safe number, i.e., the number of additional studies with a mean null result necessary to provide a non-significant global estimated effect. The CMA model we used provide Frequentist model parameters estimated by maximum likelihood; this gives some sense of the robustness of our model results.

Limitations

Our search resulted in 104 first-order meta-analyses that were included in the present study. We first searched articles with "meta-analysis" in the article title. The rationale was that the researchers would include the term "meta-analysis" or equivalent in the title along with the descriptors. Then we searched the keywords targeting the Food – Soil – Emission metrics. An article must present at least two of the three goals in the same article, assuring our moderator analyses include sufficient studies in each goal. If the original meta-analysis did not report moderator analyses, we could not report it in the SOMA. We averaged effect sizes when multiple effect sizes were reported for a single outcome, ignoring any within-subject variability[4].

We used CMA random-effect models, given the relatively large number of studies (104) for moderator analyses[5]. Relatedly, readers must always be cautious in interpreting findings from a meta-analytic study since the analysis builds off existing published studies, which have their own set of limitations. Our study did not fully examine or discuss the specific practices of original first-order researchers. This could also have explanatory power for the effect sizes. Also, our operationalization of quality does not

consider all possible study components, and we did not verify beyond what the authors reported.

References

1 Gurevitch, J., Koricheva, J., Nakagawa, S. & Stewart, G. Meta-analysis and the science of research synthesis. *Nature* **555**, 175-182 (2018). https://doi.org/10.1038/nature25753

2 Tamim, R. M., Bernard, R. M., Borokhovski, E., Abrami, P. C. & Schmid, R. F. What forty years of research says about the impact of technology on learning: A second-order meta-analysis and validation study. *Review of Educational Research* **81**, 4-28 (2011).

3 Martin, F., Sun, T., Westine, C. D. & Ritzhaupt, A. D. Examining research on the impact of distance and online learning: A second-order meta-analysis study. *Educational Research Review* **36**, 100438 (2022). https://doi.org/https://doi.org/10.1016/j.edurev.2022.100438

4 Polanin, J., Maynard, B. & Dell, N. A. Overviews in education research: A systematic review and analysis. *Review of Educational Research* **87** (2016). https://doi.org/10.3102/0034654316631117

5 Borenstein, M. in *Systematic Reviews in Health Research. https://doi.org/10.1002/9781119099369.ch27* 535-548 (2022).

6 Lupwayi, N. Z., Larney, F. J., Blackshaw, R. E., Kanashiro, D. A. & Pearson, D. C. Phospholipid fatty acid biomarkers show positive soil microbial community responses to conservation soil management of irrigated crop rotations. *Soil Tillage Res.* **168**, 1-10 (2017). https://doi.org/https://doi.org/10.1016/j.still.2016.12.003

7 Schmidt, R., Gravuer, K., Bossange, A. V., Mitchell, J. & Scow, K. Long-term use of cover crops and no-till shift soil microbial community life strategies in agricultural soil. *PLoS One* **13**, e0192953 (2018). https://doi.org/10.1371/journal.pone.0192953

8 Wang, Y. *et al.* Long-term no-tillage and organic input management enhanced the diversity and stability of soil microbial community. *Sci. Total Environ.* **609**, 341-347 (2017). https://doi.org/https://doi.org/10.1016/j.scitotenv.2017.07.053

9 Chuntao Yin, N. M., Scot Hulbert, Daniel Schlatter, Timothy C. Paulitz, Kurtis Schroeder, Aaron Prescott, Amit Dhingra. Bacterial Communities on Wheat Grown Under Long-Term Conventional Tillage and No-Till in the Pacific Northwest of the United States. *Phytobiomes Journal* **1**, 83-90 (2017). https://doi.org/10.1094/pbiomes-09-16-0008-r

10 Wyngaard, N., Franklin, D. H., Habteselassie, M. Y., Mundepi, A. & Cabrera, M. L. Legacy Effect of Fertilization and Tillage Systems on Nitrogen Mineralization and Microbial Communities. *Soil Sci. Soc. Am. J.* **80**, 1262-1271 (2016). https://doi.org/10.2136/sssaj2016.03.0070

11 Somenahally, A. *et al.* Microbial communities in soil profile are more responsive to legacy effects of wheat-cover crop rotations than tillage systems. *Soil Biol. Biochem.* **123**, 126-135 (2018). https://doi.org/https://doi.org/10.1016/j.soilbio.2018.04.025

12 Tyler, H. L. Bacterial community composition under long-term reduced tillage and no till management. *J. Appl. Microbiol.* **126**, 1797-1807 (2019). https://doi.org/10.1111/jam.14267

13 Laudicina, V. A., Novara, A., Barbera, V., Egli, M. & Badalucco, L. Long-Term Tillage and Cropping System Effects on Chemical and Biochemical Characteristics of Soil Organic Matter in a Mediterranean Semiarid Environment. *Land Degrad. Dev.* **26**, 45-53 (2015). https://doi.org/10.1002/ldr.2293

14 Babin, D. *et al.* Impact of long-term agricultural management practices on soil prokaryotic communities. *Soil Biol. Biochem.* **129**, 17-28 (2019). https://doi.org/10.1016/j.soilbio.2018.11.002

15 Sommermann, L. *et al.* Fungal community profiles in agricultural soils of a long-term field trial under different tillage, fertilization and crop rotation conditions analyzed by high-throughput ITS-amplicon sequencing. *PLoS ONE* **13**, e0195345 (2018). https://doi.org/10.1371/journal.pone.0195345

16 Le Guillou, C. *et al.* Tillage intensity and pasture in rotation effectively shape soil microbial communities at a landscape scale. *MicrobiologyOpen* **8**, e00676 (2019). https://doi.org/10.1002/mbo3.676

17 Henneron, L. *et al.* Fourteen years of evidence for positive effects of conservation agriculture and organic farming on soil life. *Agron. Sust. Dev.* **35**, 169-181 (2015). https://doi.org/10.1007/s13593-014-0215-8

18 Yangjin, D., Wu, X., Bai, H. & Gu, J. A meta-analysis of management practices for simultaneously mitigating N2O and NO emissions from agricultural soils. *Soil Tillage Res.* **213**, 105142 (2021). https://doi.org/https://doi.org/10.1016/j.still.2021.105142

19 Shakoor, A. *et al.* A global meta-analysis of greenhouse gases emission and crop yield under no-tillage as compared to conventional tillage. *Sci. Total Environ.* **750** (2021). https://doi.org/10.1016/j.scitotenv.2020.142299

20 Li, Y. *et al.* The role of conservation agriculture practices in mitigating N2O emissions: A meta-analysis. *Agron. Sust. Dev.* **43**, 63 (2023). https://doi.org/10.1007/s13593-023-00911-x

21 Ruis, S. J., Blanco-Canqui, H., Jasa, P. J. & Jin, V. L. No-till farming and greenhouse gas fluxes: Insights from literature and experimental data. *Soil Tillage Res.* **220** (2022). https://doi.org/10.1016/j.still.2022.105359

Table S1. Basic information on the 104 meta-analysis articles, including number of studies and total observations reported in the published articles.

Code	Reference	Article title	Publication year	# of studies (expt.)	Total obs.	Number of test sites	Effect size estimate model	Geographic area	Main treatment	Main effect	[1] Dropdown for all the references used in the original meta-analysis articles
1	Sun et al. 2021	Elevated CO2 shifts soil microbial communities from K- to r-strategists	2021/03/12	122	965		Random	Globe	Elevated CO2	shift soil microbial communities from K- to r-strategists	
2	Rocci et al. 2021	Soil organic carbon response to global environmental change depends on its distribution between mineral-associated and particulate organic matter: A meta-analysis	2021/06/22	168	216		Random	Globe	soil distribution between mineral-associated and particulate organic matter	Soil organic carbon response to global environmental change	Allard V, Newton PCD, Lieffering M, Soussana JF, Carran RA, Matthew C (2005) Increased quantity
3	You et al. 2022	Global meta-analysis of terrestrial nitrous oxide emissions and associated functional genes under nitrogen addition	2022/2/*	144	2068		Random	Globe	nitrogen addition	terrestrial nitrous oxide emissions and associated functional genes	Long-term fertilization in the activity and community structure of ammonia oxidizers3
4	Nunes et al. 2020	Biological soil health indicators respond to tillage intensity: A US meta-analysis	2020/06/15	302			Random	US	Biological soil health indicators	tillage intensity	
5	Mondal et al. 2020	A global analysis of the impact of zero-tillage on soil physical condition, organic carbon content, and plant root response	2019/11/07	522	4131		Random	Globe	zero-tillage	soil physical condition, organic carbon content, and plant root response	Supplementary Information
6	Zheng et al. 2019	Irrigation leads to greater maize yield at higher water productivity and lower environmental costs: a global meta-analysis	2019/03/07	162	1490	21	Random	Globe	Irrigation	greater maize yield at higher water productivity and lower environmental costs	Database used for analysis yield and WP in this study collected from peer-reviewed literatures from 1970 to 2018
7	Huang et al. 2018	Greenhouse gas emissions and crop yield in no-tillage systems: A meta-analysis	2019/3/*	90	740		Random	Globe	no-tillage systems	Greenhouse gas emissions and crop yield	Ahmad, S., Li, C., Dai, G., Zhan, M., Wang, J., Pan, S., Cao, C., 2009. Greenhouse gas emission
8	Dai et al. 2018	Long-term nitrogen fertilization decreases bacterial diversity and favors the growth of Actinobacteria and Proteobacteria in agro-ecosystems across the globe	2018/04/12	70	427		Random	Globe	Long-term nitrogen fertilization	decreases bacterial diversity and favors the growth of Actinobacteria and Proteobacteria in agro-ecosystems across the globe	
9	Wang et al. 2016	Denitrification in upland of China: Magnitude and influencing factors	2016/12/09	39	300		Random	China	Denitrification in upland of China	Magnitude and influencing factors	

10	Tan et al. 2023	Influence of arbuscular mycorrhizal fungi on bioaccumulation and bioavailability of As and Cd: A meta-analysis	2023/1/*	194	1430		Random	Globe	rbuscular mycorrhizal fungi	bioaccumulation and bioavailability of As and Cd	
11	Beillouin et al. 2023	A global meta-analysis of soil organic carbon in the Anthropocene	2023/06/22	25000	190200		Random	Globe		soil organic carbon	Data availability
12	Morugán-Coronado et al. 2022	The impact of crop diversification, tillage and fertilization type on soil total microbial, fungal and bacterial abundance: A worldwide meta-analysis of agricultural sites	2022/01/22	393			Random	Globe	crop diversification, tillage and fertilization type	soil total microbial, fungal and bacterial abundance	References*
13	Muhammad et al. 2021	Cover cropping enhances soil microbial biomass and affects microbial community structure: A meta-analysis	2021/1/*	81	1824	81	Random	Globe	Cover cropping	enhances soil microbial biomass and affects microbial community structure	Reference list for the meta-analysis
14	Curtright et al. 2021	Intercropping increases soil extracellular enzyme activity: A meta-analysis	2021/05/27	100	969		Random	Globe	Intercropping	increases soil extracellular enzyme activity	Appendix: Studies included in meta-analysis
15	Xiao et al. 2018	A meta-analysis of soil extracellular enzyme activities in response to global change	2018/8/*	132	1577	133	Random	Globe	soil extracellular enzyme activities	global change	title
16	Chagas et al. 2022	Biochar increases soil carbon pools: Evidence from a global meta-analysis	2022/3/*	169	586	184	Random	Globe	Biochar	increase soil carbon pools	
17	Wang et al. 2021	Differential effects of altered precipitation regimes on soil carbon cycles in arid versus humid terrestrial ecosystems	2021/9/*	214	845		Random	Globe	altered precipitation regimes	soil carbon cycles in arid versus humid terrestrial ecosystems	
18	Jiang et al. 2017	Higher yields and lower methane emissions with new rice cultivars	2017/05/04	18	93	21	Random	Globe	new rice cultivars	Higher yields and lower methane emissions	
19	Luo et al. 2018	Organic amendments increase crop yields by improving microbe-mediated soil functioning of agroecosystems: A meta-analysis	2018/9/*	106	690		Random	Globe	Organic amendments	improving microbe-mediated soil functioning of agroecosystem, increase crop yields	Supplementary for:1
20	Miao et al. 2019	Soil extracellular enzyme activities under long-term fertilization management in the croplands of China: a meta-analysis	2019/04/04	85			Random	China	Soil extracellular enzyme activities	long-term fertilization management in the croplands	List of references used for meta-analysis
21	Zhao et al. 2017	Roles of nitrogen, phosphorus, and potassium fertilizers in carbon sequestration in a Chinese agricultural ecosystem	2017/04/29	84	385		Random	China	nitrogen, phosphorus, and potassium fertilizers	carbon sequestration in an agricultural ecosystem	Reference

22	Zhao et al. 2019	Sustaining crop production in China's cropland by crop residue retention: A meta-analysis	2019/11/*	278	4910		Random	China	crop residue retention	Sustaining crop production in China's cropland	
23	Zhou et al. 2016	Similar responses of soil carbon storage to drought and irrigation in terrestrial ecosystems but with contrasting mechanisms: A meta-analysis	2016/07/15	195			Random	Globe	drought and irrigation	soil carbon storage, contrasting mechanisms	Text S1 A list of 179 papers from which the data were extracted for this meta-analysis
24	Gao et al. 2022	Warming-induced greenhouse gas fluxes from global croplands modified by agricultural practices: A meta-analysis	2022/5/*	104	449		Random	Globe	agricultural practices, warming	greenhouse gas fluxes	Supplementary Information
25	Han et al. 2021	Global soil organic carbon changes and economic revenues with biochar application	2021/11/*	70	389		Random	Globe	biochar application	organic carbon changes and economic revenues	
26	Li et al. 2021	Microbial-derived carbon components are critical for enhancing soil organic carbon in no-tillage croplands: A global perspective	2021/1/*	95			Random	Globe	Microbial-derived carbon components	soil organic carbon in no-tillage croplands	Reference list for meta-analysis1.
27	Borchard et al. 2019	Biochar, soil and land-use interactions that reduce nitrate leaching and N2O emissions: A meta-analysis	2019/02/15	88	608		Random	Globe	reduce nitrate leaching and N2O emissions	Biochar, soil and land-use interactions	DOI
28	Chen et al. 2018	Different responses of soil organic carbon fractions to additions of nitrogen	2018/7/*	36	296		Random	Globe	additions of nitrogen	Different responses of soil organic carbon fractions	Supplementary references
29	Chen et al. 2018	The long-term role of organic amendments in building soil nutrient fertility: a meta-analysis and review	2018/01/04	132	541	20 countries, 122study sites	Random	Globe	organic amendments	building soil nutrient fertility	References
30	Luo et al. 2019	Understanding how long-term organic amendments increase soil phosphatase activities: Insight into phoD- and phoC-harboring functional microbial populations	2019/12/*	106	599		Random	Globe	phoD- and phoC-harboring functional microbial populations organic amendments	soil phosphatase activities	References
31	Mo et al. 2020	How plastic mulching affects net primary productivity, soil C fluxes and organic carbon balance in dry agroecosystems in China	2020/8/*	144	1906	92	Random	China	plastic mulching	net primary productivity, soil C fluxes and organic carbon balance in dry agroecosystems	REFERENCES
32	Xu et al. 2019	A global meta-analysis of soil organic carbon response to corn stover removal	2019/05/26	74	409	74 expt sites,	Random	Globe	corn stover removal	soil organic carbon response	DOI
33	Huang et al. 2021	Soil organic carbon, total nitrogen, available nutrients, and yield under different straw returning methods	2021/10/*	420	6820		Random	China	different straw returning methods	Soil organic carbon, total nitrogen, available nutrients, and yield	

34	Lu et al. 2021	Decrease in soil pH has greater effects than increase in above-ground carbon inputs on soil organic carbon in terrestrial ecosystems of China under nitrogen enrichment	2021/11/*	234			Random	China	Decrease in soil pH increases in above-ground carbon inputs	soil organic carbon in terrestrial ecosystems of China	The list of 234 papers from which the data were extracted for this meta-analysis.
35	Xu et al. 2020	Long-term, amplified responses of soil organic carbon to nitrogen addition worldwide	2020/12/*	476			Random	Globe	nitrogen addition	soil organic carbon	Article
36	Geng et al. 2023	Legumes can increase the yield of subsequent wheat with or without grain harvesting compared to Gramineae crops: A meta-analysis	2023/1/*	62	453	5 continents, 18 countries	Random	Globe	yield of subsequent wheat	Legumes	Reference
37	Li et al. 2023	The role of conservation agriculture practices in mitigating N2O emissions: A meta-analysis	2023/09/04	73	281		Random	Globe	conservation agriculture practices	mitigating N2O emissions	References of the meta-analysis
38	Poeplau et al. 2015	Carbon sequestration in agricultural soils via cultivation of cover crops – A meta-analysis	2015/02/01	30	139	37sites, 139 plots	Random	Globe	cultivation of cover crops	Carbon sequestration in agricultural soils	
39	Santachiara et al. 2019	Nutritional and environmental effects on biological nitrogen fixation in soybean: A meta-analysis	2019/07/01	92	956		Random	Globe		Relative response to N fertilization, P, K and S, B, Ca, Fe and Zn	Appendix A. List of peer reviewed publications included in the meta-analysis across environmental or management factors.
40	Wang et al. 2018	Decreasing soil microbial diversity is associated with decreasing microbial biomass under nitrogen addition	2018/05/01	55	273		Random	Globe			References
41	Wang et al. 2024	Augmenting the stability of soil aggregate carbon with nutrient management in worldwide croplands	2024/08/15	269	2035		Random	Globe			Supplementary:
42	Alvarez et al. 2017	Cover crop effects on soils and subsequent crops in the pampas: A meta-analysis	2017/07/01	67	975	67	Random	Argentina			Supplementary material Table A (Bulk density)
43	Li et al. 2020	Residue retention promotes soil carbon accumulation in minimum tillage systems: Implications for conservation agriculture	2020/10/20	243	1928		Random	Globe			Reference list for meta-analysis
44	Liu et al. 2018	Climatic role of terrestrial ecosystem under elevated CO2: a bottom-up greenhouse gases budget	2018/05/07	169	1655		Random	Globe			List of 169 publications from which data were extracted for this analysis.
45	Muhammad et al. 2019	Regulation of soil CO2 and N2O emissions by cover crops: A meta-analysis	2019/09/01	48	>1000		Random	Globe			Appendix B. Information of location, climate and soil texture for each experimental

										site used for meta-analysis in this study
46	Xia et al. 2018	Trade-offs between soil carbon sequestration and reactive nitrogen losses under straw return in global agroecosystems	2018/10/08	363	3251		Random	Globe		Data S1. References of all datasets included in this meta-analysis.
47	Zhao et al 2017	Crop yields under no-till farming in China: A meta-analysis	2017/03/01	164	1006		Random	China		Selected references
48	Kan et al. 2021	Effects of experiment duration on carbon mineralization and accumulation under no-till	2021/05/01	21	57		Random	Globe		C and N mineralization of undisrupted and disrupted soil from different structural zones of conventional tillage and no-tillage systems in northern France
49	Sun et al. 2020	Climate drives global soil carbon sequestration and crop yield changes under conservation agriculture	2020/01/17	115	1970	138	Random	Globe		Lòpez-Fando & Pardo
50	Zheng et al. 2020	Drought shrinks terrestrial upland resilience to climate change	2020/07/30	128	1344		Random	Globe		List of 128 publications from which data were extracted for this analysis.
51	Jian et al. 2020	A calculator to quantify cover crop effects on soil health and productivity	2020/05/01	269	4024	269	Random	Globe		Impact of soil health management practices on soilborne pathogens, nematodes and root diseases of vegetable crops.
52	Liu et al. 2020	Increased soil release of greenhouse gases shrinks terrestrial carbon uptake enhancement under warming	2020/05/13	164	1845		Random	Globe		List of 164 references from which data were extracted for this analysis
53	Ye et al. 2019	Biochar effects on crop yields with and without fertilizer: A meta-analysis of field studies using separate controls	2019/09/16	56	264	64	Random	Globe		References
54	Zhang et al. 2019	Biochar amendment effects on the activities of soil carbon, nitrogen, and phosphorus hydrolytic enzymes: a meta-analysis	2019/06/10	43	401		Random	Globe		References
55	Deng et al. 2018	Positive responses of belowground C dynamics to nitrogen enrichment in China	2018/03/01	124	570	127	Random	Globe		A list of 63 papers from which the data were extracted for this meta-analysis.
56	Peixoto et al. 2020	Occasional tillage in no-tillage systems: A global meta-analysis	2020/11/25	68	588		Random	Globe		

57	Qiao et al. 2024	Legume rhizodeposition promotes nitrogen fixation by soil microbiota under crop diversification	2024/04/04				Random	China			
58	Bebber et al. 2022	A meta-analysis of the effect of organic and mineral fertilizers on soil microbial diversity	2022/07/01	37	65		Random	Globe			SUPPLEMENTARY REFERENCES
59	Borchard et al. 2019	Biochar, soil and land-use interactions that reduce nitrate leaching and N2O emissions: A meta-analysis	2019/02/15	88	608		Random	Globe			
60	Venter et al. 2016	The impact of crop rotation on soil microbial diversity: A meta-analysis	2016/07/01	20	281		Random	Globe			Study name
61	Davies-Barnard et al. 2020	The Global Distribution of Biological Nitrogen Fixation in Terrestrial Natural Ecosystems	2020/02/09	142	252		Random	Globe			Text S1.
62	Biederman et al. 2012	Biochar and its effects on plant productivity and nutrient cycling: a meta-analysis	2012/12/31	371	941		Random	Globe			Author
63	Estrada-Carmona et al. 2022	Complex agricultural landscapes host more biodiversity than simple ones: A global meta-analysis	2022/09/12	157	1134	29 countries sites	Random	Globe			References
64	Zhao et al. 2020	Does crop rotation yield more in China? A meta-analysis	2020/01/01	45	214		Random	China			
65	Hu et al. 2022	Responses of AM fungal abundance to the drivers of global climate change: A meta-analysis	2022/01/20	75	431		Random	Globe			Note S1 Reference list of articles included in this meta-analysis.
66	Das et al. 2022	Responses of soil organic carbon to conservation practices including climate-smart agriculture in tropical and subtropical regions: A meta-analysis	2022/01/20	84	516		Random	Globe			Supplementary Data.1: References of the articles used for data
67	Geisseler et al. 2017	Effect of fertilization on soil microorganisms in paddy rice systems e A meta-analysis	2017/12/01	55			Random	China, India, Korea, Pakistan, Taiwan			References
68	Kim et al. 2020	Do cover crops benefit soil microbiome? A meta-analysis of current research	2020/03/01	60	30		Random	Globe			
69	Liu et al. 2019	Effect of Straw Retention on Crop Yield, Soil Properties, Water Use Efficiency and Greenhouse Gas Emission in China: A Meta-Analysis	2019/07/12	176	7417		Random	Globe			The References of yield data.

70	Song et al. 2017	Altered soil carbon and nitrogen cycles due to the freeze-thaw effect: A meta-analysis	2017/06/01	46			Random	Globe			Global Change Biology 20, 2663-2673.
71	Zeng et al. 2016	Nitrogen fertilization directly affects soil bacterial diversity and indirectly affects bacterial community composition	2016/01/01				Random	Inner Mongolia, China			
72	Zhou et al. 2017	Changes in microbial biomass and the metabolic quotient with biochar addition to agricultural soils: A Meta-analysis	2017/02/15	97	1073		Random	Globe			Reference:
73	Li et al. 2024	Soil N2O emissions from specialty crop systems: A global estimation and meta-analysis	2024/03/12	114	1137		Random	Globe			Reference
74	Zhao et al. 2022	Global systematic review with meta-analysis reveals yield advantage of legume-based rotations and its drivers	2022/08/22	462	11768	53 countri es	Random	Globe			Supplementary references
75	Yangjin et al. 2021	A meta-analysis of management practices for simultaneously mitigating N2O and NO emissions from agricultural soils	2021/09/01	39	952		Random	Globe			Agric Ecosys Environ 121:383-394
76	Shakoor et al. 2021	A global meta-analysis of greenhouse gases emission and crop yield under no-tillage as compared to conventional tillage	2021/01/01	50	431		Random	Globe			Rutkowska et al. (2018)
77	Xu et al. 2020	Intercropping maize and soybean increases efficiency of land and fertilizer nitrogen use; A meta-analysis	2020/02/01	88	1436		Random	Globe			
78	Raseduzzaman et al. 2017	Does intercropping enhance yield stability in arable crop production? A meta-analysis	2017/11/01	37			Random	Globe			
79	Abdalla et al. 2019	A critical review of the impacts of cover crops on nitrogen leaching, net greenhouse gas balance and crop productivity	2019/07/04	106		372	Random	Globe	cover crops	N leaching, net greenhouse gas balance and crop productivity	References
80	Bai et al. 2018	Effects of agricultural management practices on soil quality: A review of long-term experiments for Europe and China	2018/10/01	326	474	13 case sites	Random	Europe and China	agricultural management practices	soil quality	Title of paper
81	Chen et al. 2019	Effects of plant diversity on soil carbon in diverse ecosystems: a global meta-analysis	2019/10/18	121	1001		Random	Globe	plant diversity	soil carbon	Reference
82	Cheng et al. 2017	Warming enhances old organic carbon decomposition through altering functional microbial communities	2017/04/21	43			Random	Globe	Warming	Altering functional microbial communities	Reference

83	Cong et al. 2018	Impact of soil properties on the soil methane flux response to biochar addition: a meta-analysis	2018/08/24				Random	Globe	soil properties	soil methane flux response to biochar addition	
84	Davidson et al. 2017	Livestock grazing alters multiple ecosystem properties and services in salt marshes: a meta-analysis	2017/02/22	89	498		Random	Globe	Livestock grazing	multiple ecosystem properties and services in salt marshes	
85	Ding et al. 2017	A meta-analysis and critical evaluation of influencing factors on soil carbon priming following biochar amendment	2017/12/22	27	1170		Random	Globe	biochar amendment	influencing factors on soil carbon priming	
86	Du et al. 2017	The effect of no-till on organic C storage in Chinese soils should not be overemphasized: A meta-analysis	2017/01/02	95	409	57	Random	Globe	no-till	Organic C storage in soils	References
87	Elias et al. 2018	A meta-analysis of pesticide loss in runoff under conventional tillage and no-till management	2018/01/12	35			Random	Globe	conventional tillage and no-till	pesticide loss in runoff	reference
88	Gurevitch et al. 2018	Meta-analysis and the science of research synthesis	2018/03/08				Random	Globe			
89	Li et al. 2018	Liming effects on soil pH and crop yield depend on lime material type, application method and rate, and crop species: a global meta-analysis	2018/08/23	175	1337		Random	Globe	lime material type, application method and rate, and crop species	soil pH and crop yield	List of references used for the meta-analysis
90	Li et al. 2017	Long-term (≥20 years) application of fertilizers and straw return enhances soil carbon storage: a meta-analysis	2017/06/30	61	440		Random	China	Long-term (≥20 years) application of fertilizers and straw return	soil carbon storage	
91	Ma et al. 2018	Impacts of plastic film mulching on crop yields, soil water, nitrate, and organic carbon in Northwestern China: A meta-analysis	2018/04/01	83	1278		Random	China	plastic film mulching	crop yields, soil water, nitrate, and organic carbon	
92	Masuda et al. 2024	Global soil metagenomics reveals distribution and predominance of Deltaproteobacteria in nitrogen-fixing microbiome	2024/05/24	22	1451		Random	Globe			REFERENCES
93	Meurer et al. 2018	Tillage intensity affects total SOC stocks in boreo-temperate regions only in the topsoil—A systematic review using an ESM approach	2018/02/01	101			Random	Globe			References
94	Ren et al. 2018a	Responses of soil total microbial biomass and community compositions to rainfall reductions	2018/08/01	114	208		Random	Globe			Supplementary material (ii): Text S1：Studies included in the meta-analysis

95	Ren et al. 2018b	A synthetic analysis of livestock manure substitution effects on organic carbon changes in China's arable topsoil	2018/12/01	148	729	69	Random	China			References
96	Ros et al. 2016	Selenium fertilization strategies for bio-fortification of food: an agro-ecosystem approach	2016/02/19	243	3865		Random	Globe			Further study: papers used for the meta-analysis
97	Schmidt et al. 2013	Methods for second order meta-analysis and illustrative applications	2013/07/01				Random	Globe			
98	Sun et al. 2019	Priming of soil organic carbon decomposition induced by exogenous organic carbon input: a meta-analysis	2019/08/08	94	2048		Random	Globe			Text S1 Studies included in the current meta-analysis
99	Tian et al. 2018	Cropland abandonment enhances soil inorganic nitrogen retention and carbon stock in China: A meta-analysis	2018/08/21	83	295		Random	Globe			Note S1. A list of 83 publications used for collecting data in our meta-analysis
100	Vicente-Vicente et al. 2016	Soil carbon sequestration rates under Mediterranean woody crops using recommended management practices: A meta-analysis	2016/11/01	51	144		Random	Globe			Appendix A. References of the meta-analysis
101	Yuan et al. 2017	Experimental and observational studies find contrasting responses of soil nutrients to climate change	2017/06/01	323	1421	1346	Random	Globe			
102	Yue et al. 2017	Influence of multiple global change drivers on terrestrial carbon storage: additive effects are common	2017/03/28	633	3620		Random	Globe			Text S1 A list of 633 primary articles from which the data were extracted for this
103	Zheng et al. 2019	Global pattern and controls of biological nitrogen fixation under nutrient enrichment: A meta-analysis	2019/05/23	516			Random	Globe			APPENDIX: DATA SOURCES FOR META-ANALYSIS
104	Zhou et al. 2016	Changes in organic carbon and nitrogen in soil with metal pollution by Cd, Cu, Pb and Zn: a meta-analysis	2016/03/16	160	1187		Random	Globe			References

[1]Dropdown was created to show the list of all the references used in the original meta-analysis articles (Excel files) only if the list was provided by the original authors.

Table S2. The logistic steps in searching for meta-analysis articles on triple-goal system (more food, healthy soil, less emission) and preset criteria for selecting articles to be included in the SOMA.

Meta-analysis search on triple-goal articles (more food, healthy soil, less emission)

	# of meta-analysis article
Meta-analysis in agriculture or cropping	4712
Emission or N_2O or soil property or soil health or crop yield	1805
between 2015-2024	1544
emission or N_2O	385
emission AND soil health or property	190
emission AND soil health/property AND crop yield	**104**

<u>Preset selection criteria</u>

(1) analyzed the effect of one or several factors on at least two of the three factors (more food, healthier soil, fewer emissions);

(2) presented a statistical analysis of at least two primary studies on at least two of the three factors;

(3) reported indicators of precision of the effect sizes (standard errors, standard deviation, or confidence intervals);

(4) provided details on the methods used in the original studies which can be found in the paper or supplementary files.

Table S3. List of examples of using crop diversification (such as intercropping, diversified rotation), conservation agricultural practices, and improved cropping management, for fulfilling the 'triple-goal' system, i.e., more food, healthier soil, fewer emissions.

Coordinators	Study site	Study year or data reported	Crop	Soil & crop management	Physiobiological mechanism	Outcome in crop productivity and/or soil property change	Reference
**Intercropping**							
–8.05; –34.90	Recife, Brazil	Apr-23	Cactus, millet	Cactus–millet intercropping	Improved between-crops interactions	WUE ranged from 2.31–2.47 kg m^{-3}, significantly greater than sole crops	Souza et al., 2023
37.90; 102.77	Wuwei, China	Nov-20	Maize, wheat	Maize–wheat intercropping	Promoted water complementation and inter-zone water migration	Maize–wheat intercropping increased WUE by 20–50%	Yin et al., 2020
36.72; 3.15	D'Alger, Algeria	Apr-23	Chickpea, durum wheat	Chickpea–durum wheat intercropping	Promoted WUE and NUE	WUE of 0.62 kg m^{-3}, higher than sole crops	Kherif et al., 2023
36.71; 3.15	D'Alger, Algeria	Feb-23	Chickpea, wheat	Chickpea–wheat intercropping	Increased chlorophyll content, optimizing WUE	Increased WUE by 0.30–0.57 kg m^{-3} compared to sole chickpea	Bouras et al., 2023
29.63; 52.52	Shiraz, Iran	Jun-23	Chickpea, barley	Chickpea–barley intercropping	Enhanced chlorophyll content, leaf carotenoid content, and catalase and peroxidase activities	Enhanced WUE compared to sole crops	Assadi et al., 2023
29.39; 71.69	Bahawalpur, Pakistan	Nov-22	Maize, soybean	Maize–soybean strip intercropping	Species complementarities for radiation, water, and land in time and space, higher LAI, radiation use	Intercropped maize had WUE ranging from 13.3–16.2 kg ha^{-1} mm^{-1}, higher than sole crops	Raza et al., 2022
40.63; 22.96	Thessaloniki, Greece	Feb-21	Wheat, pea	Wheat–pea intercropping	Increased land equivalent ratio (LER)	Used available water more efficiently, resulting in higher yields	Pankou et al., 2021
**Crop rotation**							
–34.97; 138.6	Urrbrae, Australia	Jan-21	Cereals, legumes, oilseeds	Cereal–legume; soybean–sunflower, chickpea–flax	Promoted between species complementary, competitive and interactions; allelopathic pest repellence	Cereal–legume intercropping had higher carbon yield than soybean–sunflower and chickpea–flax cropping	Dowling et al., 2021
35.81; 50.95	Karaj, Iran	Nov-21	Sorghum, amaranth	Sorghum–amaranth intercropping	Partial root-zone irrigation; alternate furrow irrigation	Saved 20–22% irrigation water without reducing carbon yield	Baghdadi et al., 2021
–1.26; 36.82	Sub-Saharan Africa, Kenya	Nov-20	Potato, dolichos (*Lablab purpureus* L.)	Potato–dolichos or potato–hairy vetch intercropping	Increased proline (1.99–2.91 vs. 1–1.19 umol g^{-1}) and soluble carbohydrates (28–59 vs. 10–28 umol g^{-1}) under stress	Potato–legume intercropping reduced nutrient losses by 45–80%, increased yields by 2–3-fold and WUE by 2–4-fold in potato	Nyawade et al., 2020
37.97; –100.8	Garden City, Kansas	Nov-22	Winter wheat, sorghum	Wheat–forage sorghum–forage old rotation	Diversification increased cropping intensity, productivity, resource use, and gross margin	Double cropping of forage sorghum after wheat increased total carbon production	Holman et al., 2022
**Soil mulching**							
34.65; 110.53	Yangling, Northwest China	Nov-21	Wheat	Plastic mulching	Improved RUE post-anthesis, elevated canopy photosynthesis,	Increased transpiration-to-evaporation ratio, carbon source size, and sink size	Ding et al., 2021

					delayed RUE peak in reproductive period		
34.65; 110.53	Yangling, Northwest China	Jan-22	Winter wheat	Ridge–furrow combined with supplemental irrigation	Increased soil water storage and net photosynthetic rate	Decreased irrigation water by 50% without reducing crop yield	Zhang et al., 2022
34.65; 110.53	Yangling, Northwest China	Sep-22	Maize	Partial straw mulching with urea blending	Delayed leaf senescence, increased chlorophyll content, photosynthesis, and N uptake	Increased maize grain yield by 11–53% and WUE by 9–57%	Guo et al., 2022
34.65; 110.53	Yangling, Northwest China	Aug-21	Winter wheat	Ridge–furrow with optimum irrigation and N rate	Increased LAI, above-ground DW, leaf chlorophyll content, and net photosynthetic rate	Increased winter wheat yield by 14.6–17.7%, WUE by 5.0–10.0%, and NUE by 16.2–30.5%	Gu et al., 2021
**Improved irrigation**							
–3.73; –38.53	Fortaleza, Ceara, Brazil	Apr-22	Maize	Supplemental irrigation with brackish water	Reduced water stress, averted excessive salt accumulation in soil, improved CO_2 assimilation rates	Promoted physical water productivity by 1.3–3.0-fold	Cavalcante et al., 2022
31.66; 36.31	Northern Jordan	Jan-21	Pistachio (*Pistacia vera* L.)	Micro-catchment and gravel mulching	Improved soil moisture, photosynthesis (Pn), transpiration (E), and stomatal conductance	Improved carbon production in young pistachio trees in rainfed regime	Tadros et al., 2021
17.46; 78.45	Hyderabad, India	Oct-22	Cotton (*Gossypium* sp.)	Cotton in an eddy covariance system	Seasonal NEE (–333 to –392 g C m^{-2}), GPP (990–1064 g C m^{-2}), R-eco (656–672 g C m^{-2}), and ET (468–545 mm)	Increased net CO_2 sink, with seasonal ec-WUE 1.9–2.1 g C kg^{-1} of H_2O	Chakraborty et al., 2022
**Subsoil tillage**							
35.20; 110.11	Heyang, Shaanxi	May-20	Wheat, maize	Subsoil tillage alternated with plowing	Reduced bulk density by 5.19%, increased porosity 5.69% and macroaggregates by 26.92%	Promoted WUE by 7.25% and yield by 8.37%	Yu et al., 2020
34.65; 110.53	Yangling, Northwest China	Jul-21	Wheat, maize	Wheat–maize rotation with subsoil tillage	Reduced precipitation loss, maintained soil water balance by regulating water use and precipitation storage	Reduced precipitation loss by 78–135 mm during the fallow period, increased WUE by 6.5–10.8 kg ha^{-1} mm^{-1}, and grain yields	Li et al., 2021
**Conservation agriculture**							
50°03′N - 112°09′W	Vauxhall, Canada	2000-2011	Multiple rotation crops	Conventional tillage Reduced tillage	Reduced tillage increased soil microbial biomass carbon, fungal and bacterial PLFAs	Reduced tillage increased soil microbial biomass C from 428 to 509 mg C kg^{-1}.	[6]
36°20′N - 120°7′W	California, USA	1999-2013	Tomato cotton	No-tillage (NT), Conventional tillage (CT)	CT reduced number of bacteria and archaea in top 5-cm soil layer.	Tillage reduced bacteria and archaea about 4×10^7 and 1.5×10^7 in top 5cm soil layer.	[7]

35°25′N - 82°33′W	North Carolina, USA	1994-2009	Multiple species	No-tillage, CT	No-till increased PLFA	No-till increased 55% of total PLFA concentration and 22% of bacterial PLFA compared to CT	[8]
38°54'N - 77°2'W 45°29'N - 115°27'W	Washington and Idaho, USA	35 years before first sampling in 2008 at Washington, and 2000-2010 at Idaho	Winter wheat, spring wheat, Barley, legume	No-tillage; Conventional tillage	Tillage didn’t influence soil bacterial diversity, but impacted bacterial community composition.	Tillage strongly impacted bacterial community composition. In particular, *Chitinophagaceae, Micrococcaceae, Gaiellaceae* and *Nocardioidaceae* were more frequent under tillage, while *Hyphomicrobiaceae, Koribacteraceae, Acidobacteriaceae* dominant under NT.	[9]
33°52’N - 83°27’W	Watkinsville, USA	1994-2013	Cotton, corn, rye, wheat, canola, pearl, millet	No-tillage; Conventional tillage	CT reduced the amount of AOA and AOB in top 20-cm soil layer.	CT reduced the amount of AOA and AOB in top 20-cm soil layer, which could slow down the potentials of N mineralization.	[10]
35°57’N - 98°03’W	El Reno, USA	2011-2017	Wheat, legume	No-tillage; Conventional tillage	NT increased fungal biomass carbon with inorganic N added.	Fungal biomass carbon under NT 193% higher with inorganic N added but 26% lower with organic N added, then under CT. NT+inorganic N increased 91.3% of FBC, while CT+organic N increased 93.3% of FBC.	[11]
32°31′N - 89°42′W	Mississippi, USA	2000-2014	Balansa clower, Abruzzi rye, cotton as cover crop	No-tillage; Reduced tillage	No till increased soil microbial biomass C.	No-till increased soil microbial biomass C (581.13 mg per kg dry soil) than reduced till (473.87 mg per kg dry soil). Relative abundance of *Betaproteobacteria* and *Azoarcus* higher under NT, but *Alphaproteobacteria*, *Rhizobiales, Sphingomonas* and *Bacillus* were higher under reduced till.	[12]
37°30'N - 13°31'E	Sicily, Italy	1991-2009	Wheat, bean	No-tillage (NT), Conventional tillage (CT), Reduced tillage (RT)	NT increased soil microbial carbon in wheat-pulse rotation	Both RT and NT increased MBC/TOC and reduced qCO_2 around 0.3 mg CO_2-C g^{-1} MBC h^{-1}. NT increased SMBC 120-200 mg kg^{-1} in wheat-pulse rotation, then CT.	[13]

51°47'N - 11°43'E	Bernburg, Germany	1992-2015	Maize, wheat, barley, rapeseed	Conventional tillage; Mouldboard plough tillage (MP)	CT increased the number of genera belonging to *Alphaproteobacteria* (e.g., *Amaricoccus, Chelatococcus, Microvirga)* and *Actinobacteria (Gaiella, Janibacter, Rubrobacter).*	CT increased the genera *Alphaproteobacteria* (e.g., *Amaricoccus, Chelatococcus, Microvirga)* and *Actinobacteria* , compared with MP.	14
51°47'N - 11°43'E	Bernburg, Germany	1992-2015	Maize, wheat, barley, rapeseed	Conventional tillage, Mouldboard plough tillage	CT selected fungi such as *Stagonospora, Claroideoglomus*, and *Rhizophagus*, while MP selected *Entrophospora* and *Sebacina*.	CT selected fungi such as *Stagonospora, Claroideoglomus*, and *Rhizophagus* in wheat rhizosphere, while MP selected *Entrophospora* and *Sebacina*.	15
48°00'N - 2°49'E	Brittany, France	1993-2013	Legumes	No-tillage, CT	Tillage significantly decreased soil microbial biomass and fungal richness.	CT decreased soil microbial biomass and fungal richness but increased bacterial richness and evenness compared to NT.	16
48°48′N - 2°08′E	Versailles, France	1997-2010	Wheat, pea, oilseed, maize	Conventional tillage, Conservation tillage	Conservation tillage increased soil bacteria and fungi compared to conventional tillage.	Conservation tillage increased $0.3\times\log_{10}$ g^{-1} and $0.25\times\log_{10}$ g^{-1} for soil bacteria and fungi respectively, compared to conventional tillage.	17

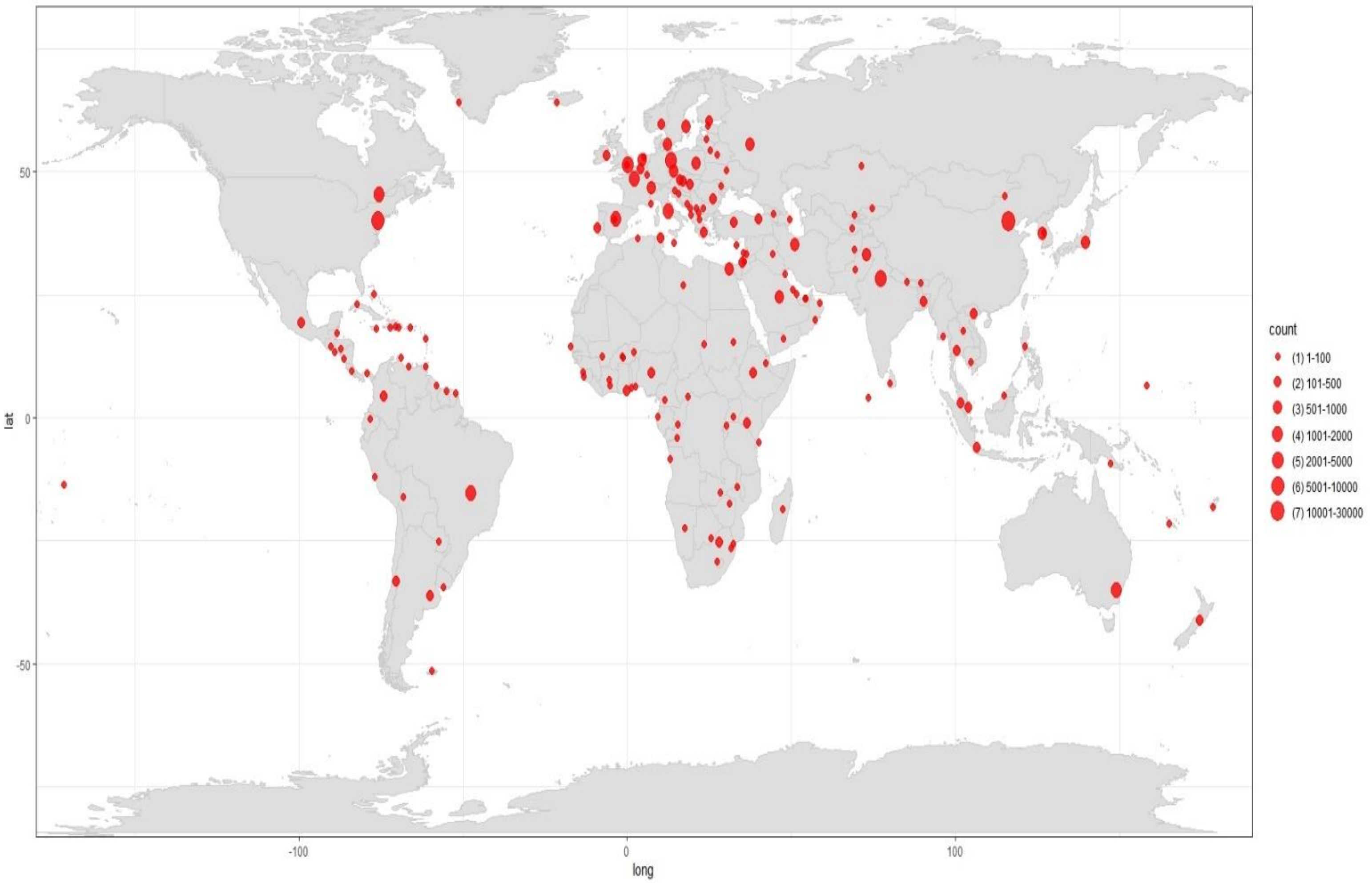

Figure S1. A global map showing the study sites reported in the 104 original first-order meta-analysis articles. The red dots represent approximate sampling sites with the dot size denoting the range of the number of studies covered in the given meta-analyses. Some dots with only a few studies are hardly visible.

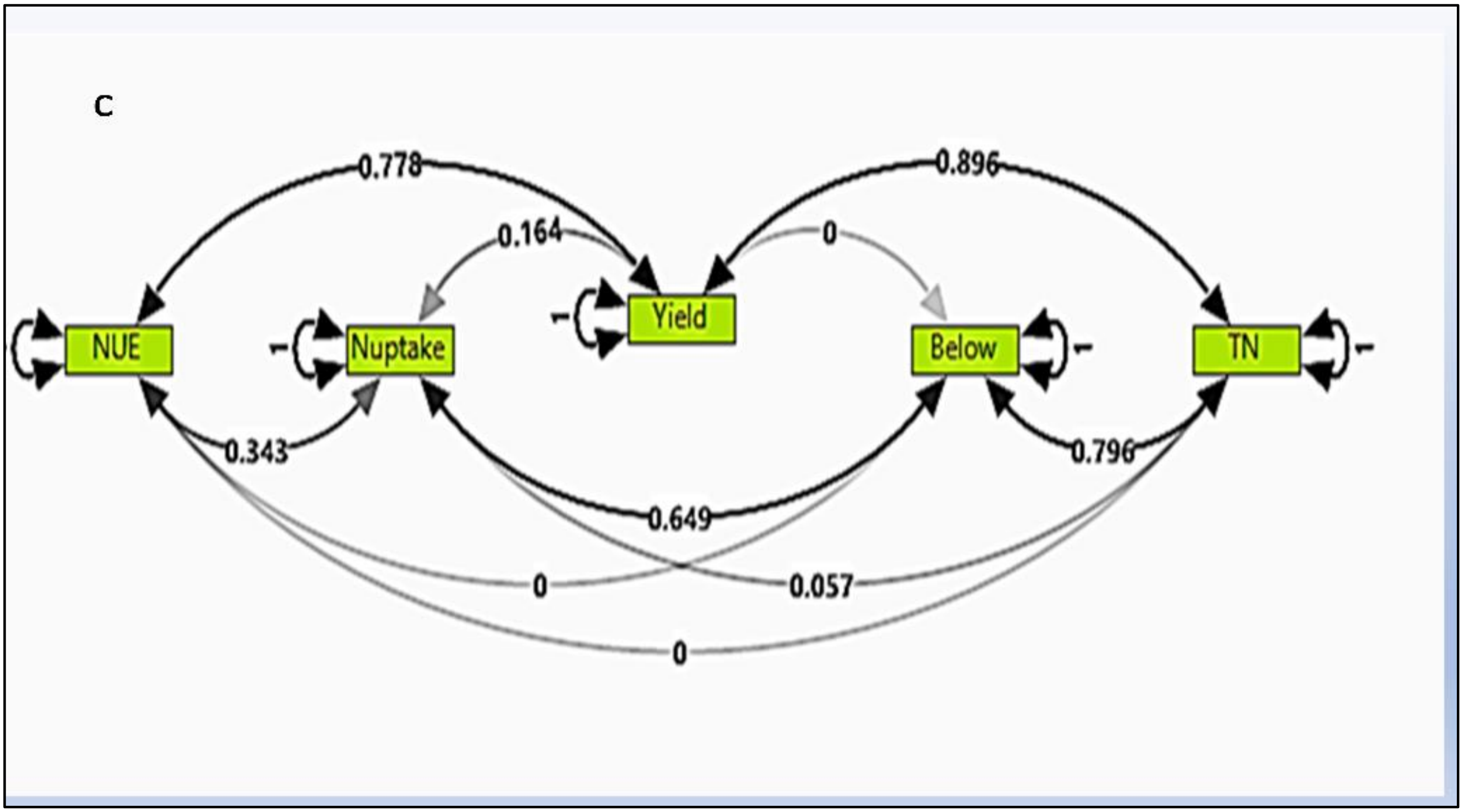

Figure S2. The '3-goal' system integrates existing and novel farming practices to maximize agrifood productivity and stability. Structure equation modeling demonstrates that increased systems productivity is closely linked to total N (TN) supplied and NUE among other factors, both being associated with plant N uptake (N uptake) and belowground biomass accumulation (below).

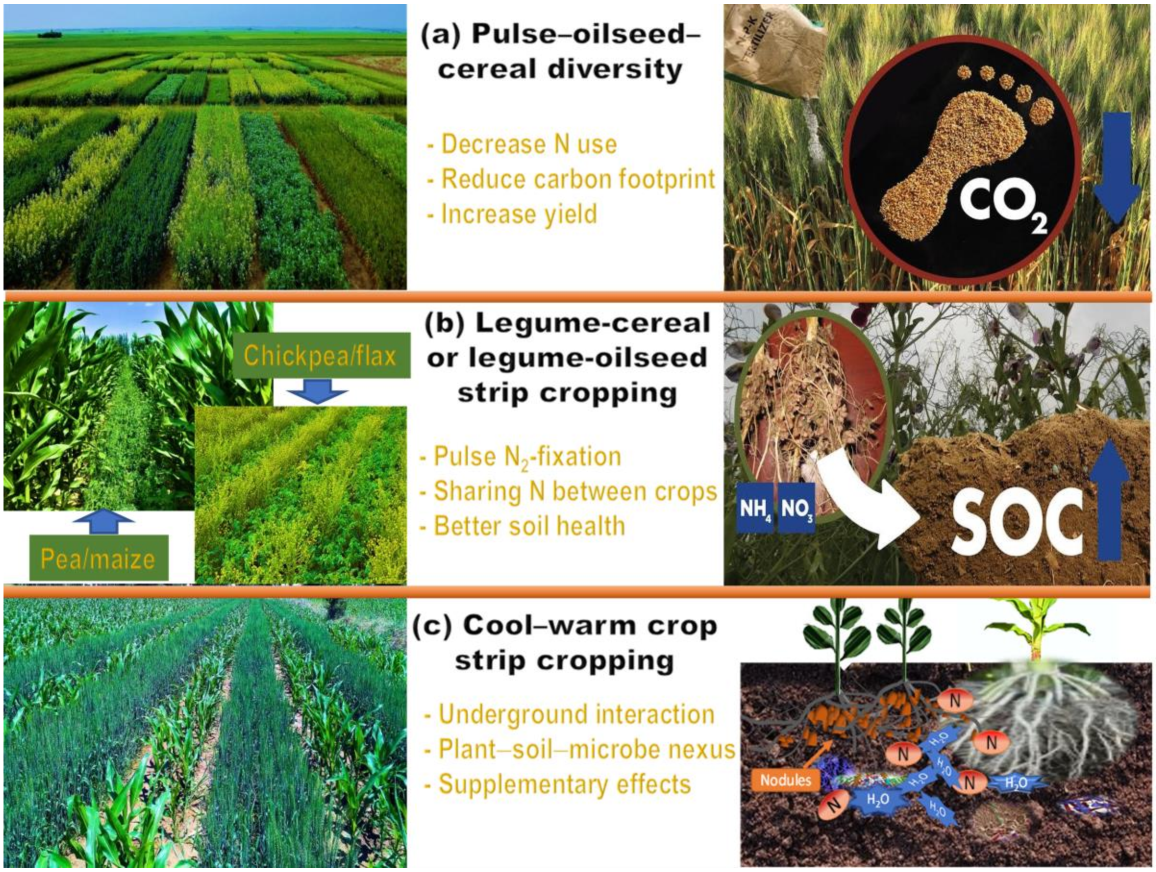

Figure S3. Legume-based cropping diversification provides significant benefits to soil health and environmental sustainability. For smallholders in less developed countries with smaller plots of land, legume-based rotation diversification offers an additional income source and can act as a safety net given low capacities to respond in the case of extreme weather events.

Legume-based diversification provides significant benefits to soil health. A meta-analysis with global data sources (77 articles, 393 treatments) revealed that crop diversification coupled with reduced or no tillage increased soil fungi abundance and fungi-to-bacteria ratios, favoring nutrient cycling and improving soil health. In the Indo-Gangetic Plains, incorporating annual legumes such as chickpea into maize–wheat and rice–wheat rotations increased SOC by 7–13%, particulate C by 41–95%, and labile C by 29–38% as compared to cereal-based monoculture. In temperate southwest France, temporally-grown legumes between cash crops lowered CO_2 emissions by 50–102% compared to a

cropping system without legumes. Shifting cropping priorities from traditional cereal-dominant systems to legume-based diversified systems can produce more plant-based protein, supporting a growing trend towards plant-based proteins globally.

Several meta-analyses that synthesized numerous field experiments revealed inconsistent effects. A meta-analysis of studies conducted across Mediterranean, tropical, subtropical and temperate monsoon climates in Spain and China showed that no-till (NT) with returning crop residues increased N_2O emissions by 6.1 to 12.9%[18]. Similarly, another global meta-analysis revealed that no-till increased CO_2, N_2O, and CH_4 emissions by 7.1, 12.0, and 20.8%, respectively, as compared to conventional tillage (CT)[19]. Increased N fertilization rates under NT management improved crop yield and GHGs emissions up to 23 and 58%, respectively, compared to CT.

Conversely, a meta-analysis with 151 direct comparisons between NT or reduced tillage (RT) and CT showed that NT/RT decreased soil N_2O emissions by 11% compared to CT in humid areas and in soils with carbon content < 20 g kg^{-1}[20].

A literature review of 37 published papers showed tillage intensity did not affect cumulative N_2O even after 38–40 years of tillage implementation, regardless of the use of a moldboard plow, chisel plow, double disk, or no-till practices[21].

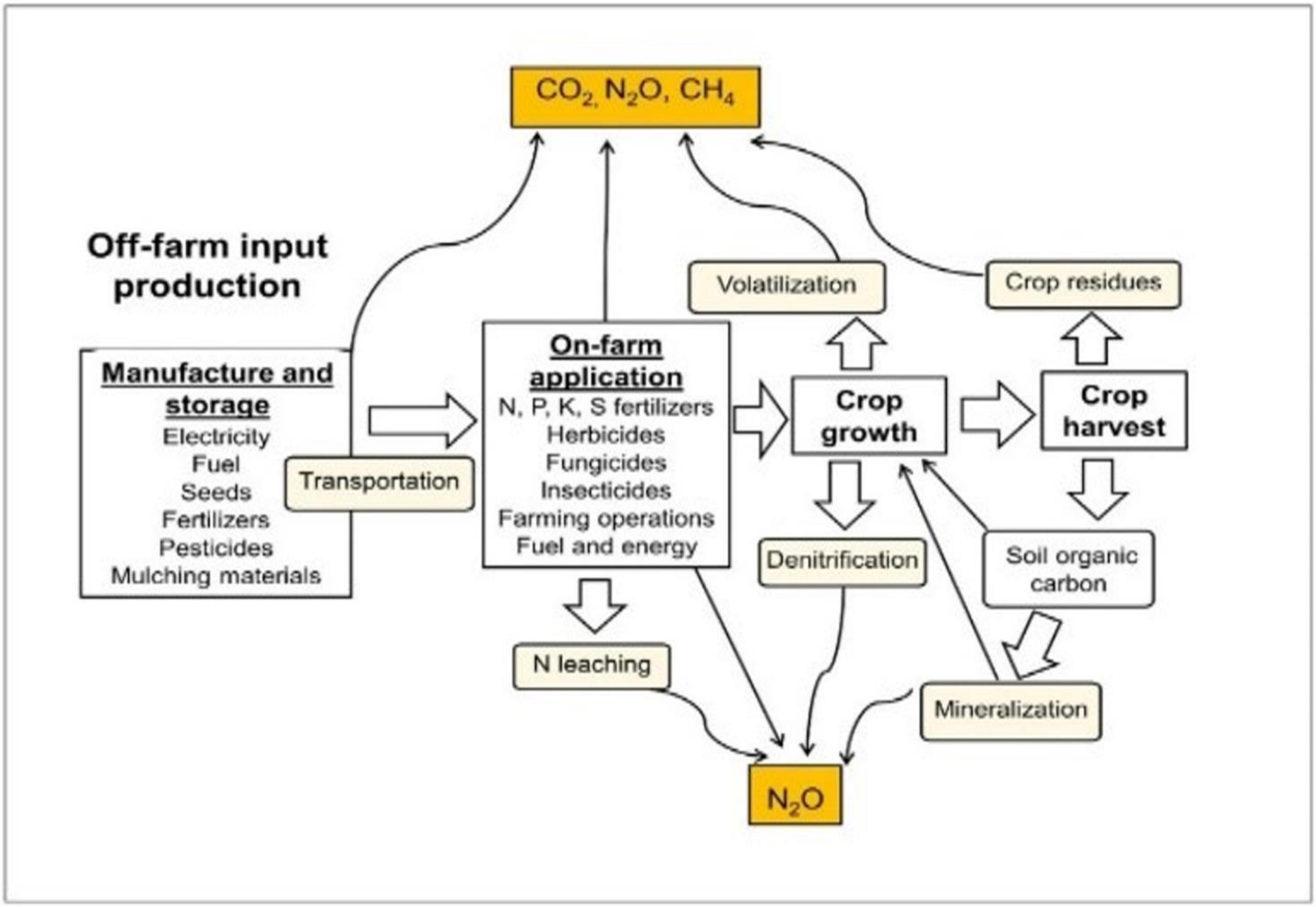

Figure S4. The boundary for estimating CO_2eq emissions in agrifood production system. The emissions include those from energy use and non-energy sources, with N_2O and CH_4 emissions converted to CO_2eq as defined in the IPCC guideline. The estimate formula was adapted and modified from Yang et al. (2024).

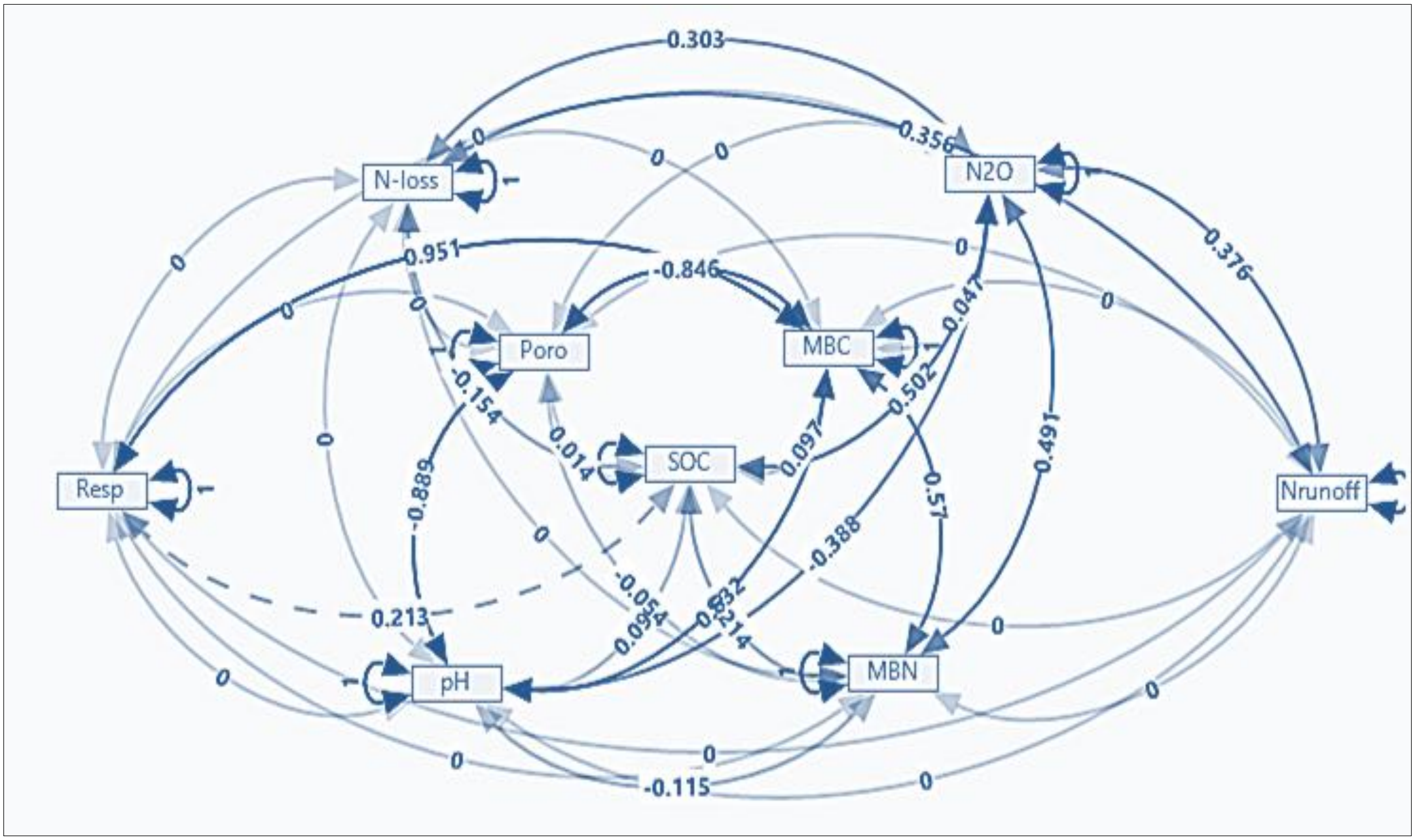

Figure S5. Structural equation modeling indicates that N_2O emissions during cropping phases are the main contributors to overall N loss. These emissions are positively correlated with N runoff (Nrunoff), whereas the total N loss is complicated by the interactive effect between soil porosity (poro) and microbial biomass carbon (MBC), MBC and microbial biomass nitrogen (MBN), N_2O and pH and porosity, and soil pH and SOC. Less important factors to the three goals have been excluded from the figure based on their correlation coefficients (the numbers besides the lines).

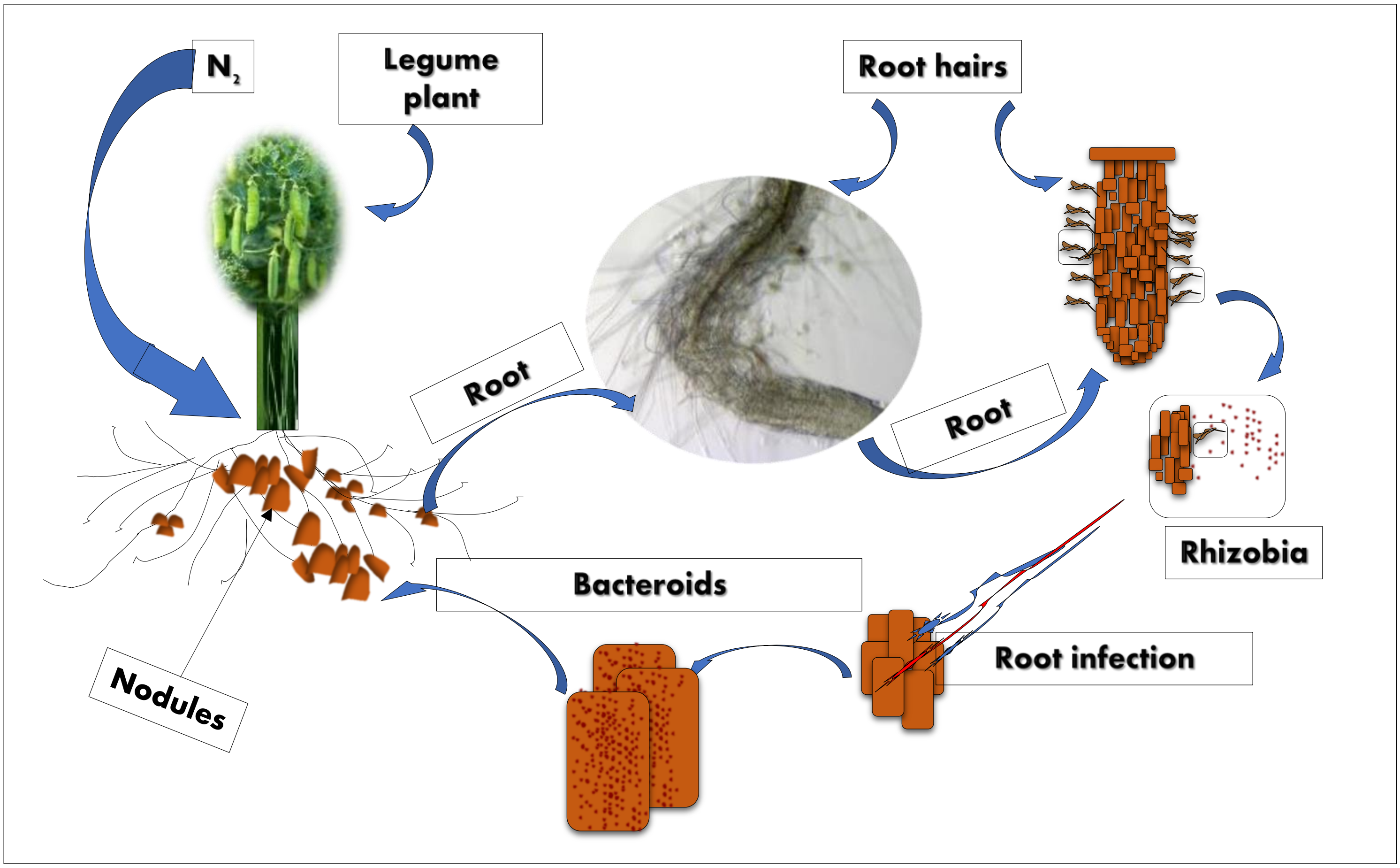

Figure S6. Rhizobial symbiosis in legume plant roots. Rhizobacteria enter the roots through root hairs to form rhizobia, and then go through root infection become bacteriods, and forms nodules on the infected sites on the root. Many nodules can be formed in the roots of legume plants to develop a mutual relationship between the bacterioids and root cells, and the symbiosis converts atmospheric N_2 into plant-available N. The symbiosis promotes ammonia secretion by bacteroides through regulating oxygen supply and catabolizing plant-derived dicarboxylates through root hairs as the energy and electron source donors for N_2 fixation, leading to conversion of atmospheric N_2 into NH_3.

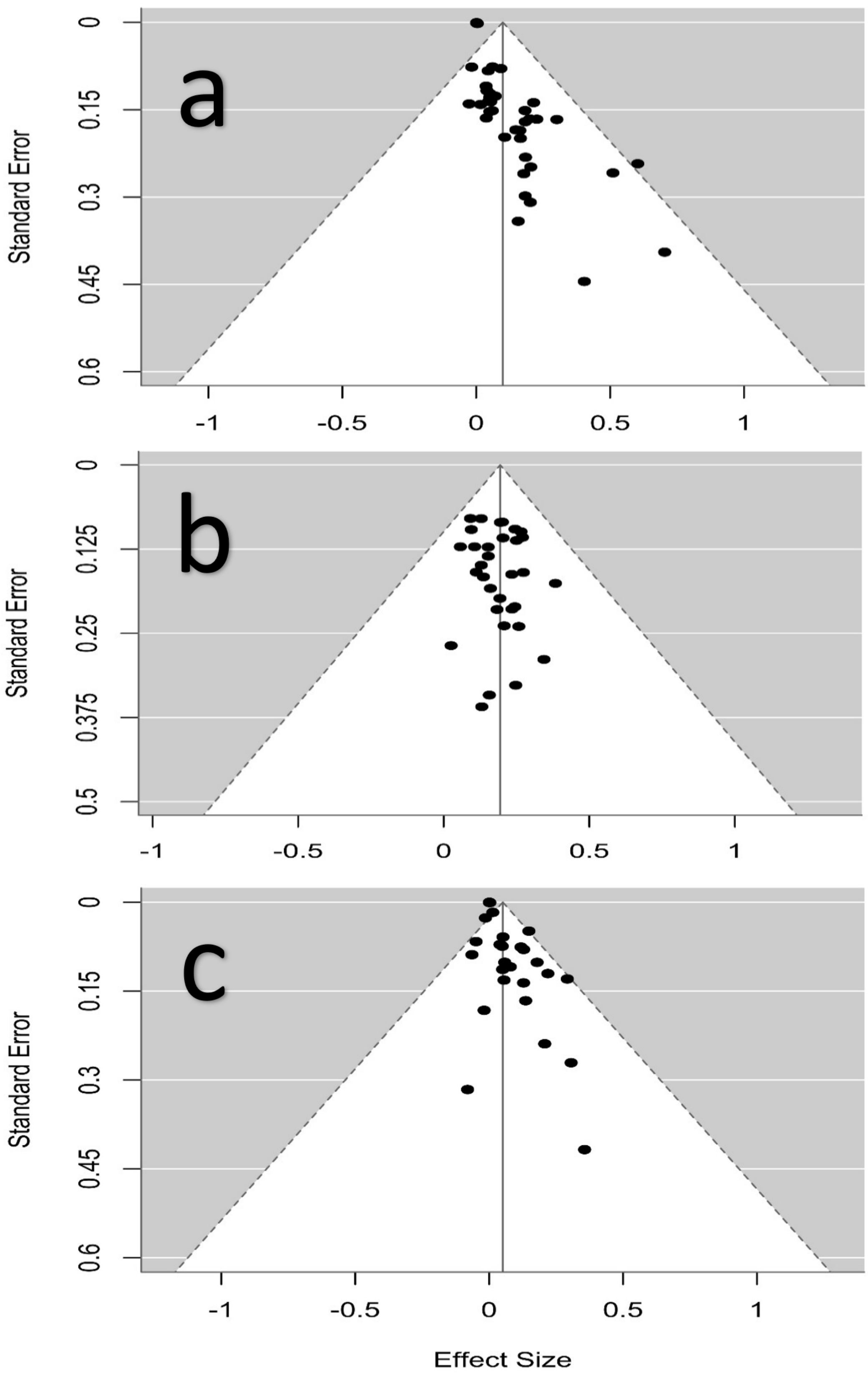

Figure S7. Funnel plots for (**a**) more food, (**b**) healthy soil, and (**c**) fewer emissions.
