# Supplementary material for: Integrated strategies for enhancing agrifood productivity, lowering greenhouse gas emissions, and improving soil health
Source: Innovation (Camb). 2025 Jun 25;6(11):101006. doi: 10.1016/j.xinn.2025.101006 (PMC12628179; doi:10.1016/j.xinn.2025.101006)
Supplement: Document S2. Article plus supplemental information [file mmc2.pdf]

REVIEW

OPEN ACCESS

www.the-innovation.org

Integrated strategies for enhancing agrifood productivity, lowering greenhouse gas emissions, and improving soil health

Li Wang,[1,2,3,*] Gina Marie Garland,[4,5] Tida Ge,[6,7] Shiqian Guo,[8] Endalkachew Abebe Kebede,[9] Chengang He,[10] Mohamed Hijri,[11,12] Daniel Plaza-Bonilla,[13] Lindsay C. Stringer,[14] Kyle Frankel Davis,[9,15] Soon-Jae Lee,[12,16] Shoujiang Feng,[1,2,3] Li Wang,[1,6,7] Zhenyang Wei,[1] Hanwen Cao,[1] Zhi Wang,[1] Jiexiong Xu,[1] Kadambot H.M. Siddique,[17] Gary Y. Gan,[1,2,3,18,*] and Min Zhao[1,2,3,*]

*Correspondence: li.wang@wzu.edu.cn (L.W.); gary.gan@ubc-soil.ca (G.Y.G.); zmcn@tom.com (M.Z.)

Received: November 9, 2024; Accepted: June 21, 2025; Published Online: June 25, 2025; https://doi.org/10.1016/j.xinn.2025.101006

© 2025 The Authors. Published by Elsevier Inc. on behalf of Youth Innovation Co., Ltd. This is an open access article under the CC BY license (http://creativecommons.org/licenses/by/4.0/).

GRAPHICAL ABSTRACT

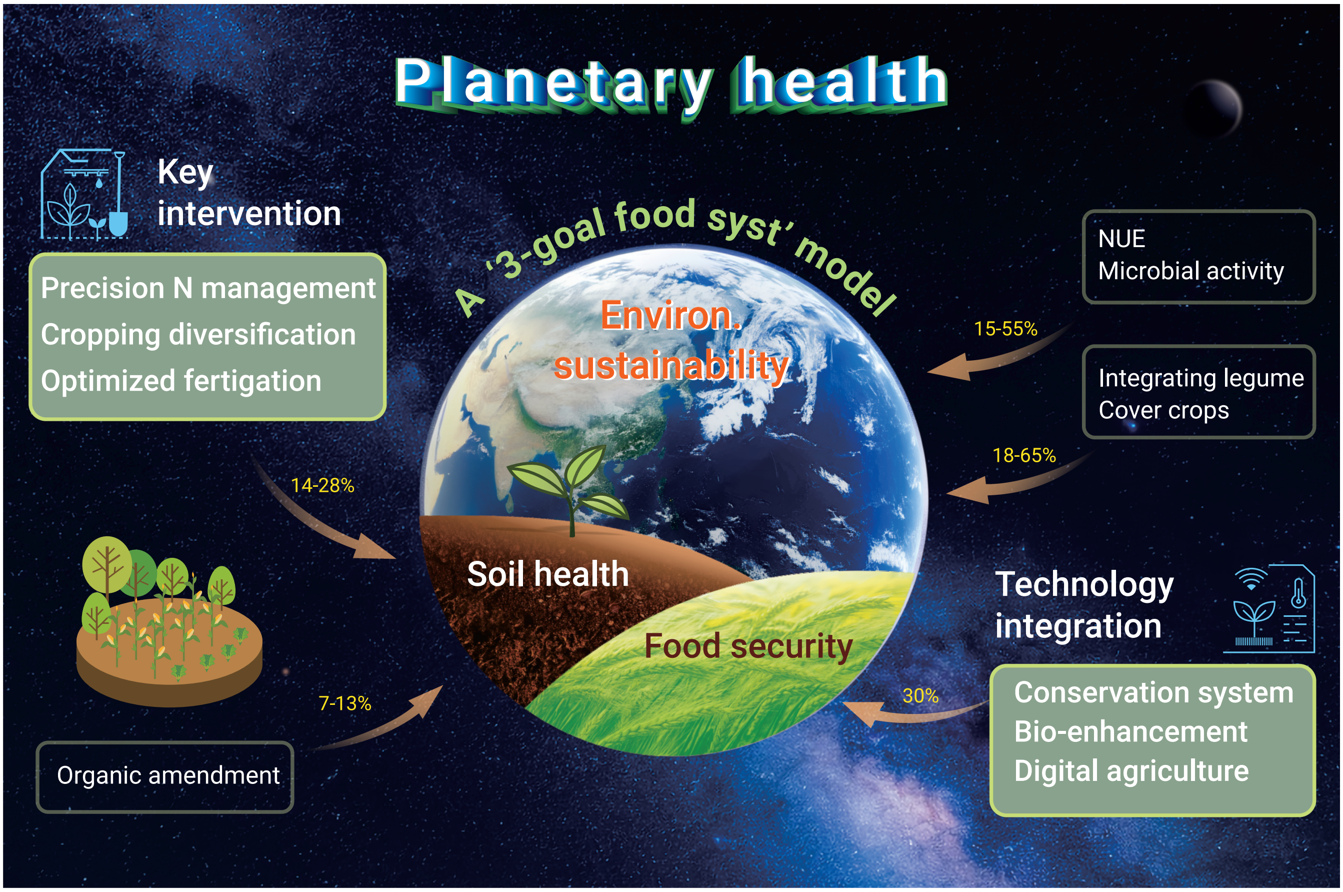

PUBLIC SUMMARY

- Integrated cropping strategies can simultaneously enhance food production, reduce emissions, and improve soil health.
- Enhancing plant-soil-microbe interactions can enhance agroecosystem resilience by 15%–40%.
- Prioritizing CO_2 fertilization, along with biofertilization, can cut greenhouse gas emissions by 30%–50%.
- Legume-cereal intercropping can enhance system productivity while reducing environmental footprint.
- Second-order meta-analysis can synthesize comprehensive research to solve interlinked issues.

OPEN ACCESS
REVIEW

Integrated strategies for enhancing agrifood productivity, lowering greenhouse gas emissions, and improving soil health

Li Wang,[1,2,3,*] Gina Marie Garland,[4,5] Tida Ge,[6,7] Shiqian Guo,[8] Endalkachew Abebe Kebede,[9] Chengang He,[10] Mohamed Hijri,[11,12] Daniel Plaza-Bonilla,[13] Lindsay C. Stringer,[14] Kyle Frankel Davis,[9,15] Soon-Jae Lee,[12,16] Shoujiang Feng,[1,2,3] Li Wang,[1,6,7] Zhenyang Wei,[1] Hanwen Cao,[1] Zhi Wang,[1] Jiexiong Xu,[1] Kadambot H.M. Siddique,[17] Gary Y. Gan,[1,2,3,18,*] and Min Zhao[1,2,3,*]

[1]College of Life and Environmental Science, Wenzhou University, Wenzhou 325035, China
[2]State & Local Joint Engineering Research Center for Ecological Treatment Technology of Urban Water Pollution, Wenzhou University, Wenzhou 325035, China
[3]Zhejiang Provincial Key Laboratory of Water Ecological Environment Treatment and Resource Protection , Wenzhou University, Wenzhou 325035, China
[4]Department of Environmental Systems Sciences, ETH Zurich, 8092 Zurich, Switzerland
[5]School of Integrative Plant Science, Cornell University, Ithaca, NY 14853, USA
[6]State Key Laboratory for Quality and Safety of Agro-Products, Key Laboratory of Biotechnology in Plant Protection of MARA, Institute of Plant Virology, Ningbo University, Ningbo 315211, China
[7]International Science and Technology Cooperation Base for the Regulation of Soil Biological Functions and One Health of Zhejiang Province, Ningbo University, Ningbo 315211, China
[8]Gansu Provincial General Station for Cultivated Land Quality Construction and Protection, Lanzhou 730030, China
[9]Department of Geography and Spatial Sciences, University of Delaware, Newark, DE 19716, USA
[10]College of Tobacco Science, Yunnan Agricultural University, Kunming 650031, China
[11]Institut de Recherche en Biologie Végétale, Département de Sciences Biologiques, Université de Montréal, Montréal, QC H1X 2B2, Canada
[12]African Genome Center, Université Mohammed VI Polytechnic (UM6P), Ben Guerir 43150, Morocco
[13]Department of Agricultural and Forest Science and Engineering – Agrotecnio-CERCA Center, Universitat de Lleida, 25198 Lleida, Spain
[14]York Environmental Sustainability Institute, and Department of Environment and Geography, University of York, York YO10 5DD, UK
[15]Department of Plant and Soil Sciences, University of Delaware, Newark, DE 19716, USA
[16]Department of Ecology and Evolution, University of Lausanne, 1015 Lausanne, Switzerland
[17]The UWA Institute of Agriculture, The University of Western Australia, Crawley, WA 6009, Australia
[18]Agroecosystems, The μBC-Soil Group, Tallus Heights, Kelowna, BC V4T 3M2, Canada
*Correspondence: li.wang@wzu.edu.cn (L.W.); gary.gan@ubc-soil.ca (G.Y.G.); zmcn@tom.com (M.Z.)
Received: November 9, 2024; Accepted: June 21, 2025; Published Online: June 25, 2025; https://doi.org/10.1016/j.xinn.2025.101006
© 2025 The Authors. Published by Elsevier Inc. on behalf of Youth Innovation Co., Ltd. This is an open access article under the CC BY license (http://creativecommons.org/licenses/by/4.0/).
Citation: Wang L., Garland G.M., Ge T., et al., (2025). Integrated strategies for enhancing agrifood productivity, lowering greenhouse gas emissions, and improving soil health. The Innovation **6(11)**, 101006.

Global agrifood systems face three interconnected challenges: ensuring food security, promoting environmental sustainability, and restoring soil health in the face of climate change. Conventional practices have prioritized productivity over ecological resilience, leading to soil degradation, increased greenhouse gas (GHG) emissions, and inefficient resource utilization. Here, we introduce a "triple-goal" agrifood framework that enhances food production, soil health, and GHG mitigation simultaneously through integrated innovations. Using a second-order meta-analysis of 104 meta-analyses that cover 39,162 studies and 300,139 global field comparisons, we identified key interventions, including optimized fertigation, diversified cropping systems, organic amendments, and precision N management, that increased productivity by 14%–28% while reducing environmental impacts. Diversified systems boosted yields by 19.6% and reduced land use by 19%. Integrating legumes and cover crops lowered N_2O emissions by 18%–65%, while organic amendments increased soil organic carbon stocks by 7%–13%. Structural equation modeling identified nitrogen use efficiency and microbial activity as central to the food-soil-emissions nexus. However, tradeoffs remain; yield-focused strategies can elevate emissions if not tailored to local conditions. By integrating agronomic, biological, and technological interventions such as conservation tillage, biofertilization, and digital agriculture, this triple-goal framework supports a 15%–30% reduction in anthropogenic CO_2-equivalent emissions. These findings underscore the need for policy reform and multi-stakeholder collaboration to scale up the adaptation of integrated strategies in alignment with the UN's Sustainable Development Goals and the "One Health" initiative. The triple-goal framework provides a transformative pathway to climate-smart, equitable, and resilient agrifood systems that strike a balance between productivity and planetary health.

INTRODUCTION

Since the Industrial Revolution (~1850 CE), the Earth has undergone continuous warming[1] profoundly affecting the atmosphere, hydrosphere, lithosphere, and biosphere.[2] This change has intensified three interconnected global challenges—food security, environmental sustainability, and soil health—each of which occurs independently or often simultaneously.

(1) The food security challenge. Global food demand is projected to increase by 35%–56% between 2010 and 2050, aiming to meet nutritional needs and alleviate hunger. However, the risk of food insecurity varies widely during this period, ranging from −91% to +8%.[3] The pressure on arable land is increasing, particularly in densely populated regions such as China, India, and many African nations.[4] Rapid urbanization, industrial expansion, and ongoing land degradation continue to reduce cultivable land,[5] further threatening food production.[6] Converting carbon-rich grasslands and forests into croplands results in significant carbon losses[7] and compromises agrifood system resilience.[8] Global disruptions—including the COVID-19 pandemic, geopolitical conflicts, and restrictive trade policies—have also weakened supply chains[9] and increased global food insecurity,[10] underscoring the need to address the imbalance between food supply and demand.
(2) The environmental sustainability challenge. Since the Green Revolution of the 1950s–1960s, agriculture has increasingly relied on synthetic fertilizers,[11] pesticides,[12] and agrofuels.[13] Synthetic nitrogen fertilizers in particular are a significant source of nitrous oxide (N_2O),[14] a potent and long-lived greenhouse gas (GHG). Over the past 40 years, nitrogen inputs to croplands have increased N_2O emissions by approximately 30%,[15] contributing to rising atmospheric GHG concentrations.[16] Globally, food systems emit about 20 Gt CO_2 equiv year^{-1}, about 35% of total GHG emissions,[17] with agricultural production accounting for about half of all non-CO_2 emissions between 1980 and 2016.[15] For instance, wheat—a staple food crop—will likely require significant nitrogen inputs to realize more than 50% of its yield potential in a warming climate,[11] inevitably increasing environmental burdens and highlighting the urgent need to reduce GHG emissions and work toward net-zero agriculture to help reverse climate change.[18]
(3) The soil health challenge. Many unsustainable farming practices have severely degraded soil health.[19] Conventional tillage disrupts soil organic carbon (SOC) accumulation and mineralization, thereby weakening soil structure and fertility.[20] Frequent soil disturbance destroys soil aggregates, reducing carbon stability,[21] while continuous monoculture disrupts microbial communities and biodiversity.[22] Excessive

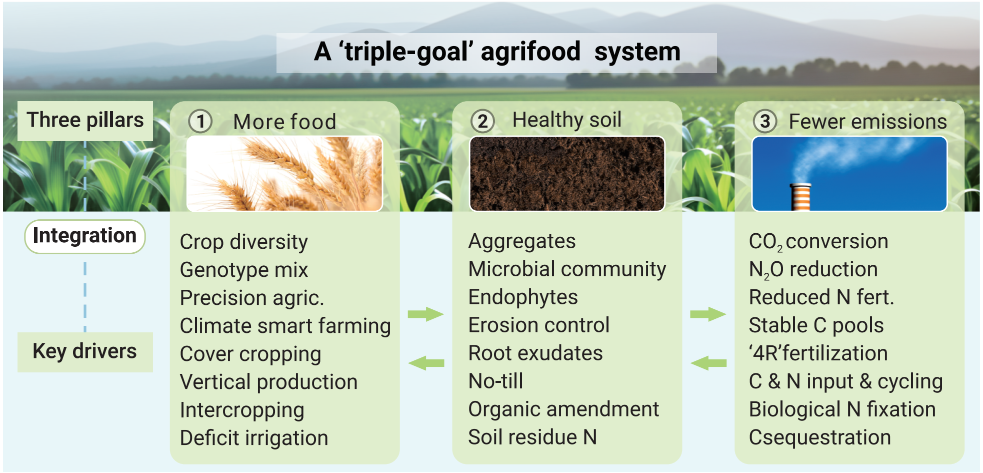

Figure 1. Integration of the three pillars—more food, healthier soils, and fewer emissions—within the triple-goal agrifood framework Each pillar is supported by key drivers. (A) More food through innovative and sustainable practices such as alley cropping, intercropping, genotype diversification, deficit irrigation, cover cropping, legume-based rotations, smart farming, precision agriculture, and vertical farming. (B) Healthier soils through strategies including increased carbon inputs, stable carbon pool formation, soil amendments, reduced or no-till practices, enhanced carbon and nitrogen cycling, improved soil aggregation, stimulation of root exudation, and promotion of endophyte activity. (C) Fewer emissions through enhanced carbon sequestration, reduced or no-till practices, optimized fertilization, improved residue N management, 4R fertilization strategies,[19] erosion control, and management practices to reduce N_2O emissions.

use of synthetic agrochemicals further deteriorates the chemical and biological integrity of soil,[23] leading to soil salinization,[24] acidification,[25] and nutrient imbalances[26] as well as water pollution[27] and habitat destruction.[28] These factors have negatively affected soil health, while crop yields dependent on nitrogen inputs have stagnated[29]—or even declined—in some regions.[30] Although countries like China and India have achieved substantial yield gains in recent decades, those improvements have often come at the expense of soil health.[31] Globally, an estimated 24 billion tons of fertile soil are lost annually, and over 90% of the Earth's land may be degraded by 2050 if current trends continue.[32]

To address these interlinked challenges—feeding a growing population, reducing environmental impacts, and restoring degraded soils—we introduce a "triple-goal agrifood production framework" (hereafter called the triple-goal framework). This integrated, multi-factor approach, synthesizing improved and emerging agricultural practices, is built on three foundational pillars, each supported by key drivers (Figure 1). The triple-goal framework features the following: (1) enhancing system resilience by integrating existing and novel technologies that improve plant-soil-microbe-environment interactions; (2) promoting carbon source-to-sink strategies that boost soil carbon sequestration and support global carbon cycling; (3) stimulating biological processes—including enzymatic and microbial activities—to enhance water and nutrient use efficiency, thereby improving soil biochemical properties; and (4) reducing reliance on synthetic nitrogen by leveraging biological nitrogen fixation (BNF), thereby decreasing nitrogen fertilizer inputs and lowering N_2O emissions. The triple-goal framework is based on a comprehensive synthesis of findings from 104 individual meta-analyses (Table S1), incorporating studies from countries such as Australia, Canada, China, the United States, the United Kingdom, European Union (EU) member states, and others worldwide.

Second-order meta-analysis

In the study, we employed a second-order meta-analysis (SOMA)[33]—similar to the approach used by Beillouin et al.,[23] Xu et al.,[34] and Ascenzi et al.[35]—to synthesize findings across multiple, individual first-order meta-analyses. We defined our target subject area and identified 104 relevant first-order meta-analyses, collectively encompassing 39,162 studies (or experiments) and 300,139 paired comparisons between target treatments and the control groups (Table S1). These articles were selected based on predefined criteria and a structured selection process (Table S2). The geographic distribution of study sites is shown in the global map (Figure S1). The rationale for adopting SOMA in this study, along with its robustness, is provided in the supplemental information.

From the selected 104 meta-analyses, we extracted all effect sizes (e.g., mean differences, odds ratios, Cohen's d, Hedges' g, and Pearson correlation r), their variances (standard errors and confidence intervals), and the number of primary studies and observations. To ensure consistency in the SOMA metrics, we converted all the effect sizes to Hedges' g (a bias-corrected standardized mean difference) using the following formulas:

$$g = \left(1 - \frac{3}{4 * df - 1}\right) \times d \quad \text{(Equation 1)}$$

$$d = \frac{2r_{xy}}{\sqrt{1 - {r_{xy}}^2}} \quad \text{(Equation 2)}$$

where g is Hedges' g, d is Cohen's d, and r is the Pearson $x * y$ correlation coefficient). We employed a random-effects model of comprehensive meta-analysis (CMA)[36] and calculated the summary effect size and its variance using the following formulas:

$$M = \frac{\sum_{i-1}^{k} W_i Y_i}{\sum_{i-1}^{k} W_i} \quad \text{(Equation 3)}$$

$$V_M = \frac{1}{\sum_{i-1}^{k} W_i} \quad \text{(Equation 4)}$$

where M is the summary effect size, V_M is the variance of the summary effect, W_i is the weight for study i, which was computed by the inverse of the sum of the within-study variance for study i and between-study variance, and Y_i is the effect size for study i. We retained independent effect sizes and weighted them by the inverse of their variance, as recommended by Sanchez-Meca and Marín-Martínez,[37] to reduce the influence of lower-quality meta-analyses. Furthermore, we quantified the heterogeneity of true effect sizes using CMA's built-in statistics, including Q value, T^2, and I^2. The Q statistic and its p value test the null hypothesis that the true effect sizes are consistent across studies. $T2$ is the estimate of the variance of the true effects, and I^2 is an index indicating the percentage of variability that reflects the heterogeneity of true effect sizes.

The CMA calculated the Q statistic and I^2 to assess heterogeneity and the significance of τ^2:

$$I^2 = \frac{\tau^2}{\tau^2 + \overline{\overline{\nabla}}} \times 100\% \quad \text{(Equation 5)}$$

$$Q = \sum w_j \left(g_j - \mu\right)^2 \quad \text{(Equation 6)}$$

where $\overline{\overline{\nabla}}$ is the average within the meta-analysis variance, and w_j is the inverse variance weight.

From the SOMA modeling, we identified relevant drivers from a list of predefined soil- and crop-related anthropogenic activities. We then categorized the effect sizes for 6–8 key drivers across the three goal areas: increased food production, healthier soils, and reduced emissions. More detailed descriptions of

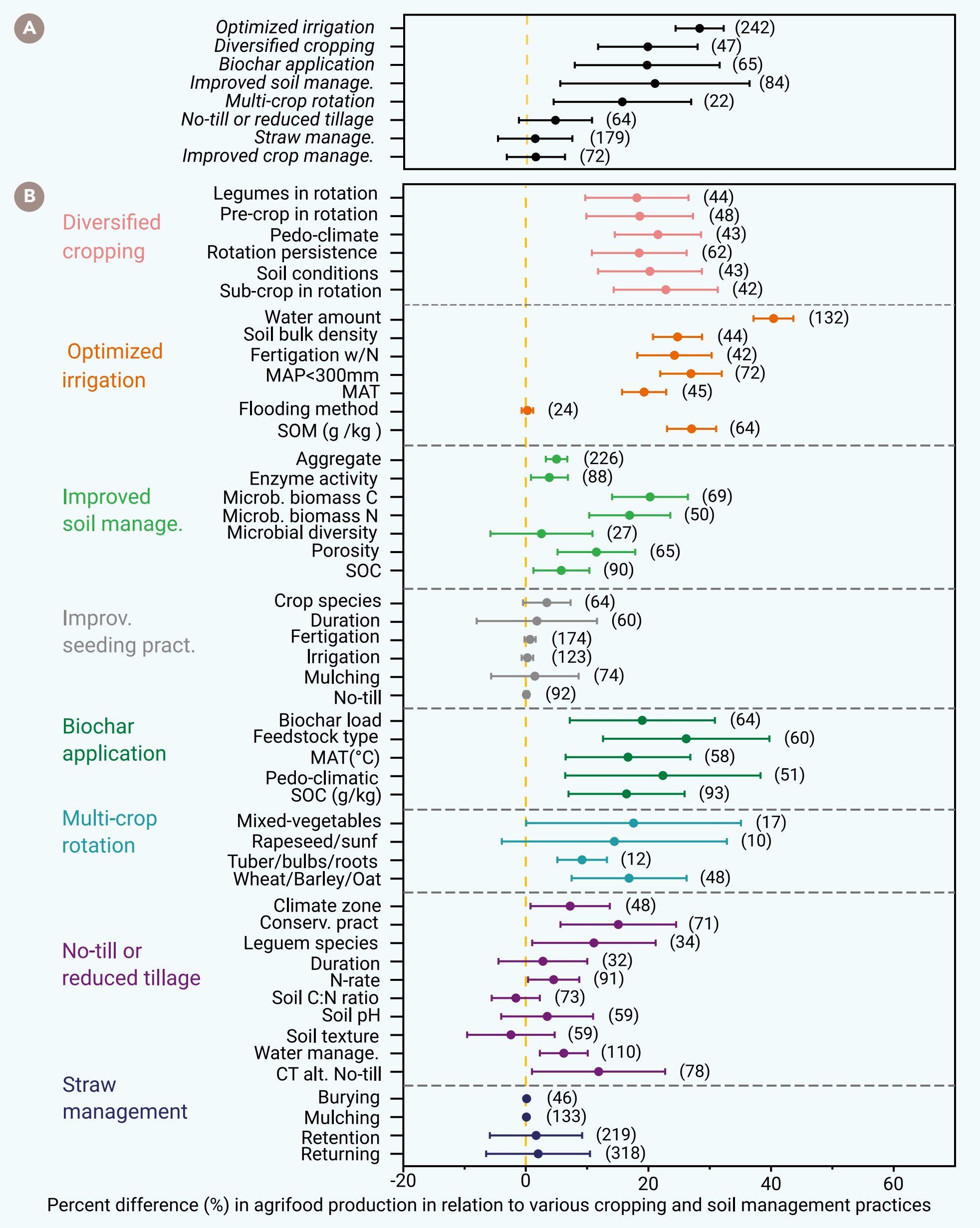

Figure 2. The triple-goal framework integrates established and emerging farming practices to maximize agrifood productivity and stability (A) Results from the SOMA indicate that integrated farming approaches significantly increase crop yields compared to conventional practices. The main contributors to yield gains are optimized irrigation (28.3% increase, *n* = 60 first-order meta-analyses), diversified cropping systems (19.6%, *n* = 47), organic amendments (19.4%, *n* = 65), and improved soil management (18.8%, *n* = 84). (B) Each of these key drivers comprises a range of agronomic practices, leading to varying effects on crop yields. While multi-crop rotation, reduced or no tilling, crop seeding practices, and straw management also improved yields, they generally had smaller effects.

the SOMA methodology, statistical models, publication bias, and limitations are provided in the supplemental information.

The boundaries

The triple-goal framework focuses exclusively on staple and vegetable crops, addressing three key pillars simultaneously—more food, healthier soils, and reduced emissions (Figure 1). Other food sectors, such as fisheries, livestock, synthetic foods, and agroforestry, fall outside the scope of this study. Although these sectors may offer income opportunities for smallholder farmers in Africa,[38] they are beyond the objectives of this analysis. Similarly, while many publications explore soil- and crop-specific agronomic practices for particular cropping systems, consolidating such practices by cropping system was not the aim of this work.

PILLAR 1: MORE FOOD

Core strategies for boosting agrifood production

The triple-goal framework integrates innovative, proven strategies to enhance agrifood productivity, including optimized irrigation, diversified cropping systems, biochar application, and improved soil and crop management (Figure 2). The SOMA showed that optimized irrigation increased agrifood production by 28.3% (*n* = 60 first-order meta-analyses), followed by diversified cropping systems by 19.6% (*n* = 47), organic amendments like biochar by 19.4% (*n* = 65), and improved soil management by 18.8% (*n* = 84), with each effect size weighted by the number of contributing studies or experiments. Multi-crop rotation also increased crop yield by 14.7%, whereas reduced or no-till practices and straw management had comparatively smaller gains. Returning crop residue to the soil as compost or biochar promotes microbial activity, which improves soil nitrogen and boosts production.[39,40] Structural equation modeling further supports the notion that productivity gains are strongly linked to the total nitrogen supplied and nitrogen use efficiency (NUE), which, in turn, are associated with plant nitrogen uptake and soil nitrogen accumulation (Figure S2). However, the effects of biochar on soil nutrients and crop production vary widely depending on soil biochemical properties (e.g., pH and N status),[41] crop species,[42] biochar properties,[43,44] and application methods and rates.[44,45] In the short term (≤5 years), crop yields following biochar application can fluctuate due to interannual variability in temperature and precipitation, although such variability tends to diminish over time.[46]

Traditional agrifood production systems, which rely on monoculture in wheat (*Triticum aestivum* L.), rice (*Oryza sativa* L.), maize (*Zea mays* L.), canola (*Brassica juncea* L.), and other staple crops, face limitations due to socioeconomic barriers and saturated markets focused on caloric and oil products. In contrast, diversified cropping systems—where crops with contrasting morphological, physiological, or biochemical traits are grown together—offer a promising alternative. Diversification may occur across time (e.g., crop rotations), space (e.g., intercropping), or both. For example, southern China supports intensive double or triple cropping due to its favorable climate; the EU commonly uses cover crop-cash crop rotations, and the semiarid northwestern plains of India practice cereal-legume intercropping.[47] These locally adapted approaches can improve food security,[48] enhance profitability, and reduce nitrogen-induced GHG emissions.[49]

Intercropping systems combining forage, grain, oilseed, and legumes in strip,[50] alley,[51] or relay[52] formats improve both productivity and resource use efficiency.[53] One meta-analysis of 88 studies found that maize-soybean (*Glycine max* L.) intercropping improved nitrogen and phosphorus use efficiencies compared to monoculture.[54] Another meta-analysis (226 experiments and 934 observations) found that yield gains from intercropping were equivalent to a 19% land saving compared to sole cropping.[55] These benefits grow with continued use over time due to improved soil fertility[56] driven by enhanced root exudates and nitrogen fixation by legumes.[57] Many other soil and crop management practices have also demonstrated significant advantages over monoculture in supporting the triple-goal framework (Table S3).

www.the-innovation.org

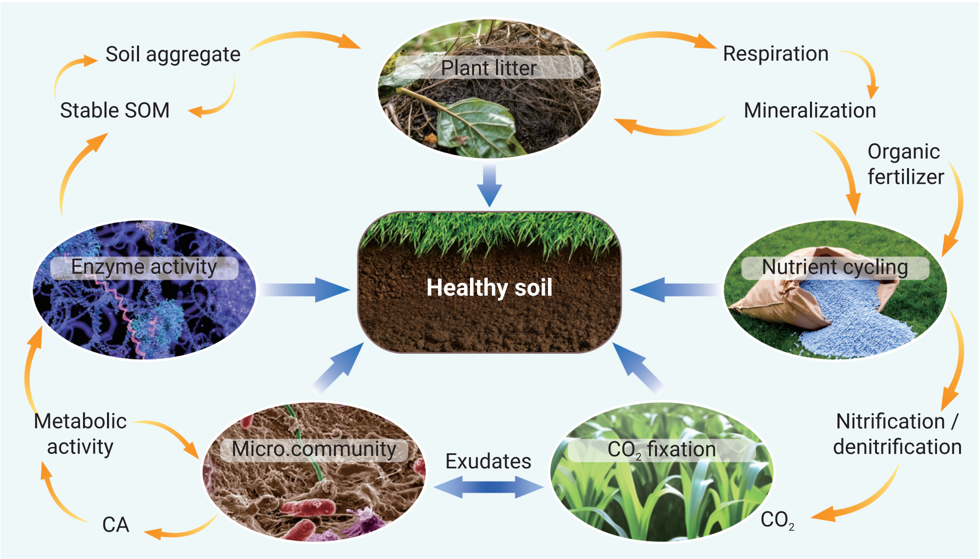

Figure 3. A healthy soil system involves complex metabolic pathways, nutrient transfer and cycling, and dynamic enzymatic and microbial activities Continuous inputs of plant litter and organic fertilizers contribute to maintaining stable soil organic matter (SOM) and improving soil structure through enhanced aggregation, which physically protects carbon pools from degradation and promotes microbial growth and activity. The coordination of (de) nitrification metabolic activities and processes involving in soil respiration and mineralization, driven by enzyme activities and root exudates, plays a key role in nutrient cycling. Optimized soil and crop management strategies can enhance soil health under favorable soil and climatic conditions.

System resilience: Insurance for agrifood security

Agrifood production systems are highly vulnerable to disruptions and shocks caused by geopolitical crises (e.g., the Russia-Ukraine war), public health emergencies (e.g., the COVID-19 pandemic), and natural disasters (e.g., severe droughts), which can significantly impact supply chains at local to global scales.[58] The triple-goal framework seeks to enhance production system resilience, enabling it to tolerate moderate abiotic and biotic stresses while ensuring rapid recovery once disruptions subside.

Diverse cropping systems play a key role in this resilience by disrupting host plant-pest species-environment relationships, thereby reducing the survival of pathogens with narrow host ranges and short life cycles.[59,60] Spatiotemporal crop diversity hinders the establishment of host-favoring fungal microbiomes.[61] Host plants influence microbiome composition through the selective allocation of resources to multiple symbionts,[62] which can further limit pathogen resistance. These practices also help address the growing challenge of pesticide resistance, particularly in regions where excessive pesticide use became widespread following the Green Revolution. Well-designed, diversified systems that incorporate crops with diverse growth habits, life cycles, and morphologies can effectively suppress pest populations. Rotating between cool- and warm-season crops, annuals and perennials, and monocots and dicots disrupts pest life cycles and reduces their persistence.[63] Additionally, using multiple modes of pesticide control (e.g., herbicidal and non-herbicidal strategies) can delay resistance development, supporting long-term crop health.[64] A resilient agrifood production system must strike a balance between production and risk mitigation.[65] One key approach is reducing synthetic nitrogen fertilizer use, a major contributor to N_2O emissions and the nitrogen-induced carbon footprint.[66] Applying organic fertilizers, integrating a broader array of nutrients, and fostering plant-microbe compatibility can reduce N_2O emissions while maintaining or improving productivity; these measures contribute to a more robust and lower-risk agrifood system.

Agrifood challenges following the Green Revolution

A significant challenge facing agrifood systems in the 21st century is the persistent issue of nutritional inequality. According to the Food and Agriculture Organization of the United Nations (FAO) Food Security and Nutrition Report, more than 720 million people were undernourished following the COVID-19 pandemic—an increase of more than 150 million since 2019.[67] Ongoing global uncertainties have further disrupted food supply chains, triggering significant inflation in commodity prices. Countries heavily reliant on agricultural imports, such as those in the Middle East and North Africa, have been particularly affected.[68] Compounding these issues, extreme weather events—such as droughts and floods—continue to place additional pressure on already vulnerable agrifood systems.

While the Green Revolution significantly boosted calorie production—particularly from rice, wheat, and maize—it also contributed to rising nutrient deficiencies in developing nations and increasing overweight and obesity rates in the developed world.[69] Malnutrition remains a widespread public health challenge in sub-Saharan Africa,[70] where the Green Revolution had a limited impact on marginal lands.[71] In Southeast Asia, smallholder farms gained few benefits due to inequitable land ownership, unaffordable inputs, and policies that marginalized small-scale producers.[30,72]

In Africa, abundant arable land and underdeveloped market infrastructure hindered the effectiveness of the Green Revolution.[71] A shift toward regionally adapted staple crops such as millet (*Cenchrus americanus* L.), sorghum (*Sorghum bicolor* L.), and cassava (*Manihot esculenta* L.) could offer greater nutritional benefits.[73] Furthermore, the social aspects of agricultural development were often overlooked in many developing countries during the Green Revolution,[74] leading to class and gender disparities.[75] Women-headed households, in particular, have faced lower crop yields and incomes, leaving them more vulnerable to climate change and economic shocks. Addressing these gender-specific vulnerabilities requires improving women's access to markets, promoting labor-saving technologies, and supporting women's organizations.[73] We advocate for coordinated action by governments, international organizations, and local communities to address the legacy shortcomings of the Green Revolution. A sustainable transformation of global agrifood systems must prioritize social equity, ensuring that smallholder and marginalized farmers have fair access to agricultural innovations and resources.[76]

PILLAR 2: HEALTHIER SOIL

Soil health, defined by the synergistic interaction of biological, physical, and chemical properties,[19] is fundamental for sustaining long-term agrifood productivity. Soils host an astonishing abundance and diversity of life, including earthworms, nematodes, mammals, insects, and microorganisms.[77] A single gram of soil can contain up to 10^{11} billion bacteria,[78] with soils home to approximately 59% of Earth's species, making them the most diverse habitat on the planet.[79] Recent advances in DNA sequencing and metagenomics have deepened our understanding of soil microbial communities, which are key drivers of essential ecological functions. These microbiomes participate in SOC decomposition by releasing hormones and chemical compounds, helping store around 1,325 Pg organic carbon within the top 100 mm of soil.[80] The balance between carbon released into the atmosphere through respiration and carbon stored in the soil through sequestration largely determines the size of soil carbon pools. Even small shifts in this balance can have a significant impact on overall carbon dynamics.[81]

Within this context, our triple-goal framework includes several strategies to optimize soil health (Figure 3). These strategies focus on balancing plant litter input, soil aggregates, and soil organic matter stability, regulating carbon loss through respiration and mineralization, managing organic fertilizer input and nutrient cycling, controlling (de)nitrification and CO_2 fixation, and fostering microbial community diversity and metabolic activity. Exudates and enzyme activities are crucial intermediaries that link microbial communities to soil structure.

Our SOMA of original meta-analyses indicates that soil bio-physiochemical properties and anthropogenic activities influence soil health (Figure 4). Increased soil infiltration enhances the Cornell Soil Health Index[31] by 127% on average (n = 61 meta-analysis studies). Alternating conventional tillage with

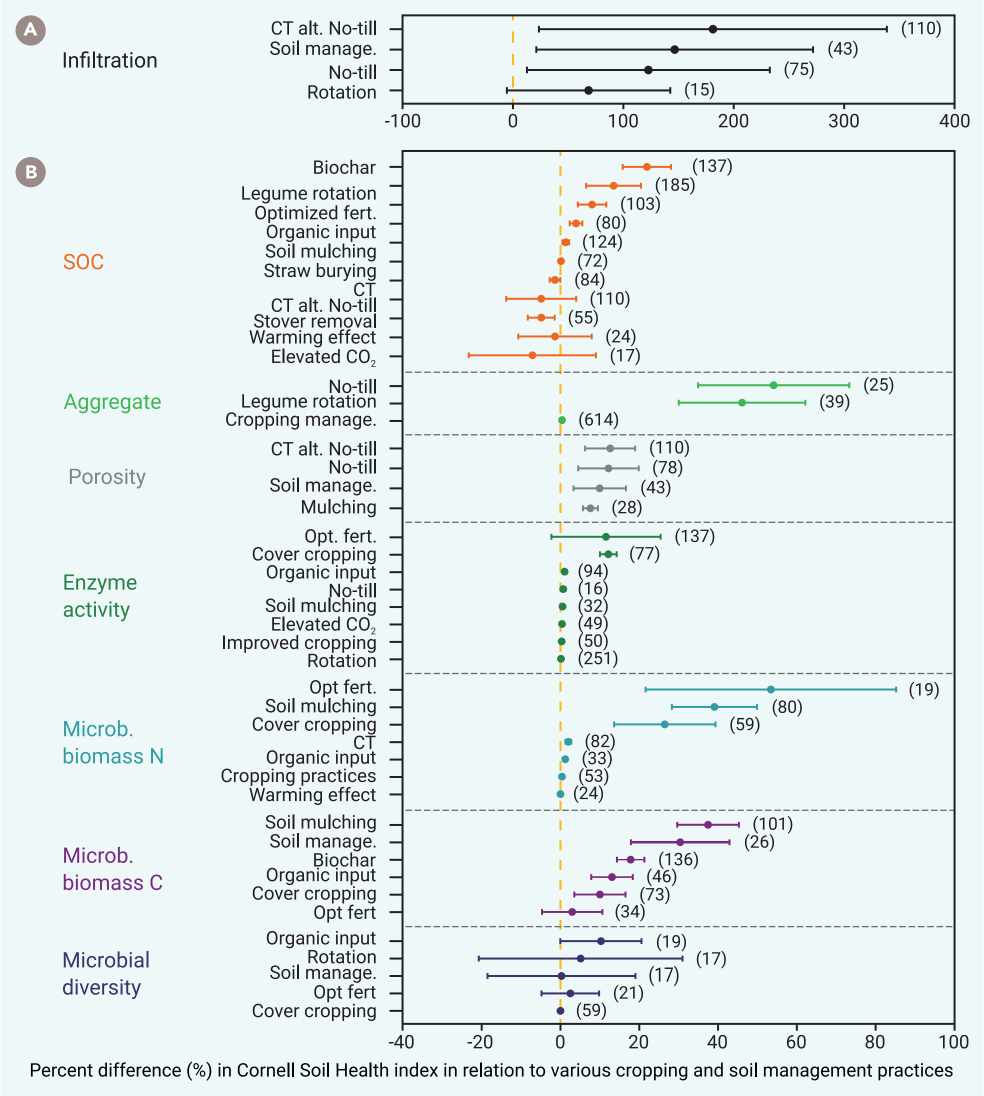

Figure 4. Key driving factors impacting soil health (A) The SOMA revealed that soil infiltration is the most critical driver impacting soil health, as indicated by the Cornel Soil Health Index. Soil infiltration is closely related to tillage, crop rotation, and other soil management practices. (B) Various anthropogenic activities impact soil health by altering soil physiochemical and biological properties, including SOC (SOC), aggregate stability, soil porosity, enzymatic activity, microbial biomass carbon (MBC), microbial biomass nitrogen (MBN), and microbial diversity. Each of these seven soil properties is influenced by different soil- and crop-related practices.

no-till methods contributes most to improved soil infiltration, followed by soil management and crop rotations (Figure 4A). Other soil properties that positively impact the Cornell Soil Health Index include soil aggregates (45%, n = 69), microbial biomass carbon (MBC) (20%, n = 69), microbial biomass nitrogen (MBN) (17%, n = 50), and soil porosity (11%, n = 65) (Figure 4B). Other soil factors, such as SOC, enzymatic activity, and microbial diversity, all showed positive but narrower effects on the soil health index (3.1%–5.6%). Key anthropogenic activities impacting soil health include crop cultivation, optimized fertilization and irrigation (e.g., fertigation), and improved cropping practices.

Structural equation modeling revealed complex relationships between soil properties, both positive and negative (Figure 5). Strong positive correlations exist between enzymic activity and SOC, MBC and MBN, MBC and microbial richness, MBN and SOC, MBC and porosity, and MBC and aggregation. These relationships highlight the importance of refining cropping and soil management practices to improve soil health, with the strength of these correlations varying according to local conditions.

Managing soil carbon to enhance soil health

Stable SOC within aggregates is a significant nutrient reservoir that enhances the soil's buffering capacity.[82] Numerous anthropogenic activities influence SOC accumulation, transportation, and decomposition, thereby affecting the size and stability of SOC pools. A meta-analysis of 269 studies, encompassing 2,035 observations, showed that adding organic materials, such as biochar, manure, and crop straw, increased aggregate-protected carbon by 21%–34% and aggregate stability by 19%–23%, thereby reducing the decomposability of aggregate-associated organic carbon.[83] Applying nature-based organic amendments to soils in arid and semiarid climates lowered soil temperatures, protected soil aggregates, and enhanced stable carbon storage. Improved cropping systems—such as cover cropping and agroforestry—also substantially increased stable SOC stocks. A global meta-analysis of 434 paired observations[84] found that cover cropping increased SOC stocks at 60% of the study sites, particularly where initial SOC concentrations were below 11.6 g kg^{-1}. These findings highlight that anthropogenic management is crucial for enhancing SOC stocks, with aggregate-protected carbon pools being key repositories for long-term carbon sequestration and improved soil health.

SOC plays a crucial role in shaping the soil's physicochemical properties. While mineralogy and texture largely determine the baseline SOC content,[85] agricultural practices can significantly modify SOC levels, influencing bulk density, cation exchange capacity (CEC), and the soil's potential for carbon sequestration. For instance, increasing organic matter inputs enhances SOC concentrations by forming stable complexes with soil minerals. Higher SOC levels improve bulk density and CEC, enhancing soil structure and overall functionality. The soil carbon-to-nitrogen ratio influences carbon assimilation and emissions due to the positive association between SOC decomposability and CO_2 cycling.[86] In conjunction with local climate conditions, SOC provides critical habitats for microbial biodiversity, which supports nutrient cycling, water infiltration, and the breakdown of contaminants.

In agrifood systems, SOC accumulation is driven by inputs from root exudates, plant residues, and microbial necromass. A net sequestration rate of 2.1 Mg C ha^{-1} $year^{-1}$ is required to counterbalance global soil carbon losses. Projections suggest that annual inputs of 5.1 Mg C ha^{-1} could raise SOC stocks to 55 Mg C ha^{-1} by 2050.[87] However, SOC stability is often undermined by the priming effect, whereby fresh organic inputs stimulate native SOC mineralization. Meta-analyses indicate that exogenous carbon amendments can increase native SOC decomposition by up to 61% in croplands,[88] with priming intensity governed by SOM recalcitrance and mineral-organic interactions.[89]

The soil microbiota plays a central role in carbon cycling, performing key metabolic functions. Anabolic processes lead to the synthesis of complex organic compounds, while catabolic processes mineralize SOC, releasing energy-rich byproducts such as pyruvate and ethanol, along with secondary metabolites that influence soil food web dynamics.[90] Microbial residues represent a diverse and significant fraction of stable SOC, potentially up to 50%,[91] forming persistent organic pools and highlighting microbial metabolites as accumulators critical to long-term carbon storage and microbially derived carbon stability. Globally, soils release an estimated 75–100 Pg CO_2 annually through respiration,[92] reflecting the diverse metabolic pathways of different decomposers: bacteria dominate plant residue breakdown,[93] archaea drive methanogenesis in anoxic environments, and fungi specialize in degrading recalcitrant compounds such as lignin and cellulose.[91] Some specialized soil fungi can also decompose the carcasses of insects and earthworms. Additionally, protozoa influence

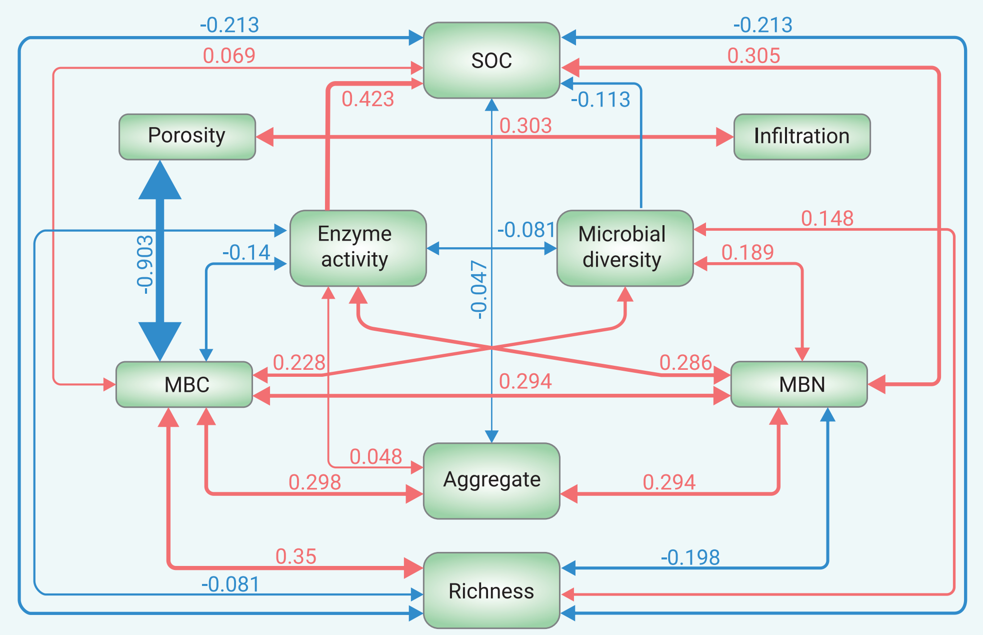

Figure 5. Structural equation modeling demonstrates that many soil properties exhibit significant interactions Notably, there are highly significant, positive relationships between enzyme activity and soil organic carbon (SOC), MBC and MBN, MBC and microbial richness, MBN and SOC, MBN and porosity, MBN and infiltration, and MBN and enzymatic activities. Less important factors to the triple-goal framework were excluded based on their correlation coefficients (indicated by the numbers beside the corresponding lines).

carbon cycling by selectively grazing on microbial populations, thereby modulating decomposition dynamics.[92]

Promoting microbial community functioning to enhance soil health

The increasing recognition of the critical role of soil biodiversity in ecosystem functioning has intensified efforts to develop strategies that assess the contributions of distinct soil biological groups. These strategies are vital for conserving habitats and sustaining soil ecosystem health. Soil microbial communities exhibit uneven spatial and temporal distributions,[94] with their biogeochemical impacts varying significantly at global, regional, and field scales. At broader scales, climate, parent material, and topography shape the underlying soil physicochemical and structural properties, which govern microbial community composition and functional potential.[77] Even within a single soil type, microscale variations in structure and chemistry—such as differences in pore size, aggregate distribution, and root architecture—can increase microbial diversity by affecting oxygen levels, water availability, and nutrient dynamics.[95] Microbial populations are primarily concentrated in the rhizosphere, where root exudates and decaying roots provide readily available carbon sources.[96] This spatial variability renders microbial communities highly responsive to land use and management practices. Temporal drivers, such as seasonal cycles and microclimate fluctuations, further influence microbial abundance, composition, and diversity.

Soil microbes play a vital role in decomposing plant biomass into SOM, stabilizing it by forming organo-mineral complexes,[97] sequestering it within soil aggregates,[98] or mineralizing it and releasing CO_2 into the atmosphere.[92] As key regulators of carbon cycling, soil microbes play a central role in maintaining ecosystem services, particularly in agricultural systems. Unlike natural ecosystems, where microbial communities are shaped predominantly by inherent soil properties, climate, and vegetation, managed agricultural systems allow for deliberate interventions that influence microbial dynamics. Increasing soil carbon inputs not only compensates for carbon losses due to harvesting but also contributes to climate change mitigation by promoting the formation of stable carbon pools and enhancing microbial resilience.[99] Strategic adjustments to management practices can result in long-term improvements in soil properties,[100] reduce anthropogenic carbon emissions,[101] and strengthen soil health and climate resilience. Fostering healthy and diverse microbial communities in soil contributes to more sustainable agriculture and global food security.

Managing nitrogen to enhance soil health

Soil nitrogen availability is governed by three interrelated sources: BNF, organic nitrogen mineralization from decomposing plant residues, and atmospheric nitrogen deposition. Among these, BNF can contribute up to 70% of the aboveground plant nitrogen in legume-dominated systems, highlighting its potential for reducing the reliance on synthetic nitrogen fertilizers. The triple-goal framework encourages the use of nitrogen-fixing microbial technologies, such as *Rhizobium* and arbuscular mycorrhizal (AM) fungal inoculants, to enhance nodulation and phosphorus uptake, thereby improving symbiotic nitrogen fixation. At the same time, the mineralization of organic nitrogen—regulated by soil moisture and temperature, physicochemical properties, and functional microbial communities—supplies plant-available ammonium (NH_4^+), which can then be nitrified into nitrate (NO_3^-) or denitrified into nitrous oxide (N_2O) and dinitrogen (N_2).[102] Specialized microbial consortia mediate these transformations, facilitating the flow of nitrogen between organic matter (e.g., crop residues and manure) and plant-accessible forms.

The triple-goal framework promotes (1) precision management practices, such as the 4R approach to fertilization[19] (applying the right source, at the correct rate, at the right time, and in the right place), to maximize plant nitrogen uptake and minimize excess inorganic nitrogen accumulation; (2) crop residue retention and the use of organic amendments to stabilize nitrogen pools;; and (3) innovative interventions such as biochar, nitrification inhibitors, and slow-release fertilizers to modulate key biochemical processes and reduce N_2O emissions. Importantly, the effectiveness of these strategies is highly context dependent, shaped by controllable factors (e.g., crop genotype and tillage) and uncontrollable variables (e.g. and climatic extremes).[102] Therefore, integrated nitrogen management must remain adaptive and site specific to support soil health and agricultural sustainability.

Managing the microenvironment to enhance soil health

Soil microenvironments that support microbial activity are fundamental to driving nutrient cycling and detoxification processes. Extracellular enzymes play a central role in these functions, playing key roles in nutrient turnover[103] and SOM mineralization,[104] and are well-recognized soil health indicators. Their activity—directly linked to substrate availability—reflects microbial metabolic potential and serves as a biomarker of soil functionality.[19] Beyond decomposing SOM to fuel microbial biomass, these enzymes mediate nutrient transformations, break down contaminants such as heavy metals and microplastics, and suppress soil-borne pathogens. Notably, synergistic plant-microbe interactions can enhance crop tolerance to heavy metals, offering a sustainable approach to managing contaminated agroecosystems.[105]

Our triple-goal framework targets key soil management practices to improve microenvironmental conditions and support soil health. Root-derived carbon inputs—such as rhizodeposition, root exudates, and necromass—contribute disproportionately to soil carbon pools compared to aboveground residues. These root inputs are closely linked to microbial activity and enzymatic processes and are considered a critical indicator of soil health.[19] The abundance of the *cbbL* gene, which encodes bacterial Ribulose-1,5-bisphosphate carboxylase/oxygenase, serves as a proxy for the carbon sequestration potential of soil autotrophic microorganisms.[106] Higher *cbbL* gene copy numbers are often observed under conservation tillage compared to conventional tillage due to the favorable microenvironments created by added organic matter.[107] However, responses can vary depending on soil nutrient status and the duration of nutrient

management.[108] In some cases, soil CO_2-fixing genes may not respond positively to soil disturbance or nutrient amendments.[109]

Another focus of the triple-goal framework is reducing anthropogenic disturbances of the soil structure. Limiting agrochemical inputs such as pesticides and herbicides is critical for preserving microbial diversity and function. Additional practices that promote SOC accumulation and maintain microbial habitats include reducing soil compaction through optimized machinery use, retaining plant residues, and incorporating organic amendments. It is also essential to exclude contaminants, including antibiotics,[110] microplastics,[111,112] and heavy metals[113]—especially when using recycled organic amendments[114]—to protect microbial communities and ensure the delivery of ecosystem services.

Legume-based diversification offers a multifunctional strategy for improving soil health (Figure S3). Incorporating legumes into crop rotations supports BNF and system resilience, reducing the need for synthetic nitrogen inputs and associated CO_2 emissions. A meta-analysis of 462 studies (11,768 observations) revealed that legume rotations can increase subsequent crop yields by an average of 20% across diverse pedo-climatic conditions.[115] Regional case studies further demonstrate 7%–13% SOC increases in maize-wheat systems in the Indo-Gangetic Plains[116] and 50%–102% reductions in CO_2 emissions in temperate legume-intercropping systems.[117]

Reduced tillage and crop diversification are complementary practices that work together to enhance soil health. A global meta-analysis (comprising 77 articles and 393 treatments) found that combining no-till practices with crop diversification enhances fungal abundance, improves the fungus-bacterium ratio, and optimizes nutrient cycling. However, such practices must be adapted to local conditions, balancing soil health gains with the need to maintain cereal yields, particularly in regions where food security remains a pressing concern.

Innovative cropping strategies can also promote biodiversity-based production systems. For example, perennial tropical crops like banana (*Musa acuminata*) and coffee (*Coffea arabica*), grown in shaded agroforestry systems, support greater biodiversity,[118] while annual crops such as maize, sugarcane (*Saccharum officinarum* L.), and oil palm (*Elaeis guineensis* L.) tend to diminish when grown in open conditions.[118] In the northern Great Plains of North America, integrating annual legumes into traditional wheat or oilseed monocultures enhances above- and belowground microbial biodiversity.[119] In Asia, applying biochar derived from pyrolyzed carbon feedstocks improves soil sustainability.[120] Selecting appropriate feedstocks and pre-pyrolysis activation methods can enhance biochar's ability to adsorb and immobilize heavy metals, benefiting the remediation of contaminated environments.[121]

Conditioning soils with amendments to enhance soil health

The foundational role of soil health in supporting agrifood productivity and resilience is widely acknowledged. However, tailoring amendment strategies to specific soil conditions is crucial for sustaining long-term soil functionality. Within the "triple-goal" framework, a key focus is on enhancing soil health through the use of organic amendments that improve its physicochemical and biological properties. Directly incorporating organic matter—such as livestock manure (raw or processed), green waste compost, and anaerobic digestate—remains a proven approach to increasing SOM.[122] Emerging circular economy innovations are expanding the range of available amendments, including fishery byproducts repurposed as nutrient-rich fertilizers,[123] insect frass from black soldier fly farming,[124] and human-derived fertilizers (e.g., sanitized sewage sludge and urine).[125] Biochar, a carbon-rich material produced by pyrolysis, also holds promise for conditioning soils by enhancing microbial diversity, stabilizing SOM,[121,] and mitigating soil contamination (e.g., heavy metals) through its strong adsorption capacity.[126] However, biochar's effectiveness depends heavily on the type of feedstock and pyrolysis temperature,[121] and its widespread use is often limited by logistical and financial constraints.[126] Moreover, biochar addition—alone or combined with other organic amendments—can sometimes increase CO_2,[127] N_2O,[128] or CH_4[129] emissions, raising the overall global warming potential.[130]

Organic fertilizers enhance soil structure by improving aggregation, reducing bulk density, and acting as carbon sinks.[131] They contribute beneficial microbial consortia while stimulating native microbial communities. However, their effects on soil health are highly variable, influenced by feedstock source, processing technique, and application rate. Significant challenges associated with organic fertilizers include GHG emissions from incomplete mineralization,[132] the risk of contaminants (e.g., antibiotics and heavy metals),[133] and issues related to labor intensity and inconsistent nutrient release.[132]

Enhanced rock weathering has recently emerged as a novel strategy for carbon sequestration. This practice involves applying crushed silicate or carbonate minerals (e.g., basalt and dolomite) to agricultural soils. It has the potential to sequester an estimated 0.5–2 billion tons of CO_2 annually in major cropping regions.[134] In addition to carbon capture, benefits may include increased soil pH, improved availability of micronutrients (e.g., calcium and magnesium),[135] and higher crop yields in acidic soils.[136] However, the long-term effects of rock weathering amendments on soil microbial communities and SOM dynamics remain unresolved.[134]

PILLAR 3: FEWER EMISSIONS

Mitigating N_2O emissions in the triple-goal framework

Primary food production systems generate 70%–85% of global anthropogenic N_2O emissions—a potent GHG with a global warming potential of 298 times greater than CO_2 over a 100-year atmospheric lifetime that exacerbates climate change and stratospheric ozone depletion.[137] Fertilized croplands are the primary source of N_2O emissions, driven by microbially mediated nitrification ($NH_3 \rightarrow NO_2^- \rightarrow NO_3^-$) and denitrification ($NO_3^- \rightarrow N_2O$ or N_2), processes regulated by key functional genes (e.g., *nirK*, *nirS*, and *nosZ*). Exogenous N inputs elevate *nirK* and *nirS* abundance while suppressing *nosZ*, skewing nitrogen cycling toward increased N_2O production. Reducing the oxidation of NH_4^+ to NO_3^- is critical, as NO_3^- leaching and subsequent denitrification account for 30%–50% of total nitrogen losses.[103] In the "triple-goal" framework, emissions are assessed across on-farm (e.g., fertilizer and pesticide application) and off-farm (e.g., production, transport, and storage of agrochemicals) activities (Figure S4). These emissions are expressed as CO_2 equiv, following Intergovernmental Panel on Climate Change guidelines.[138] Notably, the production of synthetic nitrogen fertilizer contributes 2.8–16.1 kg CO_2 equiv per kg of nitrogen applied.[139]

Anthropogenic interventions play a central role in mitigating N_2O emissions (Figure 6A). Key strategies include cover cropping and integrating legumes into crop rotations. A meta-analysis of 372 studies found that cover crops can reduce N_2O emissions by 18%–30% in soils with moderate carbon ($\sim$20 g kg^{-1}) and nitrogen ($\sim$3 g kg^{-1}) levels.[140] Legume-based rotations, such as those involving chickpea or pea, can cut emissions by 56–65% compared to canola monocultures,[141] although tradeoffs exist between maximizing yields and minimizing nitrogen losses (Figure 6B). Tillage and hydrothermal conditions also influence N_2O emissions, with no tilling increasing emissions by 6%–13% in arid regions but reducing them by 11% in humid[142] or low-C soils (<20 g kg^{-1}).[143] However, these effects tend to diminish over time as the soil structure improves and reduces anaerobic microsites.[144] A meta-analysis of 37 studies found no consistent impact of tillage (up to 40 years) on N_2O emissions, regardless of the tillage method used (e.g., moldboard plow, chisel plow, or double-disk systems).[145]

Crop plants typically absorb only a portion of applied nitrogen fertilizer, with the remainder, particularly mobile NO_3, becoming a substrate for nitrification and denitrification.[103] Globally, NUE—the proportion of fertilizer nitrogen taken up by plants—ranges from 25% to 50% in season, with an additional 5%–20% uptake in the subsequent season.[146] Precision nitrogen management strategies, such as 4R,[19] can significantly enhance NUE using slow-release fertilizers, nitrification inhibitors, and variable-rate application.

Structural equation modeling identifies N_2O as the dominant nitrogen loss pathway, decoupled from runoff and respiration losses (Figure S5). Among mitigation strategies, optimizing BNF offers significant potential (Figure S6). Currently, BNF contributes 1.4 Mt of nitrogen annually, a figure projected to increase by 56% by 2100 under elevated CO_2 conditions.[147] Key strategies to enhance BNF include (1) legume and non-legume intercropping (e.g., *Arachis hypogaea*-maize rotation), which promotes rhizosphere metabolites (flavonoids and coumarins), nodulation, and nitrogen fixation[148]; (2) microbial synergies, such as co-inoculation with AM fungi and *Rhizobium*, which improves phosphorus uptake[57] and alleviates nitrogen limitations[149]; and (3) adaptation to soil-climate interactions, as optimal BNF occurs at $\sim$25°C. Climate change is

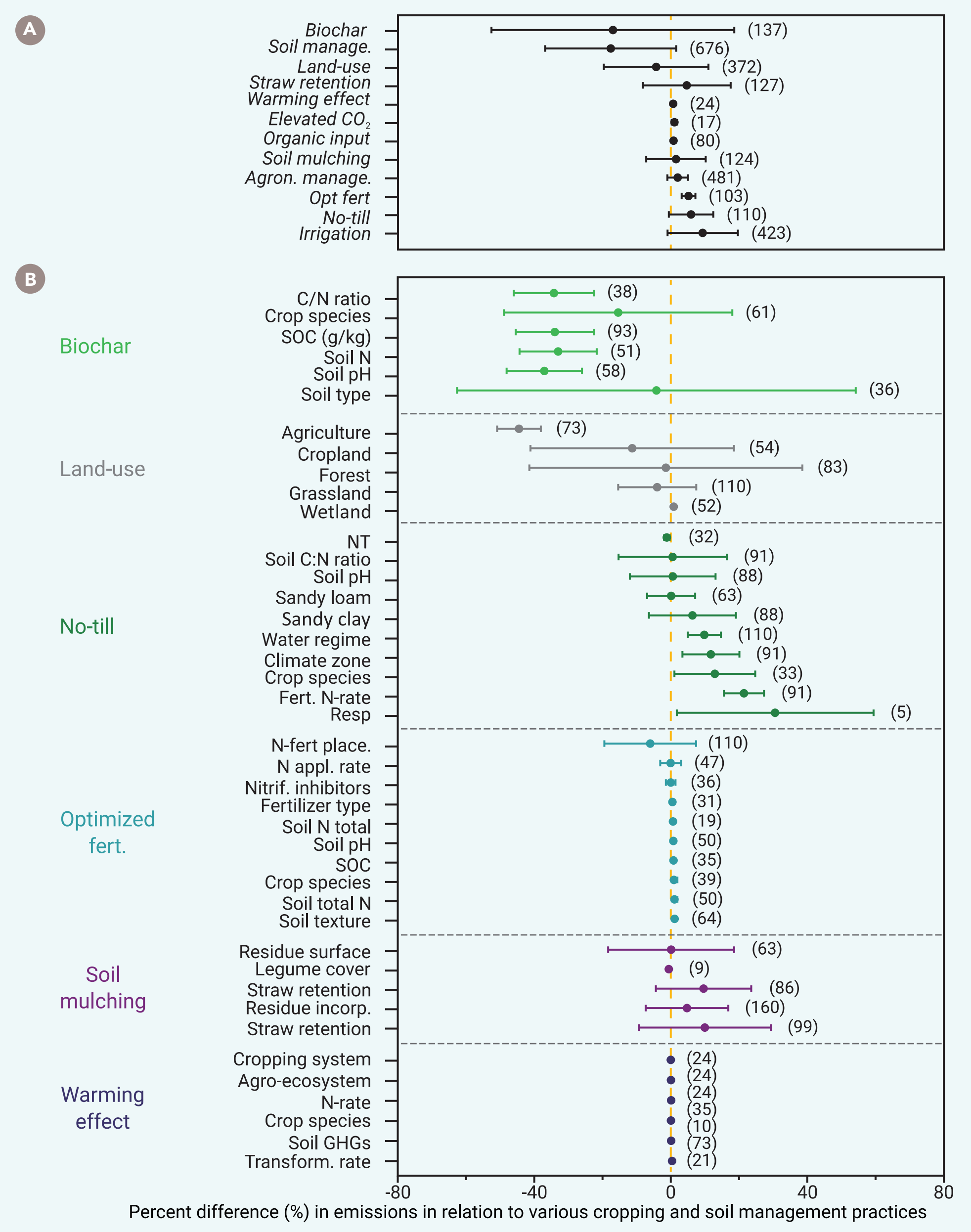

Figure 6. Key drivers regulating N_2O emissions in agrifood production systems The triple-goal agrifood production system adopts a multidisciplinary approach to simultaneously boost food production, reduce GHG emissions, and improve soil health. This approach system mitigates nitrogen-induced emissions through practices such as (A) biochar application, improved soil management, straw retention, land-use optimization, and reduced tillage during cropping. However, many current anthropogenic activities aimed primarily at increasing crop yields—such as (B) intensive tillage, soil mulching, and climate change-related warming—can exacerbate nitrogen losses.

expected to enhance BNF at higher latitudes (+50%) while reducing efficacy in tropical regions (−50%).[150] Non-symbiotic BNF (e.g., in crops like sugarcane and tobacco) play a minimal role in global nitrogen inputs due to low carbon use efficiency (0.012–0.02 g N per g C).[151]

Exploring CO_2 fertilization by promoting CO_2 biotransformation

Over the past 50 years, the seasonal amplitude of atmospheric CO_2 has increased in the Northern Hemisphere,[152,153] intensifying a "fertilization effect" that enhances photosynthesis and promotes the conversion of CO_2 into plant biomass, which will likely significantly increase carbon capture via vegetation in the coming decades. Within the triple-goal framework, a key focus is on optimizing this biotransformation by leveraging source-sink mechanisms, including leaf area index, canopy architecture, aboveground biomass accumulation, the evaporation-to-transpiration ratio, solar energy interception, and plant respiration. These physiological and structural dynamics could mitigate up to 30% of anthropogenic CO_2 equiv emissions. However, the terrestrial hydrological cycle influences the extent of the CO_2 fertilization effect, impacting the rate of CO_2 conversion. Additionally, enhancing CO_2 biotransformation via the CO_2 fertilization effect could increase soil carbon emissions[153] due to shifts in land-atmosphere carbon fluxes, which are governed mainly by atmospheric CO_2 levels and temperature.

The triple-goal framework proposes several management strategies to enhance carbon source-to-sink biotransformation under rising CO_2 levels: "precision agriculture,"[154]— using data-driven technologies like geographic information systems,[155] remote sensing,[156] and the Internet of Things[157] to optimize resource use, monitor crop performance, and increase productivity with reduced environmental impact, and "vertical farming," incorporating hydroponics, aeroponics, and Light-Emitting Diode lighting in vertically stacked systems[158] to maximize space efficiency and enable continuous urban food production. However, its contribution to global food supply remains limited; "climate-smart agriculture" (CSA)—promotes CSA approaches to support sustainable agrifood systems by improving soil health, crop yields, and resilience to biotic and abiotic stresses while simultaneously reducing GHG emissions.[159] Scaling up CSA requires ongoing investment in capacity building, communication, and farmer engagement. It is key to understanding growers' perceptions and willingness to adopt adaptation and mitigation strategies. Cross-sectoral collaboration is also vital for long-term success. "Automated systems"—integrating technologies such as smart sensors, drones, robotic harvesters, and automated irrigation[160,161] to improve operational efficiency and environmental sustainability across indoor and open-field agriculture; "technological innovation" leverages cutting-edge tools like gene editing (e.g., CRISPR-Cas9[162]) to develop cultivars with improved resource use efficiency, high-throughput sequencing to study soil-root-microbe–environment interactions,[163] remote sensing technologies (Normalized Difference Vegetation Index and Enhanced Vegetation Index) to more precisely map carbon sinks and water availability,[164] and explore biofertilizer options,[165] such as AM fungal inoculants for promoting sustainable development.[166] Introducing N_2O-reducing bacteria in the hyphosphere offers further potential for reducing emissions.[167] Additionally, digital cropping systems can enhance sustainability,[168,169] and the root-associated microbiota plays a vital role in conferring plant resistance to abiotic stresses such as aluminum toxicity and phosphorus deficiency.[105] Despite these advancements, significant disparities in access to innovation persist between wealthy and low-income countries. Addressing this inequity will require broader societal and cultural transformation to ensure that the benefits of advanced emerging technologies are shared globally.

INTEGRATION FOR LOCAL SOLUTIONS

The triple-goal framework integrates novel and improved strategies to optimize the balance between the three pillars: agrifood production, soil health, and GHG emissions (Figure 7). A synthesis of more than 39,000 studies revealed a highly significant positive correlation among the three pillars, with

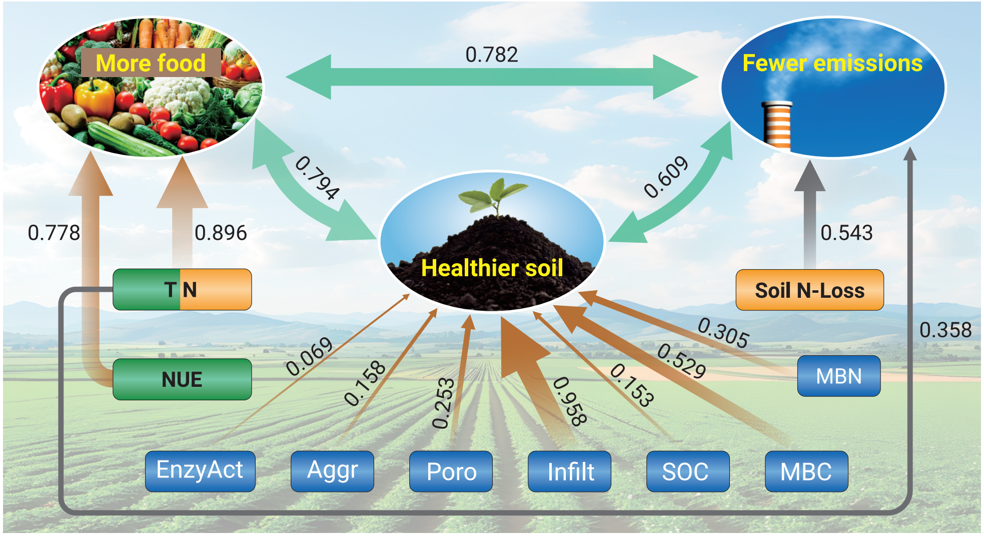

Figure 7. The relationship between food production, soil health, and GHG emissions The three components—food production, soil health, and GHG emissions—are strongly interconnected, with correlation coefficients greater than 0.609. Total soil nitrogen (TN) is the most influential factor in achieving high food production, followed by nitrogen use efficiency (NUE); both are positively associated with food production and emissions. Key soil health indicators include infiltration (Infil), MBC, MBN, and porosity (Poro). Soil enzyme activity (EnzAct), aggregate stability (Aggr), and SOC also contribute positively, though to a lesser extent. Some factors with minimal relevance to the triple-goal framework were excluded. Correlation coefficients are shown alongside the connecting lines.

coefficients exceeding 0.61, indicating that technologies aimed at boosting agrifood yields often result in increased GHG emissions. High crop yields depend primarily on total nitrogen and NUE as key determinants. Notably, total soil N plays a dual role—showing a strong positive correlation with food production (r = 0.90) and a moderate correlation with emissions (r = 0.36). The primary sources of GHG emissions are soil nitrogen losses and changes in total soil nitrogen levels. Soil infiltration, MBC, MBN, porosity, aggregate stability, and enzyme activity are all key contributors to soil health. The observed positive relationship between food production and emissions primarily stems from the addition of fresh carbon to the system through increased inputs of fertilizer and organic amendments. Our SOMA identified the relative influence of over 120 natural and anthropogenic factors affecting these interconnections. These factors are associated with soil physical properties, agronomic practices, and environmental conditions. Effective nitrogen management is crucial for balancing the nexus of food production, soil health, and GHG emissions. However, not all solutions are universally applicable. Some practices that support soil biological functions may hinder other processes. For example, conservation agriculture, residue retention, and no-till methods can help retain SOM in drier regions but may increase soil-borne pathogens in humid regions.[170]

There is no one-size-fits-all solution for enhancing soil health, increasing food production, and reducing emissions across diverse cropping systems. Strategies must be tailored to local contexts. While crop diversification has many benefits, its success depends on regional markets, farm size, farmer incentives, and social and cultural practices. Currently, there is no globally accepted framework for carbon farming, and although agricultural soils have significant potential for climate mitigation, the mechanisms underlying this potential remain poorly understood. Developing cost-effective monitoring tools and performance indicators will help farmers and land managers implement practical, locally adapted strategies aligned with the triple-goal approach. Institutional and government support will enable flexible management that takes into account current social, climatic, and environmental conditions. Coordinated policies and guidelines are necessary across all governance levels to drive progress toward these three interconnected goals. The "triple-goal" framework promotes the integration of innovative and proven practices to develop localized solutions that address the tradeoffs among food production, soil health, and environmental outcomes.

PERSPECTIVE FOR FUTURE GROWTH

Since the Green Revolution, countries have followed diverse development trajectories in building effective agrifood systems. However, the path toward resilience is highly context specific and must be tailored to each nation's unique biophysical and socioeconomic conditions.[171] Climate change is projected to have profound effects on agriculture, underscoring the urgency of adopting climate-resilient food production systems that address both synergies and tradeoffs in mitigation and adaptation.[172] There is a growing need for innovative approaches that prioritize productivity and sustainability to meet global food demands while minimizing environmental degradation.[173] Emerging digital technologies—like sensors, uncrewed aerial vehicles, GPS-guided systems, autonomous monitoring devices, and advanced data analytics—offer promising solutions for improving productivity[174] and resource use efficiency while reducing GHG emissions. Artificial intelligence is revolutionizing agriculture by enabling precise, data-driven input applications that optimize crop growth and reduce waste. However, to fully realize the potential of these technologies, challenges such as high initial investment costs, disparities in technology access, data privacy concerns, and the need for farmer education must be addressed.

Agrifood systems today must strike a delicate balance: feeding a growing global population, adapting to climate change, and mitigating GHG emissions while simultaneously restoring degraded soils. Despite soil health not being included in the United Nation's (UN's) Sustainable Development Goals,[175] it has gained prominence through the "One Health" initiative,[176] which seeks to optimize the health of people, animals, and ecosystems.[177] Current research increasingly emphasizes climate adaptation and crop sensitivity,[178] and the frameworks introduced by the World Bank and FAO under the International Assessment of Agricultural Knowledge, Science, and Technology for Development 2002 offer practical strategies to support these goals. We advocate for a renewed emphasis on conversation agriculture, tailored across biophysical and socioeconomic contexts. This paper's triple-goal agrifood production framework integrates emerging and established technologies to enhance productivity, mitigate N_2O-driven GHG emissions, and promote holistic water-carbon-nutrient cycling, thereby improving soil health. This approach provides a roadmap for decision-makers to develop climate-smart, resource-efficient, and stress-resilient agrifood systems, serving as a potential global model for multifunctional agriculture.

Nonetheless, we acknowledge potential limitations in implementing the triple-goal system. In less developed countries, for example, economic constraints may favor cash cropping over diversified systems, limiting the feasibility of cropping diversification. Regional agricultural conditions may also restrict the adoption of a diverse crop mix, depending on climate, soil type, and market access. Effective policy development requires collaboration among governments, business sectors, and local communities to address barriers to adopting practices such as no-till farming and crop diversification. Strategies that have proven successful in more developed contexts may require adaptation and supportive infrastructure in less developed regions. In areas where local economies rely on cash crops, producing cereals may not be a viable alternative. Integrating livestock into cropping systems—such as crop-tree-livestock configurations—can enhance nutrient cycling and support the production of diverse food products for human and animal consumption.

CONCLUSION

Global agrifood systems are significant contributors to GHG emissions, intensifying the impacts of climate change, while growing food demands exacerbate these challenges. At the same time, soil health has deteriorated significantly in recent decades, creating a self-reinforcing cycle of degradation from field to fork. Traditional agrifood systems have largely failed to eradicate hunger and malnutrition or to reverse worrying trends such as soil degradation, water pollution, and biodiversity loss. A sustainable agrifood production system must deliver food security while actively restoring soil health to help achieve a future

that is land degradation neutral. Our proposed triple-goal framework seeks to enhance resource use efficiencies (including water, fertilizers, and economic investment), improve soil and land quality, and increase food production from less land while boosting soil carbon sequestration. This integrated approach offers a pathway to contribute meaningfully to the UN's Sustainable Development Goals by promoting soil health and strengthening resilience to climate change. Broad adoption of the triple-goal framework could provide a strategic roadmap for transforming agrifood production globally, ensuring healthy soils, affordable and nutritious food, and reduced environmental harm. While this system alone will not eliminate global hunger, it sets the foundation for a more productive, sustainable, and resilient future for agrifood systems worldwide.

FUNDING AND ACKNOWLEDGMENTS

The views expressed in this paper are those of the authors and should not be construed as reflecting the official position of their respective affiliations. The project was supported by the National Natural Science Foundation of China (32472826), the Leading Project of the "Three Agri-Priorities with Nine Directions" Science and Technology Collaboration Plans in Zhejiang Province (2025SNJF016), the Wenzhou University research start-up fund (QD2024084), and the Wenzhou City Talent Introduction fund (R20241101). The funders had no role in the study design, data collection and analysis, decision to publish, or the preparation of the manuscript.

AUTHOR CONTRIBUTIONS

L. Wang,[1–3] G.Y.G., and M.Z., conceptualized the review. G.M.G., G.Y.G., E.A.K., and D.P.-B. contributed section materials. L.C.S. and K.F.D. brought out the critical issues relative to the subject, reviewed the draft and revisions, and provided novel ideas to improve the work. K. H.M.S. reviewed and rewrote subsections and edited versions. T.G., S.G., S.F., M.H., C.H., L. Wang,[1,6,7] and S.-J.L. contributed subsection materials to the paper. H.C., Z. Wei, J.X., and Z. Wang data collection, meta-analysis, and graphics. All authors contributed to the manuscript, agreed on the contents and authorship, and approved the final version. G.Y.G. and L. Wang[1–3] finalized the manuscript for publication.

DECLARATION OF INTERESTS

The authors declare no competing interests.

SUPPLEMENTAL INFORMATION

It can be found online at https://doi.org/10.1016/j.xinn.2025.101006.

REFERENCES

1. Tollefson, J. (2021). IPCC climate report: Earth is warmer than it's been in 125,000 years. *Nature* **596**:171–172.
2. Cheng, H. (2020). Future Earth and Sustainable Developments. *Innovation* **1**:100055. DOI: https://doi.org/10.1016/j.xinn.2020.100055.
3. van Dijk, M., Morley, T., Rau, M.L. et al. (2021). A meta-analysis of projected global food demand and population at risk of hunger for the period 2010–2050. *Nat. Food* **2**:494–501. DOI:https://doi.org/10.1038/s43016-021-00322-9.
4. Yin, Z., Zhou, B., Duan, M. et al. (2023). Climate extremes become increasingly fierce in China. *Innovation* **4**:100406. DOI:https://doi.org/10.1016/j.xinn.2023.100406.
5. Cabernard, L., Pfister, S. and Hellweg, S. (2024). Biodiversity impacts of recent land-use change driven by increases in agri-food imports. *Nat. Sustain.* **7**:1512–1524. DOI: https://doi.org/10.1038/s41893-024-01433-4.
6. Cui, Z., Zhang, H., Chen, X. et al. (2018). Pursuing sustainable productivity with millions of smallholder farmers. *Nature* **555**:363–366. DOI:https://doi.org/10.1038/nature25785.
7. Liu, M., Zheng, S., Pendall, E. et al. (2025). Unprotected carbon dominates decadal soil carbon increase. *Nat. Com* **16**:2008. DOI:https://doi.org/10.1038/s41467-025-57354-z.
8. Wheeler, T. and Von Braun, J. (2013). Climate change impacts on global food security. *Science* **341**:508–513. DOI:https://doi.org/10.1126/science.1239402.
9. Marris, E. (2022). A world without soil: the past, present, and precarious future of the Earth beneath our feet. *Nature* **601**:503–504. DOI:https://doi.org/10.1038/d41586-022-00158-8.
10. Guha, S. and Chandra, H. (2021). Measuring disaggregate level food insecurity via multivariate small area modelling: evidence from rural districts of Uttar Pradesh, India. *Food Secur.* **13**:597–615. DOI:https://doi.org/10.1007/s12571-021-01143-1.
11. Martre, P., Dueri, S., Guarin, J.R. et al. (2024). Global needs for nitrogen fertilizer to improve wheat yield under climate change. *Nat. Plants* **10**:1081–1090. DOI:https://doi.org/10.1038/s41477-024-01739-3.
12. Schneider, K., Barreiro-Hurle, J. and Rodriguez-Cerezo, E. (2023). Pesticide reduction amidst food and feed security concerns in Europe. *Nat. Food* **4**:746–750. DOI:https://doi.org/10.1038/s43016-023-00834-6.
13. Challinor, A.J., Watson, J., Lobell, D.B. et al. (2014). A meta-analysis of crop yield under climate change and adaptation. *Nat. Clim. Chang.* **4**:287–291. DOI:https://doi.org/10.1038/nclimate2153.
14. Li, L., Hong, M., Zhang, Y. et al. (2024). Soil N_2O emissions from specialty crop systems: A global estimation and meta-analysis. *Glob. Chang. Biol.* **30**:e17233. DOI:https://doi.org/10.1111/gcb.17233.
15. Tian, H., Xu, R., Canadell, J.G. et al. (2020). A comprehensive quantification of global nitrous oxide sources and sinks. *Nature* **586**:248–256. DOI:https://doi.org/10.1038/s41586-020-2780-0.
16. Gao, Y. and Cabrera Serrenho, A. (2023). Greenhouse gas emissions from nitrogen fertilizers could be reduced by up to one-fifth of current levels by 2050 with combined interventions. *Nat. Food* **4**:170–178. DOI:https://doi.org/10.1038/s43016-023-00698-w.
17. Crippa, M., Solazzo, E., Guizzardi, D. et al. (2021). Food systems are responsible for a third of global anthropogenic GHG emissions. *Nat. Food* **2**:198–209. DOI:https://doi.org/10.1038/s43016-021-00225-9.
18. Wang, F., Harindintwali, J.D., Yuan, Z. et al. (2021). Technologies and perspectives for achieving carbon neutrality. *Innovation* **2**:100180. DOI:https://doi.org/10.1016/j.xinn.2021.100180.
19. Wang, L., Lu, P., Feng, S. et al. (2024). Strategies to improve soil health by optimizing the plant–soil–microbe–anthropogenic activity nexus. *Agric. Ecosyst. Environ.* **359**:108750. DOI:https://doi.org/10.1016/j.agee.2023.108750.
20. Tang, H., Shi, L., Wen, L. et al. (2024). Effects of tillage management on soil organic carbon mineralization under double cropping rice system of southern China. *Sci. Rep.* **14**:21146. DOI:https://doi.org/10.1038/s41598-024-72042-6.
21. Even, R.J. and Francesca Cotrufo, M. (2024). The ability of soils to aggregate, more than the state of aggregation, promotes protected soil organic matter formation. *Geoderma* **442**:116760. DOI:https://doi.org/10.1016/j.geoderma.2023.116760.
22. Estrada-Carmona, N., Sánchez, A.C., Remans, R. et al. (2022). Complex agricultural landscapes host more biodiversity than simple ones: A global meta-analysis. *Proc. Natl. Acad. Sci. USA* **119**:e2203385119. DOI:https://doi.org/10.1073/pnas.2203385119.
23. Beillouin, D., Corbeels, M., Demenois, J. et al. (2023). A global meta-analysis of soil organic carbon in the Anthropocene. *Nat. Com* **14**:3700. DOI:https://doi.org/10.1038/s41467-023-39338-z.
24. Hassani, A., Azapagic, A. and Shokri, N. (2021). Global predictions of primary soil salinization under changing climate in the 21st century. *Nat. Com.* **12**:6663. DOI:https://doi.org/10.1038/s41467-021-26907-3.
25. Chen, C., Xiao, W. and Chen, H.Y.H. (2023). Mapping global soil acidification under N deposition. *Glob. Chang. Biol.* **29**:4652–4661. DOI:https://doi.org/10.1111/gcb.16813.
26. Vargas, R. (2022). Map soil nutrients to tackle imbalances worldwide. *Nature* **609**:245. DOI:https://doi.org/10.1038/d41586-022-02802-9.
27. Xia, L. and Yan, X. (2023). How to feed the world while reducing nitrogen pollution. *Nature* **613**:34–35. DOI:https://doi.org/10.1038/d41586-022-04490-x.
28. Chase, J.M., Blowes, S.A., Knight, T.M. et al. (2020). Ecosystem decay exacerbates biodiversity loss with habitat loss. *Nature* **584**:238–243. DOI:https://doi.org/10.1038/s41586-020-2531-2.
29. Ray, D.K., Ramankutty, N., Mueller, N.D. et al. (2012). Recent patterns of crop yield growth and stagnation. *Nat. Com* **3**:1293. DOI:https://doi.org/10.1038/ncomms2296.
30. Davis, K.F., Abou Ali, H., Kebede, E. et al. (2024). Where global crop yields may falter next. *Nat. Food* **5**:98–99. DOI:https://doi.org/10.1038/s43016-023-00911-w.
31. Yang, X., Xiong, J., Du, T. et al. (2024). Diversifying crop rotation increases food production, reduces net greenhouse gas emissions and improves soil health. *Nat. Com* **15**:198. DOI:https://doi.org/10.1038/s41467-023-44464-9.
32. Borrelli, P., Robinson, D.A., Fleischer, L.R. et al. (2017). An assessment of the global impact of 21st century land use change on soil erosion. *Nat. Com* **8**:2013. DOI:https://doi.org/10.1038/s41467-017-02142-7.
33. Polanin, J.R., Maynard, B.R. and Dell, N.A. (2017). Overviews in Education Research. *Rev. Educ. Res.* **87**:172–203. DOI:https://doi.org/10.3102/0034654316631117.
34. Xu, S., Delgado-Baquerizo, M., Kuzyakov, Y. et al. (2024). Positive soil priming effects are the rule at a global scale. *Glob. Chang. Biol.* **30**:e17502. DOI:https://doi.org/10.1111/gcb.17502.
35. Ascenzi, I., Hilbers, J.P., van Katwijk, M.M. et al. (2025). Increased but not pristine soil organic carbon stocks in restored ecosystems. *Nat. Com* **16**:637. DOI:https://doi.org/10.1038/s41467-025-55980-1.
36. Borenstein, M. (2022). Comprehensive Meta-Analysis Software. *Syst. Rev. Health Res* **V.3**:535–548. DOI:https://doi.org/10.1002/9781119099369.ch27.
37. Sanchez-Meca, J. and Marín-Martínez, F. (1998). Weighting by Inverse Variance or by Sample Size in Meta-Analysis: A Simulation Study. *Educ. Psychol. Meas.* **58**:211–220. DOI:https://doi.org/10.1177/0013164498058002005.
38. Leakey, R.R.B. (2018). Converting 'trade-offs' to 'trade-ons' for greatly enhanced food security in Africa: multiple environmental, economic and social benefits from 'socially modified crops'. *Food Secur.* **10**:505–524. DOI:https://doi.org/10.1007/s12571-018-0796-1.
39. Harindintwali, J.D., Zhou, J., Muhoza, B. et al. (2021). Integrated eco-strategies towards sustainable carbon and nitrogen cycling in agriculture. *J. Environ. Manage.* **293**:112856. DOI:https://doi.org/10.1016/j.jenvman.2021.112856.
40. Xiao, Z., Rasmann, S., Yue, L. et al. (2019). The effect of biochar amendment on N-cycling genes in soils: A meta-analysis. *Sci. Total Environ.* **696**:133984. DOI:https://doi.org/10.1016/j.scitotenv.2019.133984.
41. Jiao, W., Li, K., Zhou, M. et al. (2025). Determining whether biochar can effectively increase crop yields: A machine learning model development with imbalanced data. *Environ. Technol. Innov.* **38**:104154. DOI:https://doi.org/10.1016/j.eti.2025.104154.

42. Li, X., Wu, D., Liu, X. et al. (2024). A global dataset of biochar application effects on crop yield, soil properties, and greenhouse gas emissions. *Sci. Data* **11**:57. DOI:https://doi.org/10.1038/s41597-023-02867-9.
43. Bo, X., Zhang, Z., Wang, J. et al. (2023). Benefits and limitations of biochar for climate-smart agriculture: a review and case study from China. *Biochar* **5**:77. DOI:https://doi.org/10.1007/s42773-023-00279-x.
44. Xu, Z., Zhou, R. and Xu, G. (2025). Global analysis on potential effects of biochar on crop yields and soil quality. *Soil Ecol. Lett.* **7**:240267. DOI:https://doi.org/10.1007/s42832-024-0267-x.
45. Xiao, L., Lin, Y., Chen, D. et al. (2024). Maximizing crop yield and water productivity through biochar application: A global synthesis of field experiments. *Agric. Water Manag.* **305**:109134. DOI:https://doi.org/10.1016/j.agwat.2024.109134.
46. Zhang, X., Feng, X., Chai, N. et al. (2024). Biochar effects on crop yield variability. *Field Crops Res.* **316**:109518. DOI:https://doi.org/10.1016/j.fcr.2024.109518.
47. Meena, O.P., Sammauria, R., Gupta, A.K. et al. (2022). Energy-Carbon Footprint vis-a-vis System Productivity and Profitability of Diversified Crop Rotations in Semi-arid Plains of North-West India. *J. Soil Sci. Plant Nutr.* **22**:2026–2041. DOI:https://doi.org/10.1007/s42729-022-00791-2.
48. Njeru, E. (2013). Crop diversification: a potential strategy to mitigate food insecurity by smallholders in sub-Saharan Africa. *J. Agric. Food Syst. Community Dev.* **3**:1–7. DOI:https://doi.org/10.5304/jafscd.2013.034.006.
49. Gou, Z., Yin, W., Asibi, A.E. et al. (2022). Improving the sustainability of cropping systems via diversified planting in arid irrigation areas. *Agron. Sustain. Dev.* **42**:88. DOI:https://doi.org/10.1007/s13593-022-00823-2.
50. Juventia, S.D., Selin Norén, I.L.M., van Apeldoorn, D.F. et al. (2022). Spatio-temporal design of strip cropping systems. *Agr. Syst.* **201**:103455. DOI:https://doi.org/10.1016/j.agsy.2022.103455.
51. Mosquera-Losada, M.R., Santiago-Freijanes, J.J., Rois-Díaz, M. et al. (2018). Agroforestry in Europe: A land management policy tool to combat climate change. *Land Use Policy* **78**:603–613. DOI:https://doi.org/10.1016/j.landusepol.2018.06.052.
52. Lamichhane, J.R., Alletto, L., Cong, W.F. et al. (2023). Relay cropping for sustainable intensification of agriculture across temperate regions: Crop management challenges and future research priorities. *Field Crops Res.* **291**:108795. DOI:https://doi.org/10.1016/j.fcr.2022.108795.
53. Raseduzzaman, M. and Jensen, E.S. (2017). Does intercropping enhance yield stability in arable crop production ? A meta-analysis. *Eur. J. Agron.* **91**:25–33. DOI:https://doi.org/10.1016/j.eja.2017.09.009.
54. Xu, Z., Li, C., Zhang, C. et al. (2020). Intercropping maize and soybean increases efficiency of land and fertilizer nitrogen use; A meta-analysis. *Field Crops Res.* **246**:107661. DOI:https://doi.org/10.1016/j.fcr.2019.107661.
55. Li, C., Stomph, T.-J., Makowski, D. et al. (2023). The productive performance of intercropping. *Proc. Natl. Acad. Sci. USA* **120**:e2201886120. DOI:https://doi.org/10.1073/pnas.2201886120.
56. Li, X.-F., Wang, Z.-G., Bao, X.-G. et al. (2021). Long-term increased grain yield and soil fertility from intercropping. *Nat. Sustain.* **4**:943–950. DOI:https://doi.org/10.1038/s41893-021-00767-7.
57. Li, B., Li, Y.Y., Wu, H.M. et al. (2016). Root exudates drive interspecific facilitation by enhancing nodulation and N2 fixation. *Proc. Natl. Acad. Sci. USA* **113**:6496–6501. DOI:https://doi.org/10.1073/pnas.1523580113.
58. Stokeld, E., Croft, S., dos Reis, T.N.P. et al. (2023). Stakeholder perspectives on cross-border climate risks in the Brazil-Europe soy supply chain. *J. Clean. Prod.* **428**:139292. DOI:https://doi.org/10.1016/j.jclepro.2023.139292.
59. Liu, C., Plaza-Bonilla, D., Coulter, J.A. et al. (2022). Diversifying crop rotations enhances agroecosystem services and resilience. *Adv. Agron.* **173**:299–335. DOI:https://doi.org/10.1016/bs.agron.2022.02.007.
60. He, H.-m., Liu, L.-n., Munir, S. et al. (2019). Crop diversity and pest management in sustainable agriculture. *J. Integr. Agric.* **18**:1945–1952. DOI:https://doi.org/10.1016/S2095-3119(19)62689-4.
61. Owen, M.D.K., Beckie, H.J., Leeson, J.Y. et al. (2015). Integrated pest management and weed management in the United States and Canada. *Pest Manag. Sci.* **71**:357–376. DOI:https://doi.org/10.1002/ps.3928.
62. Bell, C.A., Magkourilou, E., Ault, J.R. et al. (2024). Phytophagy impacts the quality and quantity of plant carbon resources acquired by mutualistic arbuscular mycorrhizal fungi. *Nat. Com.* **15**:801. DOI:https://doi.org/10.1038/s41467-024-45026-3.
63. Beres, B.L., Harker, K.N., Clayton, G.W. et al. (2010). Weed-competitive ability of spring and winter cereals in the northern great plains. *Weed Technol.* **24**:108–116.
64. Flower, K.C., Cordingley, N., Ward, P.R. et al. (2012). Nitrogen, weed management and economics with cover crops in conservation agriculture in a Mediterranean climate. *Field Crops Res.* **132**:63–75. DOI:https://doi.org/10.1016/j.fcr.2011.09.011.
65. Meybeck, A. and Gitz, V. (2010). "Climate-Smart" Agriculture: Policies, Practices and Financing for Food Security, Adaptation and Mitigation (Food and Agriculture Organization of the United Nations).
66. Gan, Y., Liang, C., Chai, Q. et al. (2014). Improving farming practices reduces the carbon footprint of spring wheat production. *Nat. Com* **5**:5012. DOI:https://doi.org/10.1038/ncomms6012.
67. Izzeldin, M., Muradoğlu, Y.G., Pappas, V. et al. (2023). The impact of the Russian-Ukrainian war on global financial markets. *Int. Rev. Financ. Anal.* **87**:102598. DOI:https://doi.org/10.1016/j.irfa.2023.102598.
68. Behnassi, M. and El Haiba, M. (2022). Implications of the Russia–Ukraine war for global food security. *Nat. Hum. Behav.* **6**:754–755. DOI:https://doi.org/10.1038/s41562-022-01391-x.
69. Gómez, M.I., Barrett, C.B., Raney, T. et al. (2013). Post-green revolution food systems and the triple burden of malnutrition. *Food Policy* **42**:129–138. DOI:https://doi.org/10.1016/j.foodpol.2013.06.009.
70. Al-Worafi, Y.M. (2023). Malnutrition in developing countries. In Handbook Medical and Health Sciences in Developing Countries: Education, Practice, and Research, Y.M. Al-Worafi, ed. (Springer International Publishing), pp. 1–19. DOI:https://doi.org/10.1007/978-3-030-74786-2_296-1.
71. Pingali, P.L. (2012). Green Revolution: Impacts, limits, and the path ahead. *Proc. Natl. Acad. Sci. USA* **109**:12302–12308. DOI:https://doi.org/10.1073/pnas.0912953109.
72. Fan, S. and Hazell, P. (2001). Returns to public investments in the less-favored areas of India and China. *Am. J. Agric. Econ.* **83**:1217–1222.
73. Prabhu, P. (2022). Agricultural Development in Asia and Africa: Are the Lessons from the Green Revolution Relevant for Agricultural Growth and Food Security in the Twenty-First Century? (Springer Nature Singapore Pte Ltd.). DOI:https://doi.org/10.1007/978-981-19-5542-6.
74. Saleh, R. and Ehlers, M.-H. (2023). Exploring farmers' perceptions of social sustainability. *Environ. Dev. Sustain.* **27**:6371–6396. DOI:https://doi.org/10.1007/s10668-023-04140-w.
75. Satyavathi, C.T., Bharadwaj, C. and Brahmanand, P.S. (2017). Role of Farm Women in Agriculture. *Gend. Technol. Dev.* **14**:441–449. DOI:https://doi.org/10.1177/097185241001400308.
76. Janker, J., Mann, S. and Rist, S. (2019). Social sustainability in agriculture – A system-based framework. *J. Rural Stud.* **65**:32–42. DOI:https://doi.org/10.1016/j.jrurstud.2018.12.010.
77. Bardgett, R.D. and van der Putten, W.H. (2014). Belowground biodiversity and ecosystem functioning. *Nature* **515**:505–511. DOI:https://doi.org/10.1038/nature13855.
78. Horner-Devine, M.C., Carney, K.M. and Bohannan, B.J.M. (2004). An ecological perspective on bacterial biodiversity. *Proc. Biol. Sci.* **271**:113–122. DOI:https://doi.org/10.1098/rspb.2003.2549.
79. Anthony, M.A., Bender, S.F. and van der Heijden, M.G.A. (2023). Enumerating soil biodiversity. *Proc. Natl. Acad. Sci. USA* **120**:e2304663120. DOI:https://doi.org/10.1073/pnas.2304663120.
80. Koechy, M., Hiederer, R. and Freibauer, A. (2015). Global distribution of soil organic carbon - Part 1: Masses and frequency distributions of SOC stocks for the tropics, permafrost regions, wetlands, and the world. *Soil* **1**:351–365. DOI:https://doi.org/10.5194/soil-1-351-2015.
81. Shi, Z., Crowell, S., Luo, Y. et al. (2018). Model structures amplify uncertainty in predicted soil carbon responses to climate change. *Nat. Com.* **9**:2171. DOI:https://doi.org/10.1038/s41467-018-04526-9.
82. Mustafa, A., Minggang, X., Ali Shah, S.A. et al. (2020). Soil aggregation and soil aggregate stability regulate organic carbon and nitrogen storage in a red soil of southern China. *J. Environ. Manage.* **270**:110894. DOI:https://doi.org/10.1016/j.jenvman.2020.110894.
83. Wang, Y., Yao, Y., Han, B. et al. (2024). Augmenting the stability of soil aggregate carbon with nutrient management in worldwide croplands: A meta-analysis. *Agric. Ecosyst. Environ.* **370**:109052. DOI:https://doi.org/10.1016/j.agee.2024.109052.
84. Vendig, I., Guzman, A., De La Cerda, G. et al. (2023). Quantifying direct yield benefits of soil carbon increases from cover cropping. *Nat. Sustain.* **6**:1125–1134. DOI:https://doi.org/10.1038/s41893-023-01131-7.
85. Doetterl, S., Stevens, A., Six, J. et al. (2015). Soil carbon storage controlled by interactions between geochemistry and climate. *Nat. Geosci.* **8**:780–783. DOI:https://doi.org/10.1038/ngeo2516.
86. Hu, H., Chen, J., Zhou, F. et al. (2024). Relative increases in CH_4 and CO_2 emissions from wetlands under global warming dependent on soil carbon substrates. *Nat. Geosci.* **17**:26–31. DOI:https://doi.org/10.1038/s41561-023-01345-6.
87. Wang, G., Huang, Y., Zhang, W. et al. (2015). Quantifying carbon input for targeted soil organic carbon sequestration in China's croplands. *Plant Soil* **394**:57–71. DOI:https://doi.org/10.1007/s11104-015-2508-3.
88. Sun, Z., Liu, S., Zhang, T. et al. (2019). Priming of soil organic carbon decomposition induced by exogenous organic carbon input: a meta-analysis. *Plant Soil* **443**:463–471. DOI:https://doi.org/10.1007/s11104-019-04240-5.
89. Chen, L., Liu, L., Qin, S. et al. (2019). Regulation of priming effect by soil organic matter stability over a broad geographic scale. *Nat. Com* **10**:5112. DOI:https://doi.org/10.1038/s41467-019-13119-z.
90. Geller-McGrath, D., Mara, P., Taylor, G.T. et al. (2023). Diverse secondary metabolites are expressed in particle-associated and free-living microorganisms of the permanently anoxic Cariaco Basin. *Nat. Com* **14**:656. DOI:https://doi.org/10.1038/s41467-023-36026-w.
91. Sokol, N.W., Slessarev, E., Marschmann, G.L. et al. (2022). Life and death in the soil microbiome: how ecological processes influence biogeochemistry. *Nat. Rev. Microbiol.* **20**:415–430. DOI:https://doi.org/10.1038/s41579-022-00695-z.
92. Wu, H., Cui, H., Fu, C. et al. (2024). Unveiling the crucial role of soil microorganisms in carbon cycling: A review. *Sci. Total Environ.* **909**:168627. DOI:https://doi.org/10.1016/j.scitotenv.2023.168627.
93. Strickland, M.S. and Lynch, L. (2024). Decomposer communities are universal in death. *Nat. Microbiol.* **9**:585–586. DOI:https://doi.org/10.1038/s41564-023-01576-8.
94. Eisenhauer, N., Lanoue, A., Strecker, T. et al. (2017). Root biomass and exudates link plant diversity with soil bacterial and fungal biomass. *Sci. Rep.* **7**:44641. DOI:https://doi.org/10.1038/srep44641.
95. Young, I.M., Crawford, J.W., Nunan, N. et al. (2008). Microbial distribution in soils: physics and scaling (Elsevier), pp. 81–121. DOI:https://doi.org/10.1016/s0065-2113(08)00604-4.

96. Philippot, L., Raaijmakers, J.M., Lemanceau, P. et al. (2013). Going back to the roots: the microbial ecology of the rhizosphere. *Nat. Rev. Microbiol.* **11**:789–799. DOI:https://doi.org/10.1038/nrmicro3109.
97. Koegel-Knabner, I., Guggenberger, G., Kleber, M. et al. (2008). Organo-mineral associations in temperate soils:: Integrating biology, mineralogy, and organic matter chemistry. *J. Plant Nutr. Soil Sci.* **171**:61–82. DOI:https://doi.org/10.1002/jpln.200700048.
98. Six, J., Guggenberger, G., Paustian, K. et al. (2001). Sources and composition of soil organic matter fractions between and within soil aggregates. *Eur. J. Soil Sci.* **52**:607–618. DOI:https://doi.org/10.1046/j.1365-2389.2001.00406.x.
99. Bender, S.F., Wagg, C. and van der Heijden, M.G.A. (2016). An underground revolution: biodiversity and soil ecological engineering for agricultural sustainability. *Trends Ecol. Evol.* **31**:440–452. DOI:https://doi.org/10.1016/j.tree.2016.02.016.
100. Blanco-Canqui, H., Shaver, T.M., Lindquist, J.L. et al. (2015). Cover crops and ecosystem services: insights from studies in temperate Soils. *Agron. J.* **107**:2449–2474. DOI:https://doi.org/10.2134/agronj15.0086.
101. Lal, R. (2004). Soil carbon sequestration impacts on global climate change and food security. *Science* **304**:1623–1627. DOI:https://doi.org/10.1126/science.1097396.
102. Robertson, G.P. and Groffman, P.M. (2024). Chapter 14 - Nitrogen transformations. In Soil Microbiology, Ecology and Biochemistry, Fifth Edition, E.A. Paul and S.D. Frey, eds. (Elsevier), pp. 407–438. DOI:https://doi.org/10.1016/B978-0-12-822941-5.00014-4.
103. De, S.A., Madramootoo, C.A. and Whalen, J.K. (2023). Nitrogen transfer from root exudates to the rhizobiome: A 15N stem feeding method. *Soil Biol. Biochem.* **186**:109159. DOI:https://doi.org/10.1016/j.soilbio.2023.109159.
104. Curtright, A.J. and Tiemann, L.K. (2021). Intercropping increases soil extracellular enzyme activity: A meta-analysis. *Agric. Ecosyst. Environ.* **319**:107489. DOI:https://doi.org/10.1016/j.agee.2021.107489.
105. Liu, C., Jiang, M., Yuan, M.M. et al. (2023). Root microbiota confers rice resistance to aluminium toxicity and phosphorus deficiency in acidic soils. *Nat. Food* **4**:912–924. DOI:https://doi.org/10.1038/s43016-023-00848-0.
106. Selesi, D., Pattis, I., Schmid, M. et al. (2007). Quantification of bacterial RubisCO genes in soils by cbbL targeted real-time PCR. *J. Microbiol. Methods* **69**:497–503. DOI:https://doi.org/10.1016/j.mimet.2007.03.002.
107. Liu, C., Xie, J., Luo, Z. et al. (2022). Soil autotrophic bacterial community structure and carbon utilization are regulated by soil disturbance, the case of a 19-year field study. *Agriculture* **12**:1415. DOI:https://doi.org/10.3390/agriculture12091415.
108. Wang, L., Wang, Y., Sun, D. et al. (2024). Soil carbon stocks in temperate grasslands reach equilibrium with grazing duration. *Sci. Total Environ.* **949**:175081. DOI:https://doi.org/10.1016/j.scitotenv.2024.175081.
109. Anandakumar, S., Bakhoum, N., Chinnadurai, C. et al. (2022). Impact of long-term nutrient management on sequestration and dynamics of soil organic carbon in a semi-arid tropical Alfisol of India. *Appl. Soil Ecol.* **177**:104549. DOI:https://doi.org/10.1016/j.apsoil.2022.104549.
110. Cycon, M., Mrozik, A. and Piotrowska-Seget, Z. (2019). Antibiotics in the soil environment-degradation and their impact on microbial activity and diversity. *Front. Microbiol.* **10**:338. DOI:https://doi.org/10.3389/fmicb.2019.00338.
111. Kublik, S., Gschwendtner, S., Magritsch, T. et al. (2022). Microplastics in soil induce a new microbial habitat, with consequences for bulk soil microbiomes. *Front. Environ. Sci.* **10**:989267. DOI:https://doi.org/10.3389/fenvs.2022.989267.
112. Camenzind, T., Mason-Jones, K., Mansour, I. et al. (2023). Formation of necromass-derived soil organic carbon determined by microbial death pathways. *Nat. Geosci.* **16**:115–122. DOI:https://doi.org/10.1038/s41561-022-01100-3.
113. Shuaib, M., Azam, N., Bahadur, S. et al. (2021). Variation and succession of microbial communities under the conditions of persistent heavy metal and their survival mechanism. *Microb. Pathog.* **150**:104713. DOI:https://doi.org/10.1016/j.micpath.2020.104713.
114. Buenemann, E.K., Reimer, M., Smolders, E. et al. (2024). Do contaminants compromise the use of recycled nutrients in organic agriculture? A review and synthesis of current knowledge on contaminant concentrations, fate in the environment and risk assessment. *Sci. Total Environ.* **912**:168901. DOI:https://doi.org/10.1016/j.scitotenv.2023.168901.
115. Zhao, J., Chen, J., Beillouin, D. et al. (2022). Global systematic review with meta-analysis reveals yield advantage of legume-based rotations and its drivers. *Nat. Com* **13**:4926. DOI:https://doi.org/10.1038/s41467-022-32464-0.
116. Seymour, M., Kirkegaard, J.A., Peoples, M.B. et al. (2012). Break-crop benefits to wheat in Western Australia insights from over three decades of research. *Crop Pasture Sci.* **63**:1–16. DOI:https://doi.org/10.1071/CP11320.
117. Bonnet, C., Gaudio, N., Alletto, L. et al. (2021). Design and multicriteria assessment of low-input cropping systems based on plant diversification in southwestern France. *Agron. Sustain. Dev.* **41**:65. DOI:https://doi.org/10.1007/s13593-021-00719-7.
118. Tan, Q., Guo, Q., Wei, R. et al. (2023). Influence of arbuscular mycorrhizal fungi on bioaccumulation and bioavailability of As and Cd: A meta-analysis. *Environ. Pollut.* **316**:e120619. DOI:https://doi.org/10.1016/j.envpol.2022.120619.
119. Maillard, É., McConkey, B.G., St. Luce, M. et al. (2018). Crop rotation, tillage system, and precipitation regime effects on soil carbon stocks over 1 to 30 years in Saskatchewan, Canada. *Soil Tillage Res.* **177**:97–104. DOI:https://doi.org/10.1016/j.still.2017.12.001.
120. Biederman, L.A. and Harpole, W.S. (2013). Biochar and its effects on plant productivity and nutrient cycling: a meta-analysis. *Glob. Change Biol. Bioenergy* **5**:202–214. DOI:https://doi.org/10.1111/gcbb.12037.
121. Pathy, A., Pokharel, P., Chen, X. et al. (2023). Activation methods increase biochar's potential for heavy-metal adsorption and environmental remediation: A global meta-analysis. *Sci. Total Environ.* **865**:161252. DOI:https://doi.org/10.1016/j.scitotenv.2022.161252.
122. Lampkin, N. and Padel, S. (1994). The Economics of Organic Farming: An International Perspective (CAB International).
123. Ahuja, I., Dauksas, E., Remme, J.F. et al. (2020). Fish and fish waste-based fertilizers in organic farming - With status in Norway: A review. *Waste Manag.* **115**:95–112. DOI:https://doi.org/10.1016/j.wasman.2020.07.025.
124. Liu, T., Klammsteiner, T., Dregulo, A.M. et al. (2022). Black soldier fly larvae for organic manure recycling and its potential for a circular bioeconomy: A review. *Sci. Total Environ.* **833**:155122. DOI:https://doi.org/10.1016/j.scitotenv.2022.155122.
125. Haefner, F., Monzon Diaz, O.R., Tietjen, S. et al. (2023). Recycling fertilizers from human excreta exhibit high nitrogen fertilizer value and result in low uptake of pharmaceutical compounds. *Front. Environ. Sci.* **10**:1038175. DOI:https://doi.org/10.3389/fenvs.2022.1038175.
126. Lehmann, J., Rillig, M.C., Thies, J. et al. (2011). Biochar effects on soil biota - A review. *Soil Biol. Biochem.* **43**:1812–1836. DOI:https://doi.org/10.1016/j.soilbio.2011.04.022.
127. Ray, R.L., Griffin, R.W., Fares, A. et al. (2020). Soil CO_2 emission in response to organic amendments, temperature, and rainfall. *Sci. Rep.* **10**:5849. DOI:https://doi.org/10.1038/s41598-020-62267-6.
128. Zhang, X., Qian, H., Hua, K. et al. (2022). Organic amendments increase crop yield while mitigating greenhouse gas emissions from the perspective of carbon fees in a soybean-wheat system. *Agric. Ecosyst. Environ.* **325**:107736. DOI:https://doi.org/10.1016/j.agee.2021.107736.
129. Chen, Y., Guo, W., Ngo, H.H. et al. (2024). Ways to mitigate greenhouse gas production from rice cultivation. *J. Environ. Manage.* **368**:122139. DOI:https://doi.org/10.1016/j.jenvman.2024.122139.
130. Fu, J., Zhou, X., He, Y. et al. (2023). Co-application of biochar and organic amendments on soil greenhouse gas emissions: A meta-analysis. *Sci. Total Environ.* **897**:166171. DOI:https://doi.org/10.1016/j.scitotenv.2023.166171.
131. Tian, S., Zhu, B., Yin, R. et al. (2022). Organic fertilization promotes crop productivity through changes in soil aggregation. *Soil Biol. Biochem.* **165**:108533. DOI:https://doi.org/10.1016/j.soilbio.2021.108533.
132. Basiru, S. and Hijri, M. (2024). Trade-off between soil organic carbon sequestration and plant nutrient uptake in arbuscular mycorrhizal symbiosis. *Fungal Biol. Rev.* **49**:100381. DOI:https://doi.org/10.1016/j.fbr.2024.100381.
133. Velthof, G.L., Cals, T.C.A., van 't Hull, J.P. et al. (2024). Managing organic resources in agriculture: future challenges from a scientific perspective. *Front. Sustain. Food Syst.* **8**:1393190. DOI:https://doi.org/10.3389/fsufs.2024.1393190.
134. Beerling, D.J., Kantzas, E.P., Lomas, M.R. et al. (2020). Potential for large-scale CO_2 removal via enhanced rock weathering with croplands. *Nature* **583**:242–248. DOI:https://doi.org/10.1038/s41586-020-2448-9.
135. Lehmann, J., Hansel, C.M., Kaiser, C. et al. (2020). Persistence of soil organic carbon caused by functional complexity. *Nat. Geosci.* **13**:529–534. DOI:https://doi.org/10.1038/s41561-020-0612-3.
136. Swoboda, P., Döring, T.F. and Hamer, M. (2022). Remineralizing soils? The agricultural usage of silicate rock powders: A review. *Sci. Total Environ.* **807**:150976. DOI:https://doi.org/10.1016/j.scitotenv.2021.150976.
137. Griffis, T.J., Chen, Z., Baker, J.M. et al. (2017). Nitrous oxide emissions are enhanced in a warmer and wetter world. *Proc. Natl. Acad. Sci. USA* **114**:12081–12085. DOI:https://doi.org/10.1073/pnas.1704552114.
138. IPCC (2021). In Climate Change 2021: The Physical Science Basis. Contribution of Working Group I to the Sixth Assessment Report of the Intergovernmental Panel on Climate Change, V.P.Z.A. Masson-Delmotte, ed. (Cambridge University Press).
139. Köpke, U. and Nemecek, T. (2010). Ecological services of faba bean. *Field Crops Res.* **115**:217–233. DOI:https://doi.org/10.1016/j.fcr.2009.10.012.
140. Abdalla, M., Hastings, A., Cheng, K. et al. (2019). A critical review of the impacts of cover crops on nitrogen leaching, net greenhouse gas balance and crop productivity. *Glob. Chang. Biol.* **25**:2530–2543. DOI:https://doi.org/10.1111/gcb.14644.
141. Schwenke, G.D., Herridge, D.F., Scheer, C. et al. (2015). Soil N_2O emissions under N_2-fixing legumes and N-fertilised canola: A reappraisal of emissions factor calculations. *Agric. Ecosyst. Environ.* **202**:232–242. DOI:https://doi.org/10.1016/j.agee.2015.01.017.
142. Yangjin, D., Wu, X., Bai, H. et al. (2021). A meta-analysis of management practices for simultaneously mitigating N_2O and NO emissions from agricultural soils. *Soil Tillage Res.* **213**:105142. DOI:https://doi.org/10.1016/j.still.2021.105142.
143. Li, Y., Chen, J., Drury, C.F. et al. (2023). The role of conservation agriculture practices in mitigating N_2O emissions: A meta-analysis. *Agron. Sustain. Dev.* **43**:63. DOI:https://doi.org/10.1007/s13593-023-00911-x.
144. Wardak, D.L.R., Padia, F.N., de Heer, M.I. et al. (2024). Zero-tillage induces significant changes to the soil pore network and hydraulic function after 7 years. *Geoderma* **447**:116934. DOI:https://doi.org/10.1016/j.geoderma.2024.116934.
145. Ruis, S.J., Blanco-Canqui, H., Jasa, P.J. et al. (2022). No-till farming and greenhouse gas fluxes: Insights from literature and experimental data. *Soil Tillage Res.* **220**:105359. DOI:https://doi.org/10.1016/j.still.2022.105359.
146. Wang, Z., Li, D., Gruda, N.S. et al. (2024). Fertilizer application rate and nutrient use efficiency in Chinese greenhouse vegetable production. *Resour. Conserv. Recycl.* **203**:107431. DOI:https://doi.org/10.1016/j.resconrec.2024.107431.

147. Peng, J., Wang, Y.-P., Houlton, B.Z. et al. (2020). Global Carbon Sequestration Is Highly Sensitive to Model-Based Formulations of Nitrogen Fixation. *Glob. Biogeochem. Cycles* **34**:e2019GB006296. DOI:https://doi.org/10.1029/2019GB006296.
148. Qiao, M., Sun, R., Wang, Z. et al. (2024). Legume rhizodeposition promotes nitrogen fixation by soil microbiota under crop diversification. *Nat. Com* **15**:2924. DOI:https://doi.org/10.1038/s41467-024-47159-x.
149. Zhang, J., Zhao, R., Li, X. et al. (2024). Potential of arbuscular mycorrhizal fungi for soil health: A review. *Pedosphere* **34**:279–288. DOI:https://doi.org/10.1016/j.pedsph.2024.02.002.
150. Deutsch, C., Inomura, K., Luo, Y.-W. et al. (2024). Projecting global biological N_2 fixation under climate warming across land and ocean. *Trends Microbiol.* **32**:546–553. DOI:https://doi.org/10.1016/j.tim.2023.12.007.
151. Flora, Y., Rabha, P., Shinde, A. et al. (2021). Non-symbiotic bacteria for soil nitrogen fortification. *Sustainable Agriculture Reviews* **52**:417–435. DOI:https://doi.org/10.1007/978-3-030-73245-5_13.
152. Liu, Z., Rogers, B.M., Keppel-Aleks, G. et al. (2024). Seasonal CO_2 amplitude in northern high latitudes. *Nat. Rev. Earth Environ.* **5**:802–817. DOI:https://doi.org/10.1038/s43017-024-00600-7.
153. Lombardozzi, D.L., Wieder, W.R., Keppel-Aleks, G. et al. (2025). Agricultural fertilization significantly enhances amplitude of land-atmosphere CO_2 exchange. *Nat. Com* **16**:1742. DOI:https://doi.org/10.1038/s41467-025-56730-z.
154. Anastasiou, E., Fountas, S., Voulgaraki, M. et al. (2023). Precision farming technologies for crop protection: A meta-analysis. *Smart Agric. Technol.* **5**:100323. DOI:https://doi.org/10.1016/j.atech.2023.100323.
155. Li, S., Zhao, L., Wang, C. et al. (2023). Synergistic improvement of carbon sequestration and crop yield by organic material addition in saline soil: A global meta-analysis. *Sci. Total Environ.* **891**:164530. DOI:https://doi.org/10.1016/j.scitotenv.2023.164530.
156. Zolkos, S.G., Goetz, S.J. and Dubayah, R. (2013). A meta-analysis of terrestrial aboveground biomass estimation using lidar remote sensing. *Remote Sens. Environ.* **128**:289–298. DOI:https://doi.org/10.1016/j.rse.2012.10.017.
157. Wang, J., Hao, Q.H., Tu, Y. et al. (2022). The relationship between negative life events and internet addiction disorder among adolescents and college students in China: A systematic review and meta-analysis. *Front. Psychia.* **13**:e799128. DOI:https://doi.org/10.3389/fpsyt.2022.799128.
158. Bunge, A.C., Wood, A., Halloran, A. et al. (2022). A systematic scoping review of the sustainability of vertical farming, plant-based alternatives, food delivery services and blockchain in food systems. *Nat. Food* **3**:933–941. DOI:https://doi.org/10.1038/s43016-022-00622-8.
159. Sarker, M.N.I., Wu, M., Alam, G.M.M. et al. (2019). Role of climate smart agriculture in promoting sustainable agriculture: a systematic literature review. *Int. J. Agric. Resour. Gov. Ecol.* **15**:323–337. DOI:https://doi.org/10.1504/IJARGE.2019.104199.
160. Zhang, W., Dong, A., Liu, F. et al. (2022). Effect of film mulching on crop yield and water use efficiency in drip irrigation systems: A meta-analysis. *Soil Tillage Res.* **221**:105392. DOI:https://doi.org/10.1016/j.still.2022.105392.
161. Wang, H., Wang, N., Quan, H. et al. (2022). Yield and water productivity of crops, vegetables and fruits under subsurface drip irrigation: A global meta-analysis. *Agric. Water Manag.* **269**:107645. DOI:https://doi.org/10.1016/j.agwat.2022.107645.
162. Villiger, L., Joung, J., Koblan, L. et al. (2024). CRISPR technologies for genome, epigenome and transcriptome editing. *Nat. Rev. Mol. Cell Biol.* **25**:464–487. DOI:https://doi.org/10.1038/s41580-023-00697-6.
163. Trivedi, P., Leach, J.E., Tringe, S.G. et al. (2020). Plant–microbiome interactions: from community assembly to plant health. *Nat. Rev. Microbiol.* **18**:607–621. DOI:https://doi.org/10.1038/s41579-020-0412-1.
164. Panigrahi, S., Verma, K. and Tripathi, P. (2021). Review of MODIS EVI and NDVI data for data mining applications. In Data Deduplication Approaches, T.T. Thwel and G.R. Sinha, eds. (Academic Press), pp. 231–253. DOI:https://doi.org/10.1016/B978-0-12-823395-5.00018-5.
165. Iqbal, S., Riaz, U., Murtaza, G. et al. (2021). Chemical Fertilizers, Formulation, and Their Influence on Soil Health (Springer Nature Switzerland AG). DOI:https://doi.org/10.1007/978-3-030-48771-3_1.
166. Field, K.J., Daniell, T., Johnson, D. et al. (2021). Mycorrhizal mediation of sustainable development goals. *Plants, People, Planet* **3**:430–432. DOI:https://doi.org/10.1002/ppp3.10223.
167. Li, X., Zhao, R., Li, D. et al. (2023). Mycorrhiza-mediated recruitment of complete denitrifying Pseudomonas reduces N_2O emissions from soil. *Microbiome* **11**:45. DOI:https://doi.org/10.1186/s40168-023-01466-5.
168. Tzachor, A., Richards, C.E. and Jeen, S. (2022). Transforming agrifood production systems and supply chains with digital twins. *npj Sci. Food* **6**:47. DOI:https://doi.org/10.1038/s41538-022-00162-2.
169. Basso, B. and Antle, J. (2020). Digital agriculture to design sustainable agricultural systems. *Nat. Sustain.* **3**:254–256. DOI:https://doi.org/10.1038/s41893-020-0510-0.
170. Shakoor, A., Shahbaz, M., Farooq, T.H. et al. (2021). A global meta-analysis of greenhouse gases emission and crop yield under no-tillage as compared to conventional tillage. *Sci. Total Environ.* **750**:142299. DOI:https://doi.org/10.1016/j.scitotenv.2020.142299.
171. Pingali, P.R.,E. (2010). Handbook of Agricultural Economics (Agricultural and biological sciences). https://www.sciencedirect.com/handbook/handbook-of-agricultural-economics/vol/4/suppl/C.
172. Zougmoré, R., Partey, S., Ouédraogo, M. et al. (2016). Toward climate-smart agriculture in West Africa: a review of climate change impacts, adaptation strategies and policy developments for the livestock, fishery and crop production sectors. *Agric. Food Secur.* **5**:1–16. DOI:https://doi.org/10.1186/s40066-016-0075-3.
173. Field, K.J., Carrillo, Y., Campbell, S.A. et al. (2024). Innovation in plant and soil sciences to tackle critical global challenges. *Plants, People, Planet* **6**:1153–1158. DOI:https://doi.org/10.1002/ppp3.10520.
174. Bertoglio, R., Corbo, C., Renga, F.M. et al. (2021). The Digital Agricultural Revolution: A Bibliometric Analysis Literature Review. *IEEE Access* **9**:134762–134782. DOI:https://doi.org/10.1109/Access.2021.3115258.
175. Anonymous (2024). Progress towards the sustainable development goals, report of the secretary-general. In Econ. Social Council 2024 Session, pp. 1–189.
176. Kahn, L.H. (2017). Perspective: The one-health way. *Nature* **543**:47. DOI:https://doi.org/10.1038/543S47a.
177. Schulte, L.A., Dale, B.E., Bozzetto, S. et al. (2021). Meeting global challenges with regenerative agriculture producing food and energy. *Nat. Sustain.* **5**:384–388. DOI:https://doi.org/10.1038/s41893-021-00827-y.
178. Zhao, T., Wang, S., Ouyang, C. et al. (2024). Artificial intelligence for geoscience: Progress, challenges, and perspectives. *Innovation.* **5**:100691. DOI:https://doi.org/10.1016/j.xinn.2024.100691.

The Innovation, Volume *6*

Supplemental Information

Integrated strategies for enhancing agrifood productivity, lowering greenhouse gas emissions, and improving soil health

Li Wang, Gina Marie Garland, Tida Ge, Shiqian Guo, Endalkachew Abebe Kebede, Chengang He, Mohamed Hijri, Daniel Plaza-Bonilla, Lindsay C. Stringer, Kyle Frankel Davis, Soon-Jae Lee, Shoujiang Feng, Li Wang, Zhenyang Wei, Hanwen Cao, Zhi Wang, Jiexiong Xu, Kadambot H.M. Siddique, Gary Y. Gan, and Min Zhao

Supplementary file

TOWARD 'TRIPLE-GOAL' AGRIFOOD SYSTEMS

Li Wang[1,2], Gina Marie Garland[3,4], Junling Zhang[5], Tida Ge[6], Mohamed Hijri[7], Chengang He[8], Daniel Plaza-Bonilla[9], Lindsay C. Stringer[10], Endalkachew Abebe Kebede[11], Kyle Frankel Davis[11,12], Soon-Jae Lee[13], Shoujiang Feng[2], Zhenyang Wei[2], Hanwen Cao[2], Zhi Wang[2], Jiexiong Xu[2], Kadambot H.M Siddique[14], Fusuo Zhang[5*], Gary Y. Gan[2,15*], Min Zhao[2*]

Includes: More descriptions on Methods used in the study

Table S1, Table S2, Table S3

Figure S1

Figure S2

Figure S3

Figure S4

Figure S5

Figure S6

Figure S7

More descriptions on Methods

Rationale of Using A 'Second-Order Meta-Analysis' (Abbreviation SOMA)

Numerous studies have investigated the impact of crop- and soil-related anthropogenic activities on agrifood production, soil health, and GHG emissions, and the results have been documented in various scientific literatures through a rapidly growing number of first-order meta-analyses. However, most of the first-order meta-analyses synthesize the findings on individual issues, lacking a comprehensive understanding of multi-factor effects and their interactions. A close examination of individual first-order meta-analysis reveals that their results are highly variable, inconsistent, and sometimes contradictory. This was due to large variations in their research scopes, scales, experimental structure (e.g., treatment complex versus simplicity), and the number of experiments included in the original meta-analyses. Also, original studies were conducted under various soil-climatic conditions across different geographical regions, and the results differed substantially among the studies that were included in the first-order meta-analysis[1].

In contrast, a large review can be time-consuming and expensive, but it has a better chance of identifying underlying patterns of variability that may be of use to the field. A SOMA is less costly and less time-consuming while providing sufficient power of identifying novel findings, as a SOMA is designed to synthesize the results of multiple first-order meta-analyses in a quantitative and comprehensive way. A SOMA differs from a typical first-order meta-analysis which synthesizes the results of systematic review of published articles on a specific subject. Also, a SOMA differs from a conventional literature review which typically synthesizes available evidence on a certain topic utilizing pre-specified eligibility criteria for including articles with a systematic method for its production. A SOMA provides a robust way to deal with the heterogeneities across the various studies, enabling to foster the impact of soil- and crop-related anthropogenic activities on agrifood system productivity, GHG emissions, and soil health. Moreover, a SOMA approach represents an economical means of providing an answer to big questions in research determinations[2]. The strongest point in a SOMA is its ability to provide evidence to answer a general question by taking a substantive body of hard data into

consideration. The synthesis with the validation process of a SOMA indicates that the approach is an adequate technique for synthesizing effect sizes and estimating the average effect size in relation to a specific phenomenon. A SOMA allows for using moderator analysis to answer more specific questions pertaining to various study features of interest. By applying the standard procedures of systematic reviews to the synthesis of meta-analyses, the SOMA is intended to capture the essence of the existing body of literature on the subject. Additionally, the SOMA approach may prove to be helpful when reliable answers to global questions are required within limited time frames and with limited resources.

Article selection criteria and logistic steps

In the SOMA, we took the following logistic steps:

First, we defined and listed the subject areas to be discussed in our article, aiming at finding how anthropogenic activities relative to crop and soil management practices impact the triple-goal system.

Second, we identified original meta-analysis articles closely relevant to the defined subject areas through searching for the most popular academic search engines Web of Science-AHCI, Web of Science-SCIE, Web of Science-SSCI, Elsevier ScienceDirect, and Google Scholar. As a result, 190 out of 4712 meta-analyses in the subject area of interest were identified that might meet the study objectives (**Figure S1**).

Third, we preset article selection criteria: an article had (1) analyzed the effect of one or several factors on at least two of the three factors (food, soil, emissions), (2) presented a statistical analysis of at least two primary studies on at least two of the three factors, (3) reported indicators of precision of the effect sizes (standard errors, standard deviation, or confidence intervals), and (4) provided details on the methods used in the original studies which can be found in the paper or supplementary files.

Fourth, we entered the basic information presented in each of the 190 original meta-analyses into a spreadsheet (final version to be disclosed via figshare at https://figshare.com/) and then we extracted relevant data on emissions, soil health, and

crop yield or system productivity from the individual articles to generate a Master spreadsheet. A careful examination of the 190 articles during and after the data extraction ensured that each meta-analysis contained at least two of the three factors (food, soil, emissions) in the same article.

Removing overlayed articles

The same article may have been selected by different original meta-analyses, which may have some sort of 'cumulative effects' with time and space overlayed. For instance, an article describing the effect of AM fungi on soil health may have been selectively used by multiple (say six) original meta-analyses. If all those six meta-analyses were to be included in our SOMA, it would result in a 'cumulative effect' of the AM fungi effects. In this case, the requirement of statistical independence to test the classes of the moderator may be a concern. To avoid this sort of pseudo-replication, we examined all the references in the identified original meta-analyses and calculated the proportion of shared original studies among the meta-analyses, and we found that overlayed articles in the different original meta-analyses were fewer than 20%, thus, the 'cumulative effects' with time and space is at a low and acceptable level based on the findings by Florence et al[3]. This process strikes a balance between reducing significant dependence, but maintained important large studies. So, a smaller than 25% amounts of overlap is considered reasonable[4]. Thus, we included only original meta-analyses with a maximum of 25% of shared original studies in the SOMA. This cutoff was based on (1) the studies focusing only on the three pillars of interest in our study, (2) the list of studies was included in their original first-order meta-analyses, and (3) the effect of anthropogenic activities (soil and crop management-related practices) on the response variables was summarized. As a result, the search yielded **104** original meta-analyses that fit our preset selection criteria (**Table S1**).

Data extraction & management

For each of the 104 selected articles, we extracted data from the control and the response groups. All the effect sizes of the original meta-analyses were then extracted from the text, tables or figures. We used WebPlotDigitizer (https://automeris. io/WebPlotDigitizer/)

to extract data from figures of original meta-analyses. The list of primary studies (and their DOIs, when available) used in each meta-analysis was retrieved, allowing us to identify the number of common primary studies between each pair of meta-analyses when needed. Finally, we characterized the meta-analyses included in our study by pre-set criteria related to the literature search and potential bias analysis. We entered the basic information of each selected article into a spreadsheet (https://figshare.com). A total of 39,162 studies or field experiments with over 300,000 observations were recorded in the 104 meta-analyses (**Table S2**), and the studies were conducted across the globe (Global meta-analyses) or in specific regions (Regional meta-analyses).

Effect size

Effect Sizes: We extracted the ratio, percentage change, possible transformations, confidence intervals or other indicators of variability, and the number of primary studies and observations that were used to calculate the effect sizes. Also, we extracted and collected the pooled effect sizes (e.g., mean differences, odds ratios) and their variances (standard errors, confidence intervals) from each first-order meta-analysis.

In the SOMA, we use a random-effects model of the Comprehensive Meta Analysis (CMA)—one of the best meta-analysis tools at the present time. The CMA's build-in features can handle two levels of effect sizes: the observed effect sizes (Hedges' g) from each first-order meta-analysis, and the distribution of these effects across meta-analyses. This is based on the assumptions that each first-order meta-analysis provided an unbiased estimate, and that the variances were known or estimated. Also, heterogeneity measures like Tau^2 and I^2 are built to quantify between-study variance. The SOMA combines these, considering both the variance within each meta-analysis and the variance between them.

Standardization: To ensure consistency in the SOMA metrics, we standardized mean differences by converting all the effect size to **Hedges' g** (a bias-corrected standardized mean difference).

Cross-Validation

A cross validation offers support for the accuracy of the effect size synthesis and the SOMA results. The extensive literature search and a systematic review process resulted in the inclusion of 104 meta-analyses with overlap below 25% in the 39,000+ primary studies, resulting in an overall effect size of **0.67** under the random effects model, which is significantly different from zero. To validate our SOMA, we extracted 216 individual, independent effect sizes from 87 out of 104 articles which reported effect size or ratios, leading to a mean effect size of **0.48** under the random effects model. The average effect size **0.67** in our analysis is substantially greater than 0.48 in the 'sampled' meta-analyses. Effect size is a quantitative measure of the magnitude of the experimental effect. The larger the effect size the stronger the relationship between an experimental group and the control group for the target variable. When the sample size increases, the power for detecting a given effect size increase. In other words, increasing sample size while holding statistical power constant at a particular level (e.g., 95%) allows to detect a smaller effect size at that level or the chance of detecting a given effect size with a given power, where increased precision is what allowed us to detect a smaller effect with a larger sample.

Publication bias

An important issue with meta-analyses is publication bias that indicates an association between the publication status of a manuscript and the magnitude of the effect found. In our analysis, potential publication bias was assessed with funnel plots (**Figure S7**). Studies with high precision will be plotted near the average mean effect, and studies with low precision will be spread on both sides. To minimize publication bias, we took the following approaches:

1) Included meta-analyses describing experiments conducted in a field setting, and excluded meta-analyses of laboratory experiments, case studies, survey data, and qualitative studies;
2) Included meta-analyses that incorporated studies with a control group or a proxy for a control group, that incorporated both within- and between-subject designs and multiple measurement points, and that provided (either in the article, through open

access sources, or in correspondence with the authors) adequate statistics for us to calculate both an effect size measure and a measure of dispersion. These measures helped minimized potential publication bias as the funnel plot showed only weak indications of asymmetry;

3) Excluded low-quality studies or studies with insufficient information to calculate effect sizes or SEs;
4) Assessed publication bias for all accessible effect sizes reported in the original meta-analyses; and
5) Tested the sensitivity of the results against publication bias using Rosenthal fail-safe number, i.e., the number of additional studies with a mean null result necessary to provide a non-significant global estimated effect. The CMA model we used provide Frequentist model parameters estimated by maximum likelihood; this gives some sense of the robustness of our model results.

Limitations

Our search resulted in 104 first-order meta-analyses that were included in the present study. We first searched articles with "meta-analysis" in the article title. The rationale was that the researchers would include the term "meta-analysis" or equivalent in the title along with the descriptors. Then we searched the keywords targeting the Food – Soil – Emission metrics. An article must present at least two of the three goals in the same article, assuring our moderator analyses include sufficient studies in each goal. If the original meta-analysis did not report moderator analyses, we could not report it in the SOMA. We averaged effect sizes when multiple effect sizes were reported for a single outcome, ignoring any within-subject variability[4].

We used CMA random-effect models, given the relatively large number of studies (104) for moderator analyses[5]. Relatedly, readers must always be cautious in interpreting findings from a meta-analytic study since the analysis builds off existing published studies, which have their own set of limitations. Our study did not fully examine or discuss the specific practices of original first-order researchers. This could also have explanatory power for the effect sizes. Also, our operationalization of quality does not

consider all possible study components, and we did not verify beyond what the authors reported.

References

1 Gurevitch, J., Koricheva, J., Nakagawa, S. & Stewart, G. Meta-analysis and the science of research synthesis. *Nature* **555**, 175-182 (2018). https://doi.org/10.1038/nature25753

2 Tamim, R. M., Bernard, R. M., Borokhovski, E., Abrami, P. C. & Schmid, R. F. What forty years of research says about the impact of technology on learning: A second-order meta-analysis and validation study. *Review of Educational Research* **81**, 4-28 (2011).

3 Martin, F., Sun, T., Westine, C. D. & Ritzhaupt, A. D. Examining research on the impact of distance and online learning: A second-order meta-analysis study. *Educational Research Review* **36**, 100438 (2022). https://doi.org/https://doi.org/10.1016/j.edurev.2022.100438

4 Polanin, J., Maynard, B. & Dell, N. A. Overviews in education research: A systematic review and analysis. *Review of Educational Research* **87** (2016). https://doi.org/10.3102/0034654316631117

5 Borenstein, M. in *Systematic Reviews in Health Research. https://doi.org/10.1002/9781119099369.ch27* 535-548 (2022).

6 Lupwayi, N. Z., Larney, F. J., Blackshaw, R. E., Kanashiro, D. A. & Pearson, D. C. Phospholipid fatty acid biomarkers show positive soil microbial community responses to conservation soil management of irrigated crop rotations. *Soil Tillage Res.* **168**, 1-10 (2017). https://doi.org/https://doi.org/10.1016/j.still.2016.12.003

7 Schmidt, R., Gravuer, K., Bossange, A. V., Mitchell, J. & Scow, K. Long-term use of cover crops and no-till shift soil microbial community life strategies in agricultural soil. *PLoS One* **13**, e0192953 (2018). https://doi.org/10.1371/journal.pone.0192953

8 Wang, Y. *et al.* Long-term no-tillage and organic input management enhanced the diversity and stability of soil microbial community. *Sci. Total Environ.* **609**, 341-347 (2017). https://doi.org/https://doi.org/10.1016/j.scitotenv.2017.07.053

9 Chuntao Yin, N. M., Scot Hulbert, Daniel Schlatter, Timothy C. Paulitz, Kurtis Schroeder, Aaron Prescott, Amit Dhingra. Bacterial Communities on Wheat Grown Under Long-Term Conventional Tillage and No-Till in the Pacific Northwest of the United States. *Phytobiomes Journal* **1**, 83-90 (2017). https://doi.org/10.1094/pbiomes-09-16-0008-r

10 Wyngaard, N., Franklin, D. H., Habteselassie, M. Y., Mundepi, A. & Cabrera, M. L. Legacy Effect of Fertilization and Tillage Systems on Nitrogen Mineralization and Microbial Communities. *Soil Sci. Soc. Am. J.* **80**, 1262-1271 (2016). https://doi.org/10.2136/sssaj2016.03.0070

11 Somenahally, A. *et al.* Microbial communities in soil profile are more responsive to legacy effects of wheat-cover crop rotations than tillage systems. *Soil Biol. Biochem.* **123**, 126-135 (2018). https://doi.org/https://doi.org/10.1016/j.soilbio.2018.04.025

12 Tyler, H. L. Bacterial community composition under long-term reduced tillage and no till management. *J. Appl. Microbiol.* **126**, 1797-1807 (2019). https://doi.org/10.1111/jam.14267

13 Laudicina, V. A., Novara, A., Barbera, V., Egli, M. & Badalucco, L. Long-Term Tillage and Cropping System Effects on Chemical and Biochemical Characteristics of Soil Organic Matter in a Mediterranean Semiarid Environment. *Land Degrad. Dev.* **26**, 45-53 (2015). https://doi.org/10.1002/ldr.2293

14 Babin, D. *et al.* Impact of long-term agricultural management practices on soil prokaryotic communities. *Soil Biol. Biochem.* **129**, 17-28 (2019). https://doi.org/10.1016/j.soilbio.2018.11.002

15 Sommermann, L. *et al.* Fungal community profiles in agricultural soils of a long-term field trial under different tillage, fertilization and crop rotation conditions analyzed by high-throughput ITS-amplicon sequencing. *PLoS ONE* **13**, e0195345 (2018). https://doi.org/10.1371/journal.pone.0195345

16 Le Guillou, C. *et al.* Tillage intensity and pasture in rotation effectively shape soil microbial communities at a landscape scale. *MicrobiologyOpen* **8**, e00676 (2019). https://doi.org/10.1002/mbo3.676

17 Henneron, L. *et al.* Fourteen years of evidence for positive effects of conservation agriculture and organic farming on soil life. *Agron. Sust. Dev.* **35**, 169-181 (2015). https://doi.org/10.1007/s13593-014-0215-8

18 Yangjin, D., Wu, X., Bai, H. & Gu, J. A meta-analysis of management practices for simultaneously mitigating N2O and NO emissions from agricultural soils. *Soil Tillage Res.* **213**, 105142 (2021). https://doi.org/https://doi.org/10.1016/j.still.2021.105142

19 Shakoor, A. *et al.* A global meta-analysis of greenhouse gases emission and crop yield under no-tillage as compared to conventional tillage. *Sci. Total Environ.* **750** (2021). https://doi.org/10.1016/j.scitotenv.2020.142299

20 Li, Y. *et al.* The role of conservation agriculture practices in mitigating N2O emissions: A meta-analysis. *Agron. Sust. Dev.* **43**, 63 (2023). https://doi.org/10.1007/s13593-023-00911-x

21 Ruis, S. J., Blanco-Canqui, H., Jasa, P. J. & Jin, V. L. No-till farming and greenhouse gas fluxes: Insights from literature and experimental data. *Soil Tillage Res.* **220** (2022). https://doi.org/10.1016/j.still.2022.105359

Table S1. Basic information on the 104 meta-analysis articles, including number of studies and total observations reported in the published articles.

Code	Reference	Article title	Publication year	# of studies (expt.)	Total obs.	Number of test sites	Effect size estimate model	Geographic area	Main treatment	Main effect	[1] Dropdown for all the references used in the original meta-analysis articles
1	Sun et al. 2021	Elevated CO2 shifts soil microbial communities from K- to r-strategists	2021/03/12	122	965		Random	Globe	Elevated CO2	shift soil microbial communities from K- to r-strategists	
2	Rocci et al. 2021	Soil organic carbon response to global environmental change depends on its distribution between mineral-associated and particulate organic matter: A meta-analysis	2021/06/22	168	216		Random	Globe	soil distribution between mineral-associated and particulate organic matter	Soil organic carbon response to global environmental change	Allard V, Newton PCD, Lieffering M, Soussana JF, Carran RA, Matthew C (2005) Increased quantity
3	You et al. 2022	Global meta-analysis of terrestrial nitrous oxide emissions and associated functional genes under nitrogen addition	2022/2/*	144	2068		Random	Globe	nitrogen addition	terrestrial nitrous oxide emissions and associated functional genes	Long-term fertilization in the activity and community structure of ammonia oxidizers3
4	Nunes et al. 2020	Biological soil health indicators respond to tillage intensity: A US meta-analysis	2020/06/15	302			Random	US	Biological soil health indicators	tillage intensity	
5	Mondal et al. 2020	A global analysis of the impact of zero-tillage on soil physical condition, organic carbon content, and plant root response	2019/11/07	522	4131		Random	Globe	zero-tillage	soil physical condition, organic carbon content, and plant root response	Supplementary Information
6	Zheng et al. 2019	Irrigation leads to greater maize yield at higher water productivity and lower environmental costs: a global meta-analysis	2019/03/07	162	1490	21	Random	Globe	Irrigation	greater maize yield at higher water productivity and lower environmental costs	Database used for analysis yield and WP in this study collected from peer-reviewed literatures from 1970 to 2018
7	Huang et al. 2018	Greenhouse gas emissions and crop yield in no-tillage systems: A meta-analysis	2019/3/*	90	740		Random	Globe	no-tillage systems	Greenhouse gas emissions and crop yield	Ahmad, S., Li, C., Dai, G., Zhan, M., Wang, J., Pan, S., Cao, C., 2009. Greenhouse gas emission
8	Dai et al. 2018	Long-term nitrogen fertilization decreases bacterial diversity and favors the growth of Actinobacteria and Proteobacteria in agro-ecosystems across the globe	2018/04/12	70	427		Random	Globe	Long-term nitrogen fertilization	decreases bacterial diversity and favors the growth of Actinobacteria and Proteobacteria in agro-ecosystems across the globe	
9	Wang et al. 2016	Denitrification in upland of China: Magnitude and influencing factors	2016/12/09	39	300		Random	China	Denitrification in upland of China	Magnitude and influencing factors	

10	Tan et al. 2023	Influence of arbuscular mycorrhizal fungi on bioaccumulation and bioavailability of As and Cd: A meta-analysis	2023/1/*	194	1430		Random	Globe	rbuscular mycorrhizal fungi	bioaccumulation and bioavailability of As and Cd	
11	Beillouin et al. 2023	A global meta-analysis of soil organic carbon in the Anthropocene	2023/06/22	25000	190200		Random	Globe		soil organic carbon	Data availability
12	Morugán-Coronado et al. 2022	The impact of crop diversification, tillage and fertilization type on soil total microbial, fungal and bacterial abundance: A worldwide meta-analysis of agricultural sites	2022/01/22	393			Random	Globe	crop diversification, tillage and fertilization type	soil total microbial, fungal and bacterial abundance	References*
13	Muhammad et al. 2021	Cover cropping enhances soil microbial biomass and affects microbial community structure: A meta-analysis	2021/1/*	81	1824	81	Random	Globe	Cover cropping	enhances soil microbial biomass and affects microbial community structure	Reference list for the meta-analysis
14	Curtright et al. 2021	Intercropping increases soil extracellular enzyme activity: A meta-analysis	2021/05/27	100	969		Random	Globe	Intercropping	increases soil extracellular enzyme activity	Appendix: Studies included in meta-analysis
15	Xiao et al. 2018	A meta-analysis of soil extracellular enzyme activities in response to global change	2018/8/*	132	1577	133	Random	Globe	soil extracellular enzyme activities	global change	title
16	Chagas et al. 2022	Biochar increases soil carbon pools: Evidence from a global meta-analysis	2022/3/*	169	586	184	Random	Globe	Biochar	increase soil carbon pools	
17	Wang et al. 2021	Differential effects of altered precipitation regimes on soil carbon cycles in arid versus humid terrestrial ecosystems	2021/9/*	214	845		Random	Globe	altered precipitation regimes	soil carbon cycles in arid versus humid terrestrial ecosystems	
18	Jiang et al. 2017	Higher yields and lower methane emissions with new rice cultivars	2017/05/04	18	93	21	Random	Globe	new rice cultivars	Higher yields and lower methane emissions	
19	Luo et al. 2018	Organic amendments increase crop yields by improving microbe-mediated soil functioning of agroecosystems: A meta-analysis	2018/9/*	106	690		Random	Globe	Organic amendments	improving microbe-mediated soil functioning of agroecosystem, increase crop yields	Supplementary for:1
20	Miao et al. 2019	Soil extracellular enzyme activities under long-term fertilization management in the croplands of China: a meta-analysis	2019/04/04	85			Random	China	Soil extracellular enzyme activities	long-term fertilization management in the croplands	List of references used for meta-analysis
21	Zhao et al. 2017	Roles of nitrogen, phosphorus, and potassium fertilizers in carbon sequestration in a Chinese agricultural ecosystem	2017/04/29	84	385		Random	China	nitrogen, phosphorus, and potassium fertilizers	carbon sequestration in an agricultural ecosystem	Reference

22	Zhao et al. 2019	Sustaining crop production in China's cropland by crop residue retention: A meta-analysis	2019/11/*	278	4910		Random	China	crop residue retention	Sustaining crop production in China's cropland	
23	Zhou et al. 2016	Similar responses of soil carbon storage to drought and irrigation in terrestrial ecosystems but with contrasting mechanisms: A meta-analysis	2016/07/15	195			Random	Globe	drought and irrigation	soil carbon storage, contrasting mechanisms	Text S1 A list of 179 papers from which the data were extracted for this meta-analysis
24	Gao et al. 2022	Warming-induced greenhouse gas fluxes from global croplands modified by agricultural practices: A meta-analysis	2022/5/*	104	449		Random	Globe	agricultural practices, warming	greenhouse gas fluxes	Supplementary Information
25	Han et al. 2021	Global soil organic carbon changes and economic revenues with biochar application	2021/11/*	70	389		Random	Globe	biochar application	organic carbon changes and economic revenues	
26	Li et al. 2021	Microbial-derived carbon components are critical for enhancing soil organic carbon in no-tillage croplands: A global perspective	2021/1/*	95			Random	Globe	Microbial-derived carbon components	soil organic carbon in no-tillage croplands	Reference list for meta-analysis1.
27	Borchard et al. 2019	Biochar, soil and land-use interactions that reduce nitrate leaching and N2O emissions: A meta-analysis	2019/02/15	88	608		Random	Globe	reduce nitrate leaching and N2O emissions	Biochar, soil and land-use interactions	DOI
28	Chen et al. 2018	Different responses of soil organic carbon fractions to additions of nitrogen	2018/7/*	36	296		Random	Globe	additions of nitrogen	Different responses of soil organic carbon fractions	Supplementary references
29	Chen et al. 2018	The long-term role of organic amendments in building soil nutrient fertility: a meta-analysis and review	2018/01/04	132	541	20 countries, 122study sites	Random	Globe	organic amendments	building soil nutrient fertility	References
30	Luo et al. 2019	Understanding how long-term organic amendments increase soil phosphatase activities: Insight into phoD- and phoC-harboring functional microbial populations	2019/12/*	106	599		Random	Globe	phoD- and phoC-harboring functional microbial populations organic amendments	soil phosphatase activities	References
31	Mo et al. 2020	How plastic mulching affects net primary productivity, soil C fluxes and organic carbon balance in dry agroecosystems in China	2020/8/*	144	1906	92	Random	China	plastic mulching	net primary productivity, soil C fluxes and organic carbon balance in dry agroecosystems	REFERENCES
32	Xu et al. 2019	A global meta-analysis of soil organic carbon response to corn stover removal	2019/05/26	74	409	74 expt sites,	Random	Globe	corn stover removal	soil organic carbon response	DOI
33	Huang et al. 2021	Soil organic carbon, total nitrogen, available nutrients, and yield under different straw returning methods	2021/10/*	420	6820		Random	China	different straw returning methods	Soil organic carbon, total nitrogen, available nutrients, and yield	

34	Lu et al. 2021	Decrease in soil pH has greater effects than increase in above-ground carbon inputs on soil organic carbon in terrestrial ecosystems of China under nitrogen enrichment	2021/11/*	234			Random	China	Decrease in soil pH increases in above-ground carbon inputs	soil organic carbon in terrestrial ecosystems of China	The list of 234 papers from which the data were extracted for this meta-analysis.
35	Xu et al. 2020	Long-term, amplified responses of soil organic carbon to nitrogen addition worldwide	2020/12/*	476			Random	Globe	nitrogen addition	soil organic carbon	Article
36	Geng et al. 2023	Legumes can increase the yield of subsequent wheat with or without grain harvesting compared to Gramineae crops: A meta-analysis	2023/1/*	62	453	5 continents, 18 countries	Random	Globe	yield of subsequent wheat	Legumes	Reference
37	Li et al. 2023	The role of conservation agriculture practices in mitigating N2O emissions: A meta-analysis	2023/09/04	73	281		Random	Globe	conservation agriculture practices	mitigating N2O emissions	References of the meta-analysis
38	Poeplau et al. 2015	Carbon sequestration in agricultural soils via cultivation of cover crops – A meta-analysis	2015/02/01	30	139	37sites, 139 plots	Random	Globe	cultivation of cover crops	Carbon sequestration in agricultural soils	
39	Santachiara et al. 2019	Nutritional and environmental effects on biological nitrogen fixation in soybean: A meta-analysis	2019/07/01	92	956		Random	Globe		Relative response to N fertilization, P, K and S, B, Ca, Fe and Zn	Appendix A. List of peer reviewed publications included in the meta-analysis across environmental or management factors.
40	Wang et al. 2018	Decreasing soil microbial diversity is associated with decreasing microbial biomass under nitrogen addition	2018/05/01	55	273		Random	Globe			References
41	Wang et al. 2024	Augmenting the stability of soil aggregate carbon with nutrient management in worldwide croplands	2024/08/15	269	2035		Random	Globe			Supplementary:
42	Alvarez et al. 2017	Cover crop effects on soils and subsequent crops in the pampas: A meta-analysis	2017/07/01	67	975	67	Random	Argentina			Supplementary material Table A (Bulk density)
43	Li et al. 2020	Residue retention promotes soil carbon accumulation in minimum tillage systems: Implications for conservation agriculture	2020/10/20	243	1928		Random	Globe			Reference list for meta-analysis
44	Liu et al. 2018	Climatic role of terrestrial ecosystem under elevated CO2: a bottom-up greenhouse gases budget	2018/05/07	169	1655		Random	Globe			List of 169 publications from which data were extracted for this analysis.
45	Muhammad et al. 2019	Regulation of soil CO2 and N2O emissions by cover crops: A meta-analysis	2019/09/01	48	>1000		Random	Globe			Appendix B. Information of location, climate and soil texture for each experimental

									site used for meta-analysis in this study
46	Xia et al. 2018	Trade-offs between soil carbon sequestration and reactive nitrogen losses under straw return in global agroecosystems	2018/10/08	363	3251		Random	Globe	Data S1. References of all datasets included in this meta-analysis.
47	Zhao et al 2017	Crop yields under no-till farming in China: A meta-analysis	2017/03/01	164	1006		Random	China	Selected references
48	Kan et al. 2021	Effects of experiment duration on carbon mineralization and accumulation under no-till	2021/05/01	21	57		Random	Globe	C and N mineralization of undisrupted and disrupted soil from different structural zones of conventional tillage and no-tillage systems in northern France
49	Sun et al. 2020	Climate drives global soil carbon sequestration and crop yield changes under conservation agriculture	2020/01/17	115	1970	138	Random	Globe	Lòpez-Fando & Pardo
50	Zheng et al. 2020	Drought shrinks terrestrial upland resilience to climate change	2020/07/30	128	1344		Random	Globe	List of 128 publications from which data were extracted for this analysis.
51	Jian et al. 2020	A calculator to quantify cover crop effects on soil health and productivity	2020/05/01	269	4024	269	Random	Globe	Impact of soil health management practices on soilborne pathogens, nematodes and root diseases of vegetable crops.
52	Liu et al. 2020	Increased soil release of greenhouse gases shrinks terrestrial carbon uptake enhancement under warming	2020/05/13	164	1845		Random	Globe	List of 164 references from which data were extracted for this analysis
53	Ye et al. 2019	Biochar effects on crop yields with and without fertilizer: A meta-analysis of field studies using separate controls	2019/09/16	56	264	64	Random	Globe	References
54	Zhang et al. 2019	Biochar amendment effects on the activities of soil carbon, nitrogen, and phosphorus hydrolytic enzymes: a meta-analysis	2019/06/10	43	401		Random	Globe	References
55	Deng et al. 2018	Positive responses of belowground C dynamics to nitrogen enrichment in China	2018/03/01	124	570	127	Random	Globe	A list of 63 papers from which the data were extracted for this meta-analysis.
56	Peixoto et al. 2020	Occasional tillage in no-tillage systems: A global meta-analysis	2020/11/25	68	588		Random	Globe	

57	Qiao et al. 2024	Legume rhizodeposition promotes nitrogen fixation by soil microbiota under crop diversification	2024/04/04				Random	China	
58	Bebber et al. 2022	A meta-analysis of the effect of organic and mineral fertilizers on soil microbial diversity	2022/07/01	37	65		Random	Globe	SUPPLEMENTARY REFERENCES
59	Borchard et al. 2019	Biochar, soil and land-use interactions that reduce nitrate leaching and N2O emissions: A meta-analysis	2019/02/15	88	608		Random	Globe	
60	Venter et al. 2016	The impact of crop rotation on soil microbial diversity: A meta-analysis	2016/07/01	20	281		Random	Globe	Study name
61	Davies-Barnard et al. 2020	The Global Distribution of Biological Nitrogen Fixation in Terrestrial Natural Ecosystems	2020/02/09	142	252		Random	Globe	Text S1.
62	Biederman et al. 2012	Biochar and its effects on plant productivity and nutrient cycling: a meta-analysis	2012/12/31	371	941		Random	Globe	Author
63	Estrada-Carmona et al. 2022	Complex agricultural landscapes host more biodiversity than simple ones: A global meta-analysis	2022/09/12	157	1134	29 countries sites	Random	Globe	References
64	Zhao et al. 2020	Does crop rotation yield more in China? A meta-analysis	2020/01/01	45	214		Random	China	
65	Hu et al. 2022	Responses of AM fungal abundance to the drivers of global climate change: A meta-analysis	2022/01/20	75	431		Random	Globe	Note S1 Reference list of articles included in this meta-analysis.
66	Das et al. 2022	Responses of soil organic carbon to conservation practices including climate-smart agriculture in tropical and subtropical regions: A meta-analysis	2022/01/20	84	516		Random	Globe	Supplementary Data.1: References of the articles used for data
67	Geisseler et al. 2017	Effect of fertilization on soil microorganisms in paddy rice systems e A meta-analysis	2017/12/01	55			Random	China, India, Korea, Pakistan, Taiwan	References
68	Kim et al. 2020	Do cover crops benefit soil microbiome? A meta-analysis of current research	2020/03/01	60	30		Random	Globe	
69	Liu et al. 2019	Effect of Straw Retention on Crop Yield, Soil Properties, Water Use Efficiency and Greenhouse Gas Emission in China: A Meta-Analysis	2019/07/12	176	7417		Random	Globe	The References of yield data.

70	Song et al. 2017	Altered soil carbon and nitrogen cycles due to the freeze-thaw effect: A meta-analysis	2017/06/01	46			Random	Globe			Global Change Biology 20, 2663-2673.
71	Zeng et al. 2016	Nitrogen fertilization directly affects soil bacterial diversity and indirectly affects bacterial community composition	2016/01/01				Random	Inner Mongolia, China			
72	Zhou et al. 2017	Changes in microbial biomass and the metabolic quotient with biochar addition to agricultural soils: A Meta-analysis	2017/02/15	97	1073		Random	Globe			Reference:
73	Li et al. 2024	Soil N2O emissions from specialty crop systems: A global estimation and meta-analysis	2024/03/12	114	1137		Random	Globe			Reference
74	Zhao et al. 2022	Global systematic review with meta-analysis reveals yield advantage of legume-based rotations and its drivers	2022/08/22	462	11768	53 countries	Random	Globe			Supplementary references
75	Yangjin et al. 2021	A meta-analysis of management practices for simultaneously mitigating N2O and NO emissions from agricultural soils	2021/09/01	39	952		Random	Globe			Agric Ecosys Environ 121:383-394
76	Shakoor et al. 2021	A global meta-analysis of greenhouse gases emission and crop yield under no-tillage as compared to conventional tillage	2021/01/01	50	431		Random	Globe			Rutkowska et al. (2018)
77	Xu et al. 2020	Intercropping maize and soybean increases efficiency of land and fertilizer nitrogen use; A meta-analysis	2020/02/01	88	1436		Random	Globe			
78	Raseduzzaman et al. 2017	Does intercropping enhance yield stability in arable crop production? A meta-analysis	2017/11/01	37			Random	Globe			
79	Abdalla et al. 2019	A critical review of the impacts of cover crops on nitrogen leaching, net greenhouse gas balance and crop productivity	2019/07/04	106		372	Random	Globe	cover crops	N leaching, net greenhouse gas balance and crop productivity	References
80	Bai et al. 2018	Effects of agricultural management practices on soil quality: A review of long-term experiments for Europe and China	2018/10/01	326	474	13 case sites	Random	Europe and China	agricultural management practices	soil quality	Title of paper
81	Chen et al. 2019	Effects of plant diversity on soil carbon in diverse ecosystems: a global meta-analysis	2019/10/18	121	1001		Random	Globe	plant diversity	soil carbon	Reference
82	Cheng et al. 2017	Warming enhances old organic carbon decomposition through altering functional microbial communities	2017/04/21	43			Random	Globe	Warming	Altering functional microbial communities	Reference

83	Cong et al. 2018	Impact of soil properties on the soil methane flux response to biochar addition: a meta-analysis	2018/08/24				Random	Globe	soil properties	soil methane flux response to biochar addition	
84	Davidson et al. 2017	Livestock grazing alters multiple ecosystem properties and services in salt marshes: a meta-analysis	2017/02/22	89	498		Random	Globe	Livestock grazing	multiple ecosystem properties and services in salt marshes	
85	Ding et al. 2017	A meta-analysis and critical evaluation of influencing factors on soil carbon priming following biochar amendment	2017/12/22	27	1170		Random	Globe	biochar amendment	influencing factors on soil carbon priming	
86	Du et al. 2017	The effect of no-till on organic C storage in Chinese soils should not be overemphasized: A meta-analysis	2017/01/02	95	409	57	Random	Globe	no-till	Organic C storage in soils	References
87	Elias et al. 2018	A meta-analysis of pesticide loss in runoff under conventional tillage and no-till management	2018/01/12	35			Random	Globe	conventional tillage and no-till	pesticide loss in runoff	reference
88	Gurevitch et al. 2018	Meta-analysis and the science of research synthesis	2018/03/08				Random	Globe			
89	Li et al. 2018	Liming effects on soil pH and crop yield depend on lime material type, application method and rate, and crop species: a global meta-analysis	2018/08/23	175	1337		Random	Globe	lime material type, application method and rate, and crop species	soil pH and crop yield	List of references used for the meta-analysis
90	Li et al. 2017	Long-term (≥20 years) application of fertilizers and straw return enhances soil carbon storage: a meta-analysis	2017/06/30	61	440		Random	China	Long-term (≥20 years) application of fertilizers and straw return	soil carbon storage	
91	Ma et al. 2018	Impacts of plastic film mulching on crop yields, soil water, nitrate, and organic carbon in Northwestern China: A meta-analysis	2018/04/01	83	1278		Random	China	plastic film mulching	crop yields, soil water, nitrate, and organic carbon	
92	Masuda et al. 2024	Global soil metagenomics reveals distribution and predominance of Deltaproteobacteria in nitrogen-fixing microbiome	2024/05/24	22	1451		Random	Globe			REFERENCES
93	Meurer et al. 2018	Tillage intensity affects total SOC stocks in boreo-temperate regions only in the topsoil—A systematic review using an ESM approach	2018/02/01	101			Random	Globe			References
94	Ren et al. 2018a	Responses of soil total microbial biomass and community compositions to rainfall reductions	2018/08/01	114	208		Random	Globe			Supplementary material (ii): Text S1：Studies included in the meta-analysis

95	Ren et al. 2018b	A synthetic analysis of livestock manure substitution effects on organic carbon changes in China's arable topsoil	2018/12/01	148	729	69	Random	China			References
96	Ros et al. 2016	Selenium fertilization strategies for bio-fortification of food: an agro-ecosystem approach	2016/02/19	243	3865		Random	Globe			Further study: papers used for the meta-analysis
97	Schmidt et al. 2013	Methods for second order meta-analysis and illustrative applications	2013/07/01				Random	Globe			
98	Sun et al. 2019	Priming of soil organic carbon decomposition induced by exogenous organic carbon input: a meta-analysis	2019/08/08	94	2048		Random	Globe			Text S1 Studies included in the current meta-analysis
99	Tian et al. 2018	Cropland abandonment enhances soil inorganic nitrogen retention and carbon stock in China: A meta-analysis	2018/08/21	83	295		Random	Globe			Note S1. A list of 83 publications used for collecting data in our meta-analysis
100	Vicente-Vicente et al. 2016	Soil carbon sequestration rates under Mediterranean woody crops using recommended management practices: A meta-analysis	2016/11/01	51	144		Random	Globe			Appendix A. References of the meta-analysis
101	Yuan et al. 2017	Experimental and observational studies find contrasting responses of soil nutrients to climate change	2017/06/01	323	1421	1346	Random	Globe			
102	Yue et al. 2017	Influence of multiple global change drivers on terrestrial carbon storage: additive effects are common	2017/03/28	633	3620		Random	Globe			Text S1 A list of 633 primary articles from which the data were extracted for this
103	Zheng et al. 2019	Global pattern and controls of biological nitrogen fixation under nutrient enrichment: A meta-analysis	2019/05/23	516			Random	Globe			APPENDIX: DATA SOURCES FOR META-ANALYSIS
104	Zhou et al. 2016	Changes in organic carbon and nitrogen in soil with metal pollution by Cd, Cu, Pb and Zn: a meta-analysis	2016/03/16	160	1187		Random	Globe			References

[1]Dropdown was created to show the list of all the references used in the original meta-analysis articles (Excel files) only if the list was provided by the original authors.

Table S2. The logistic steps in searching for meta-analysis articles on triple-goal system (more food, healthy soil, less emission) and preset criteria for selecting articles to be included in the SOMA.

Meta-analysis search on triple-goal articles (more food, healthy soil, less emission)

	# of meta-analysis article
Meta-analysis in agriculture or cropping	4712
Emission or N_2O or soil property or soil health or crop yield	1805
between 2015-2024	1544
emission or N_2O	385
emission AND soil health or property	190
emission AND soil health/property AND crop yield	**104**

<u>Preset selection criteria</u>

(1) analyzed the effect of one or several factors on at least two of the three factors (more food, healthier soil, fewer emissions);

(2) presented a statistical analysis of at least two primary studies on at least two of the three factors;

(3) reported indicators of precision of the effect sizes (standard errors, standard deviation, or confidence intervals);

(4) provided details on the methods used in the original studies which can be found in the paper or supplementary files.

Table S3. List of examples of using crop diversification (such as intercropping, diversified rotation), conservation agricultural practices, and improved cropping management, for fulfilling the 'triple-goal' system, i.e., more food, healthier soil, fewer emissions.

Coordinators	Study site	Study year or data reported	Crop	Soil & crop management	Physiobiological mechanism	Outcome in crop productivity and/or soil property change	Reference
**Intercropping**							
–8.05; –34.90	Recife, Brazil	Apr-23	Cactus, millet	Cactus–millet intercropping	Improved between-crops interactions	WUE ranged from 2.31–2.47 kg m^{-3}, significantly greater than sole crops	Souza et al., 2023
37.90; 102.77	Wuwei, China	Nov-20	Maize, wheat	Maize–wheat intercropping	Promoted water complementation and inter-zone water migration	Maize–wheat intercropping increased WUE by 20–50%	Yin et al., 2020
36.72; 3.15	D'Alger, Algeria	Apr-23	Chickpea, durum wheat	Chickpea–durum wheat intercropping	Promoted WUE and NUE	WUE of 0.62 kg m^{-3}, higher than sole crops	Kherif et al., 2023
36.71; 3.15	D'Alger, Algeria	Feb-23	Chickpea, wheat	Chickpea–wheat intercropping	Increased chlorophyll content, optimizing WUE	Increased WUE by 0.30–0.57 kg m^{-3} compared to sole chickpea	Bouras et al., 2023
29.63; 52.52	Shiraz, Iran	Jun-23	Chickpea, barley	Chickpea–barley intercropping	Enhanced chlorophyll content, leaf carotenoid content, and catalase and peroxidase activities	Enhanced WUE compared to sole crops	Assadi et al., 2023
29.39; 71.69	Bahawalpur, Pakistan	Nov-22	Maize, soybean	Maize–soybean strip intercropping	Species complementarities for radiation, water, and land in time and space, higher LAI, radiation use	Intercropped maize had WUE ranging from 13.3–16.2 kg ha^{-1} mm^{-1}, higher than sole crops	Raza et al., 2022
40.63; 22.96	Thessaloniki, Greece	Feb-21	Wheat, pea	Wheat–pea intercropping	Increased land equivalent ratio (LER)	Used available water more efficiently, resulting in higher yields	Pankou et al., 2021
**Crop rotation**							
–34.97; 138.6	Urrbrae, Australia	Jan-21	Cereals, legumes, oilseeds	Cereal–legume; soybean–sunflower, chickpea–flax	Promoted between species complementary, competitive and interactions; allelopathic pest repellence	Cereal–legume intercropping had higher carbon yield than soybean–sunflower and chickpea–flax cropping	Dowling et al., 2021
35.81; 50.95	Karaj, Iran	Nov-21	Sorghum, amaranth	Sorghum–amaranth intercropping	Partial root-zone irrigation; alternate furrow irrigation	Saved 20–22% irrigation water without reducing carbon yield	Baghdadi et al., 2021
–1.26; 36.82	Sub-Saharan Africa, Kenya	Nov-20	Potato, dolichos (*Lablab purpureus* L.)	Potato–dolichos or potato–hairy vetch intercropping	Increased proline (1.99–2.91 vs. 1–1.19 umol g^{-1}) and soluble carbohydrates (28–59 vs. 10–28 umol g^{-1}) under stress	Potato–legume intercropping reduced nutrient losses by 45–80%, increased yields by 2–3-fold and WUE by 2–4-fold in potato	Nyawade et al., 2020
37.97; –100.8	Garden City, Kansas	Nov-22	Winter wheat, sorghum	Wheat–forage sorghum–forage old rotation	Diversification increased cropping intensity, productivity, resource use, and gross margin	Double cropping of forage sorghum after wheat increased total carbon production	Holman et al., 2022
**Soil mulching**							
34.65; 110.53	Yangling, Northwest China	Nov-21	Wheat	Plastic mulching	Improved RUE post-anthesis, elevated canopy photosynthesis,	Increased transpiration-to-evaporation ratio, carbon source size, and sink size	Ding et al., 2021

					delayed RUE peak in reproductive period		
34.65; 110.53	Yangling, Northwest China	Jan-22	Winter wheat	Ridge–furrow combined with supplemental irrigation	Increased soil water storage and net photosynthetic rate	Decreased irrigation water by 50% without reducing crop yield	Zhang et al., 2022
34.65; 110.53	Yangling, Northwest China	Sep-22	Maize	Partial straw mulching with urea blending	Delayed leaf senescence, increased chlorophyll content, photosynthesis, and N uptake	Increased maize grain yield by 11–53% and WUE by 9–57%	Guo et al., 2022
34.65; 110.53	Yangling, Northwest China	Aug-21	Winter wheat	Ridge–furrow with optimum irrigation and N rate	Increased LAI, above-ground DW, leaf chlorophyll content, and net photosynthetic rate	Increased winter wheat yield by 14.6–17.7%, WUE by 5.0–10.0%, and NUE by 16.2–30.5%	Gu et al., 2021
**Improved irrigation**							
–3.73; –38.53	Fortaleza, Ceara, Brazil	Apr-22	Maize	Supplemental irrigation with brackish water	Reduced water stress, averted excessive salt accumulation in soil, improved CO_2 assimilation rates	Promoted physical water productivity by 1.3–3.0-fold	Cavalcante et al., 2022
31.66; 36.31	Northern Jordan	Jan-21	Pistachio (*Pistacia vera* L.)	Micro-catchment and gravel mulching	Improved soil moisture, photosynthesis (Pn), transpiration (E), and stomatal conductance	Improved carbon production in young pistachio trees in rainfed regime	Tadros et al., 2021
17.46; 78.45	Hyderabad, India	Oct-22	Cotton (*Gossypium* sp.)	Cotton in an eddy covariance system	Seasonal NEE (–333 to –392 g C m^{-2}), GPP (990–1064 g C m^{-2}), R-eco (656–672 g C m^{-2}), and ET (468–545 mm)	Increased net CO_2 sink, with seasonal ec-WUE 1.9–2.1 g C kg^{-1} of H_2O	Chakraborty et al., 2022
**Subsoil tillage**							
35.20; 110.11	Heyang, Shaanxi	May-20	Wheat, maize	Subsoil tillage alternated with plowing	Reduced bulk density by 5.19%, increased porosity 5.69% and macroaggregates by 26.92%	Promoted WUE by 7.25% and yield by 8.37%	Yu et al., 2020
34.65; 110.53	Yangling, Northwest China	Jul-21	Wheat, maize	Wheat–maize rotation with subsoil tillage	Reduced precipitation loss, maintained soil water balance by regulating water use and precipitation storage	Reduced precipitation loss by 78–135 mm during the fallow period, increased WUE by 6.5–10.8 kg ha^{-1} mm^{-1}, and grain yields	Li et al., 2021
**Conservation agriculture**							
50°03′N - 112°09′W	Vauxhall, Canada	2000-2011	Multiple rotation crops	Conventional tillage Reduced tillage	Reduced tillage increased soil microbial biomass carbon, fungal and bacterial PLFAs	Reduced tillage increased soil microbial biomass C from 428 to 509 mg C kg^{-1}.	[6]
36°20′N - 120°7′W	California, USA	1999-2013	Tomato cotton	No-tillage (NT), Conventional tillage (CT)	CT reduced number of bacteria and archaea in top 5-cm soil layer.	Tillage reduced bacteria and archaea about 4×10^7 and 1.5×10^7 in top 5cm soil layer.	[7]

35°25′N - 82°33′W	North Carolina, USA	1994-2009	Multiple species	No-tillage, CT	No-till increased PLFA	No-till increased 55% of total PLFA concentration and 22% of bacterial PLFA compared to CT	8
38°54'N - 77°2'W 45°29'N - 115°27'W	Washington and Idaho, USA	35 years before first sampling in 2008 at Washington, and 2000-2010 at Idaho	Winter wheat, spring wheat, Barley, legume	No-tillage; Conventional tillage	Tillage didn't influence soil bacterial diversity, but impacted bacterial community composition.	Tillage strongly impacted bacterial community composition. In particular, *Chitinophagaceae, Micrococcaceae, Gaiellaceae* and *Nocardioidaceae* were more frequent under tillage, while *Hyphomicrobiaceae, Koribacteraceae, Acidobacteriaceae* dominant under NT.	9
33°52'N - 83°27'W	Watkinsville, USA	1994-2013	Cotton, corn, rye, wheat, canola, pearl, millet	No-tillage; Conventional tillage	CT reduced the amount of AOA and AOB in top 20-cm soil layer.	CT reduced the amount of AOA and AOB in top 20-cm soil layer, which could slow down the potentials of N mineralization.	10
35°57'N - 98°03'W	El Reno, USA	2011-2017	Wheat, legume	No-tillage; Conventional tillage	NT increased fungal biomass carbon with inorganic N added.	Fungal biomass carbon under NT 193% higher with inorganic N added but 26% lower with organic N added, then under CT. NT+inorganic N increased 91.3% of FBC, while CT+organic N increased 93.3% of FBC.	11
32°31′N - 89°42′W	Mississippi, USA	2000-2014	Balansa clower, Abruzzi rye, cotton as cover crop	No-tillage; Reduced tillage	No till increased soil microbial biomass C.	No-till increased soil microbial biomass C (581.13 mg per kg dry soil) than reduced till (473.87 mg per kg dry soil). Relative abundance of *Betaproteobacteria* and *Azoarcus* higher under NT, but *Alphaproteobacteria*, *Rhizobiales, Sphingomonas* and *Bacillus* were higher under reduced till.	12
37°30'N - 13°31'E	Sicily, Italy	1991-2009	Wheat, bean	No-tillage (NT), Conventional tillage (CT), Reduced tillage (RT)	NT increased soil microbial carbon in wheat-pulse rotation	Both RT and NT increased MBC/TOC and reduced qCO_2 around 0.3 mg CO_2-C g^{-1} MBC h^{-1}. NT increased SMBC 120-200 mg kg^{-1} in wheat-pulse rotation, then CT.	13

51°47'N - 11°43'E	Bernburg, Germany	1992-2015	Maize, wheat, barley, rapeseed	Conventional tillage; Mouldboard plough tillage (MP)	CT increased the number of genera belonging to *Alphaproteobacteria* (e.g., *Amaricoccus, Chelatococcus, Microvirga)* and *Actinobacteria (Gaiella, Janibacter, Rubrobacter).*	CT increased the genera *Alphaproteobacteria* (e.g., *Amaricoccus, Chelatococcus, Microvirga)* and *Actinobacteria* , compared with MP.	[14]
51°47'N - 11°43'E	Bernburg, Germany	1992-2015	Maize, wheat, barley, rapeseed	Conventional tillage, Mouldboard plough tillage	CT selected fungi such as *Stagonospora, Claroideoglomus*, and *Rhizophagus*, while MP selected *Entrophospora* and *Sebacina*.	CT selected fungi such as *Stagonospora, Claroideoglomus*, and *Rhizophagus* in wheat rhizosphere, while MP selected *Entrophospora* and *Sebacina*.	[15]
48°00'N - 2°49'E	Brittany, France	1993-2013	Legumes	No-tillage, CT	Tillage significantly decreased soil microbial biomass and fungal richness.	CT decreased soil microbial biomass and fungal richness but increased bacterial richness and evenness compared to NT.	[16]
48°48′N - 2°08′E	Versailles, France	1997-2010	Wheat, pea, oilseed, maize	Conventional tillage, Conservation tillage	Conservation tillage increased soil bacteria and fungi compared to conventional tillage.	Conservation tillage increased $0.3\times\log_{10}$ g^{-1} and $0.25\times\log_{10}$ g^{-1} for soil bacteria and fungi respectively, compared to conventional tillage.	[17]

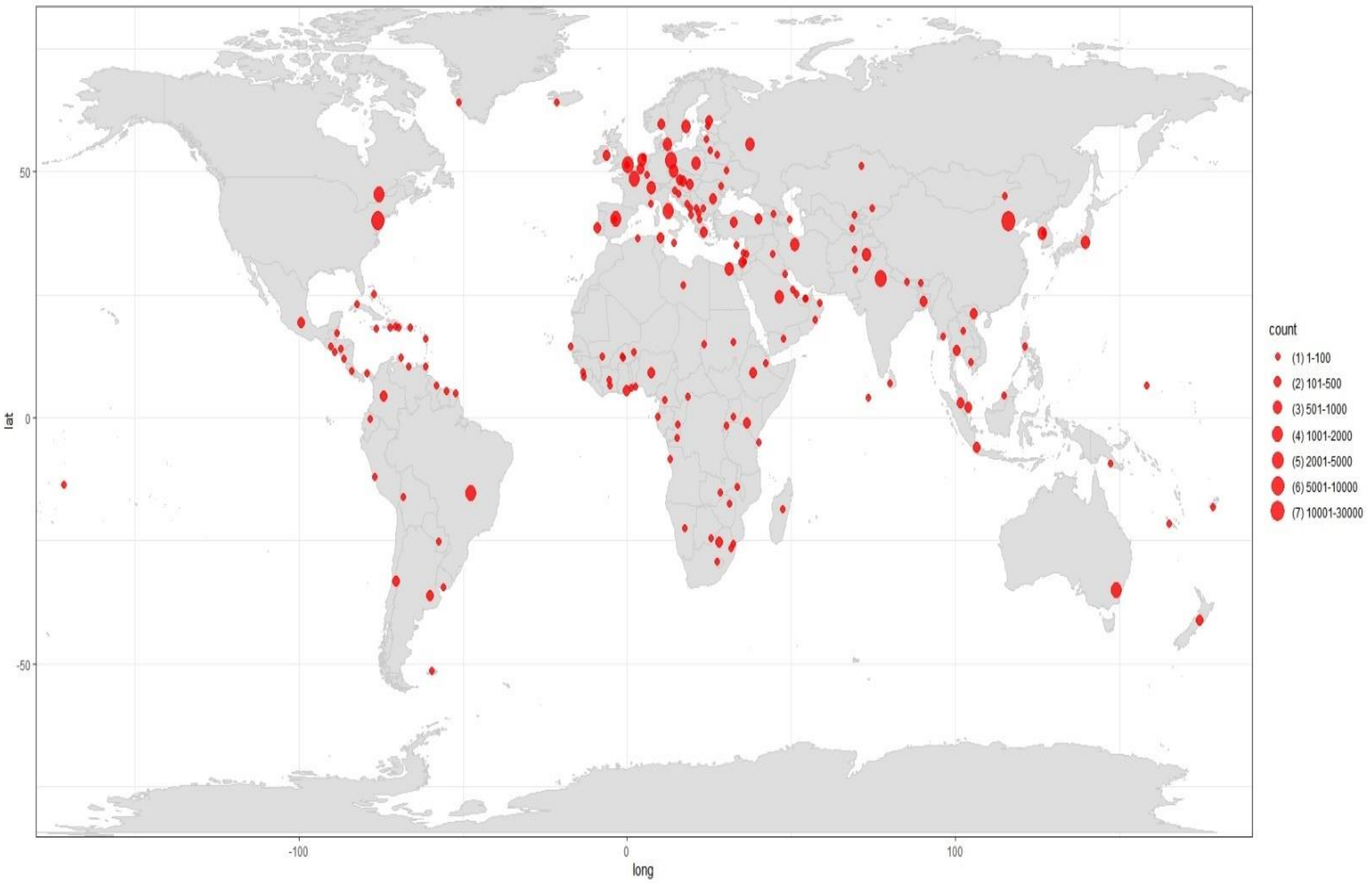

Figure S1. A global map showing the study sites reported in the 104 original first-order meta-analysis articles. The red dots represent approximate sampling sites with the dot size denoting the range of the number of studies covered in the given meta-analyses. Some dots with only a few studies are hardly visible.

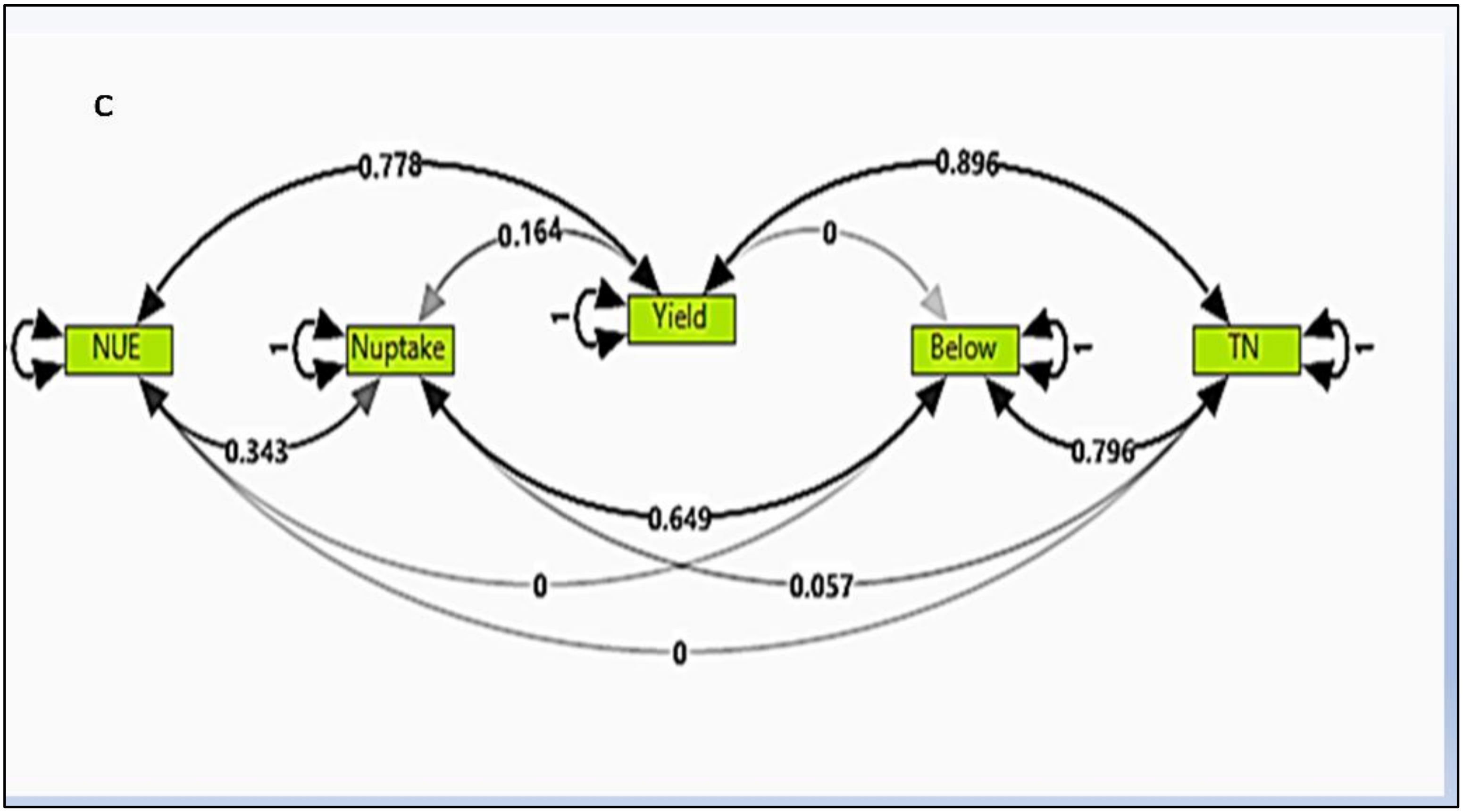

268

Figure S2. The '3-goal' system integrates existing and novel farming practices to maximize agrifood productivity and stability. Structure equation modeling demonstrates that increased systems productivity is closely linked to total N (TN) supplied and NUE among other factors, both being associated with plant N uptake (N uptake) and belowground biomass accumulation (below).

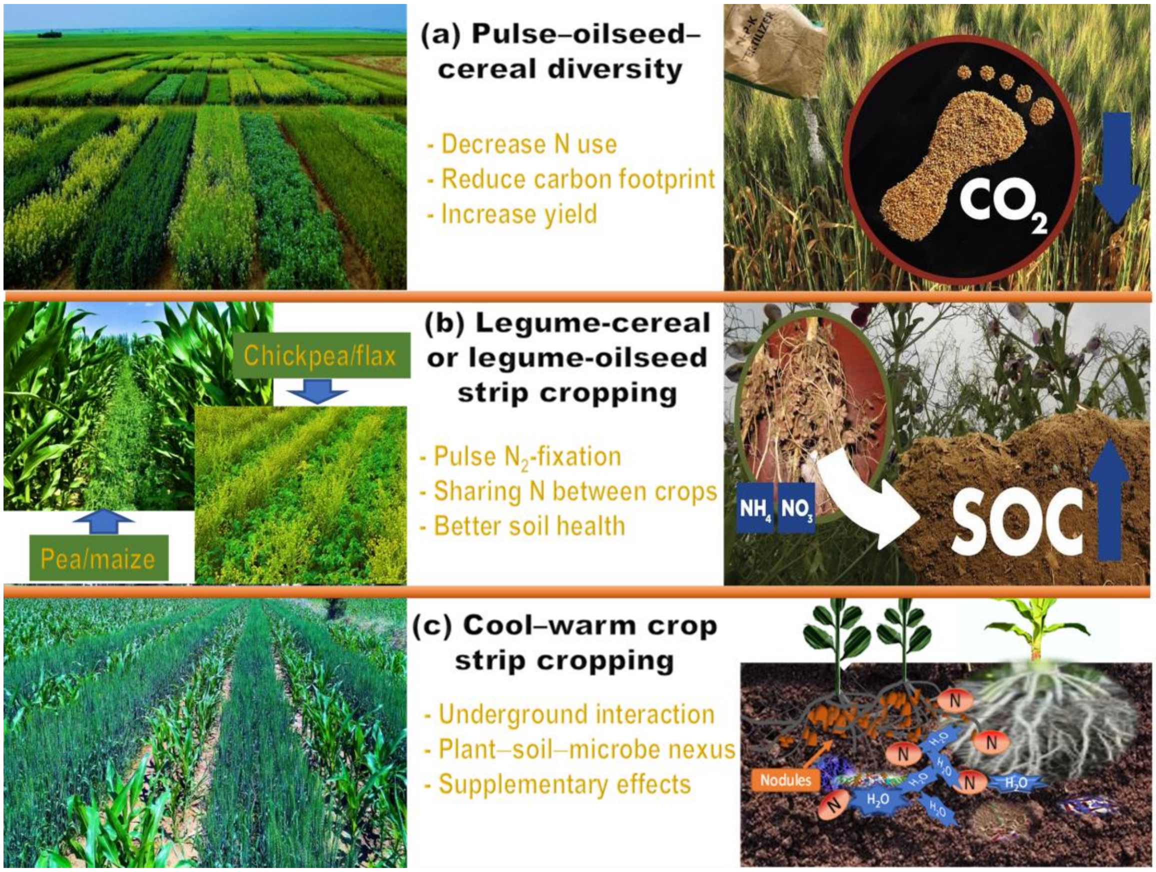

Figure S3. Legume-based cropping diversification provides significant benefits to soil health and environmental sustainability. For smallholders in less developed countries with smaller plots of land, legume-based rotation diversification offers an additional income source and can act as a safety net given low capacities to respond in the case of extreme weather events.

Legume-based diversification provides significant benefits to soil health. A meta-analysis with global data sources (77 articles, 393 treatments) revealed that crop diversification coupled with reduced or no tillage increased soil fungi abundance and fungi-to-bacteria ratios, favoring nutrient cycling and improving soil health. In the Indo-Gangetic Plains, incorporating annual legumes such as chickpea into maize–wheat and rice–wheat rotations increased SOC by 7–13%, particulate C by 41–95%, and labile C by 29–38% as compared to cereal-based monoculture. In temperate southwest France, temporally-grown legumes between cash crops lowered CO_2 emissions by 50–102% compared to a

cropping system without legumes. Shifting cropping priorities from traditional cereal-dominant systems to legume-based diversified systems can produce more plant-based protein, supporting a growing trend towards plant-based proteins globally.

Several meta-analyses that synthesized numerous field experiments revealed inconsistent effects. A meta-analysis of studies conducted across Mediterranean, tropical, subtropical and temperate monsoon climates in Spain and China showed that no-till (NT) with returning crop residues increased N_2O emissions by 6.1 to 12.9%[18]. Similarly, another global meta-analysis revealed that no-till increased CO_2, N_2O, and CH_4 emissions by 7.1, 12.0, and 20.8%, respectively, as compared to conventional tillage (CT)[19]. Increased N fertilization rates under NT management improved crop yield and GHGs emissions up to 23 and 58%, respectively, compared to CT.

Conversely, a meta-analysis with 151 direct comparisons between NT or reduced tillage (RT) and CT showed that NT/RT decreased soil N_2O emissions by 11% compared to CT in humid areas and in soils with carbon content < 20 g kg^{-1}[20].

A literature review of 37 published papers showed tillage intensity did not affect cumulative N_2O even after 38–40 years of tillage implementation, regardless of the use of a moldboard plow, chisel plow, double disk, or no-till practices[21].

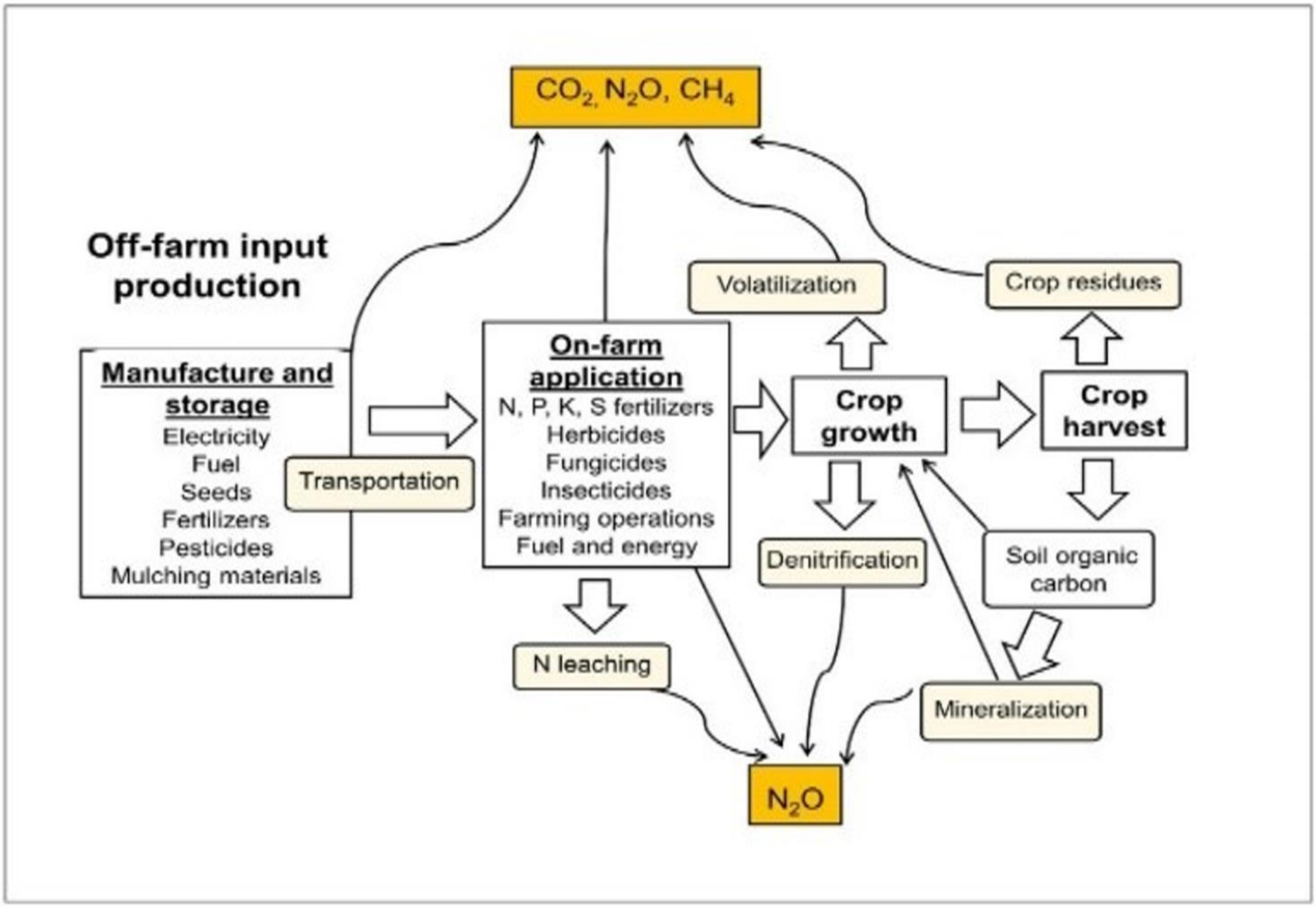

Figure S4. The boundary for estimating CO_2eq emissions in agrifood production system. The emissions include those from energy use and non-energy sources, with N_2O and CH_4 emissions converted to CO_2eq as defined in the IPCC guideline. The estimate formula was adapted and modified from Yang et al. (2024).

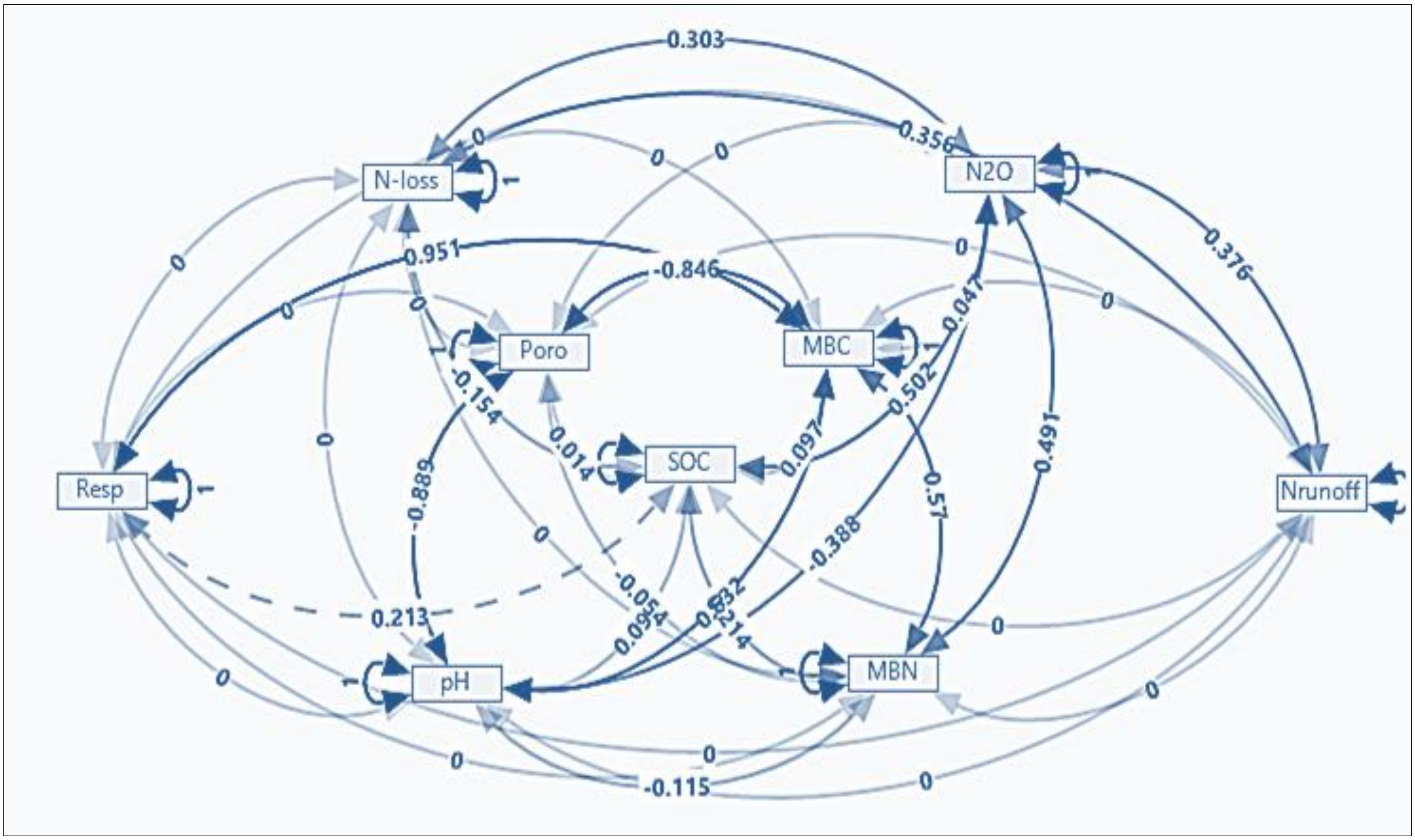

301

Figure S5. Structural equation modeling indicates that N_2O emissions during cropping phases are the main contributors to overall N loss. These emissions are positively correlated with N runoff (Nrunoff), whereas the total N loss is complicated by the interactive effect between soil porosity (poro) and microbial biomass carbon (MBC), MBC and microbial biomass nitrogen (MBN), N_2O and pH and porosity, and soil pH and SOC. Less important factors to the three goals have been excluded from the figure based on their correlation coefficients (the numbers besides the lines).

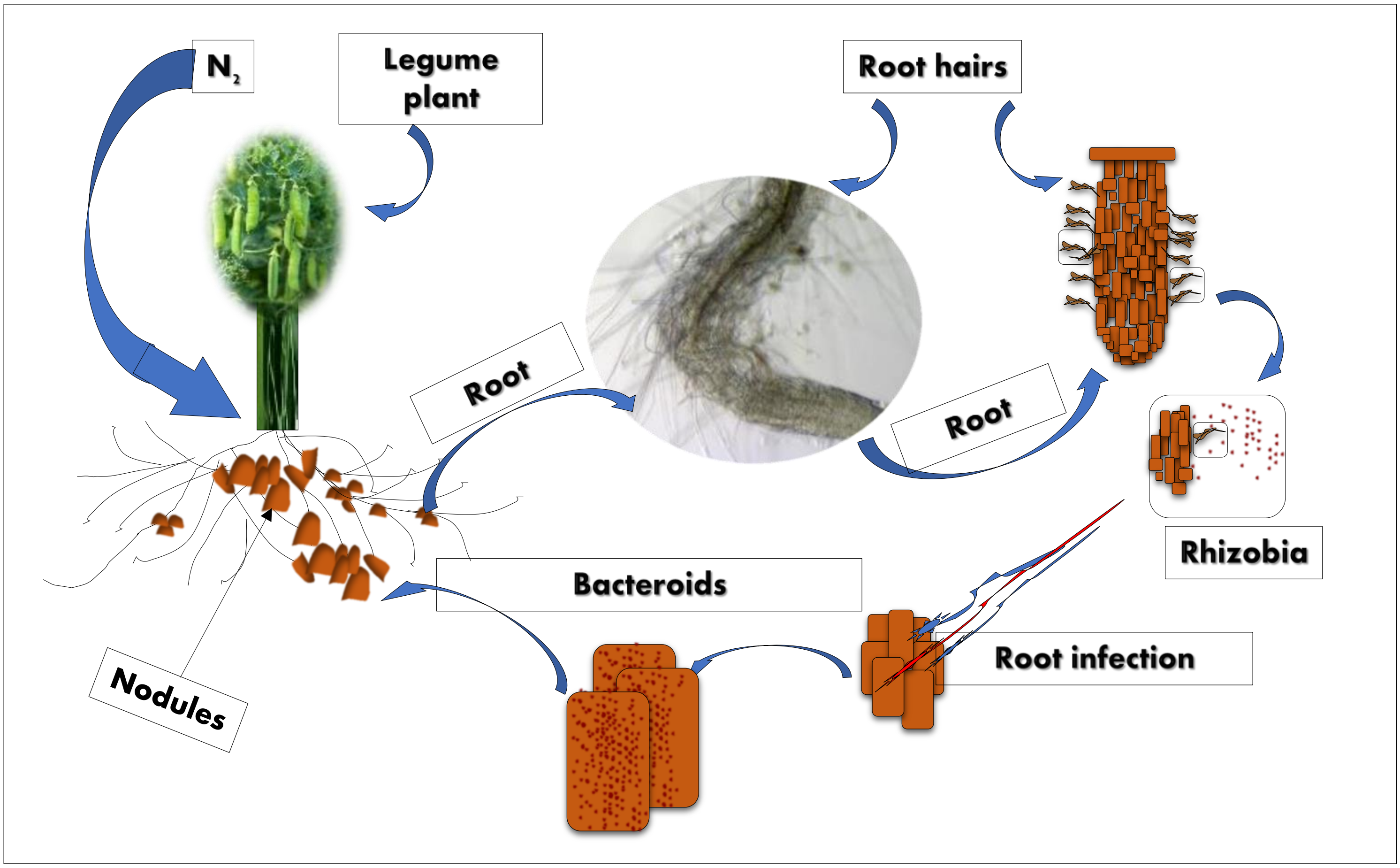

302

Figure S6. Rhizobial symbiosis in legume plant roots. Rhizobacteria enter the roots through root hairs to form rhizobia, and then go through root infection become bacteriods, and forms nodules on the infected sites on the root. Many nodules can be formed in the roots of legume plants to develop a mutual relationship between the bacterioids and root cells, and the symbiosis converts atmospheric N_2 into plant-available N. The symbiosis promotes ammonia secretion by bacteroides through regulating oxygen supply and catabolizing plant-derived dicarboxylates through root hairs as the energy and electron source donors for N_2 fixation, leading to conversion of atmospheric N_2 into NH_3.

303

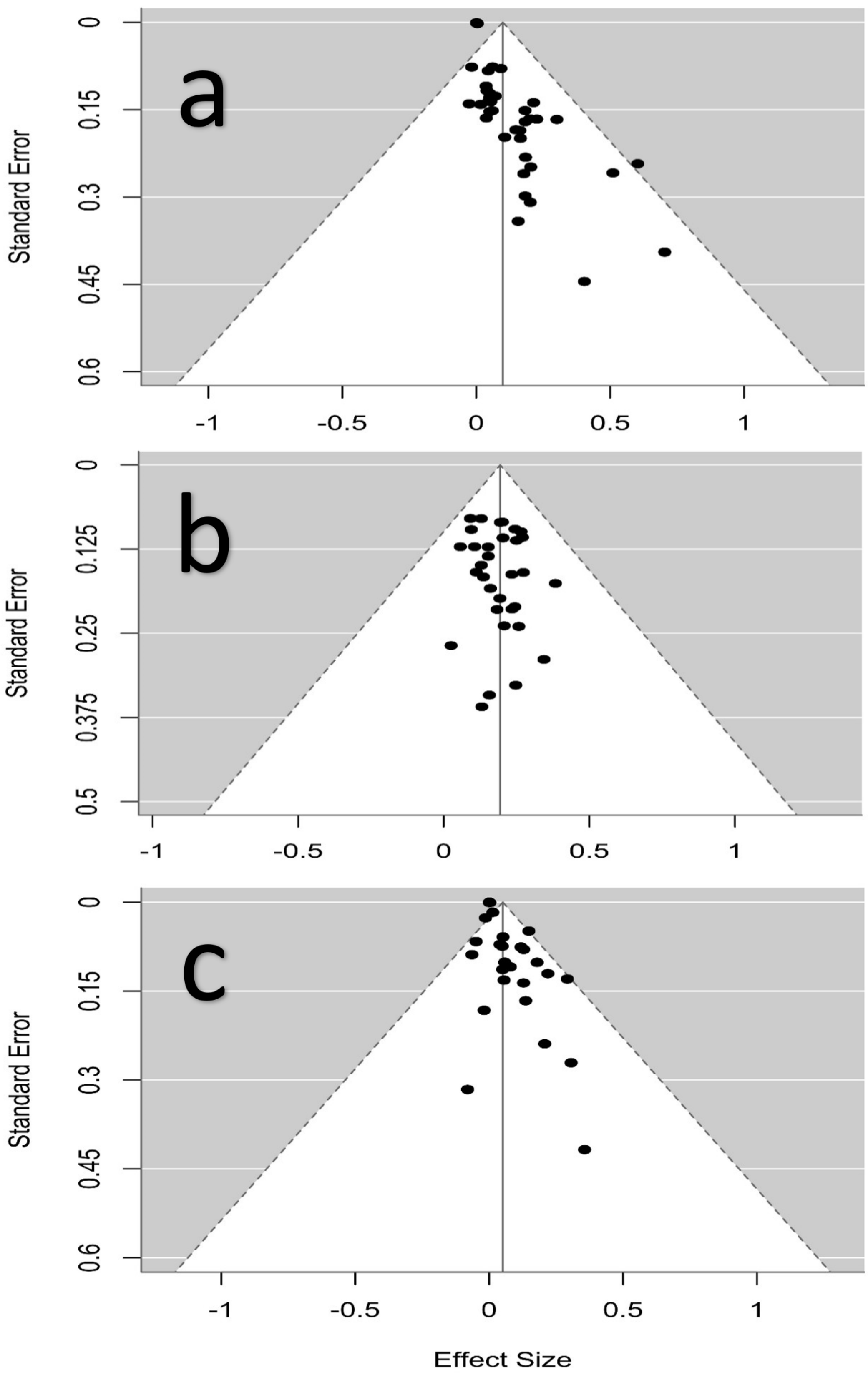

Figure S7. Funnel plots for (**a**) more food, (**b**) healthy soil, and (**c**) fewer emissions.
